# Description of strongly heat-inducible *heat shock protein 70* transcripts from Baikal endemic amphipods

**DOI:** 10.1038/s41598-019-45193-0

**Published:** 2019-06-20

**Authors:** Polina Drozdova, Daria Bedulina, Ekaterina Madyarova, Lorena Rivarola-Duarte, Stephan Schreiber, Peter F. Stadler, Till Luckenbach, Maxim Timofeyev

**Affiliations:** 1 Irkutsk State University, Institute of Biology, Irkutsk, 664003 Russia; 2 Baikal Research Centre, 664003 Irkutsk, Russia; 3 Leipzig University, Interdisciplinary Center for Bioinformatics, Leipzig, D-04107 Germany; 4 Leipzig University, Bioinformatics Group, Department of Computer Science, Leipzig, D-04107 Germany; 5 UFZ – Helmholtz Centre for Environmental Research, Young Investigators Group Bioinformatics and Transcriptomics, Leipzig, D-04318 Germany; 6 Leipzig University, LIFE-Leipzig Research Center for Civilization Diseases and Competence Center for Scalable Data Services and Solutions, Leipzig, D-04107 Germany; 7 University of Vienna, Institute for Theoretical Chemistry, Wien, A-1090 Austria; 8 University of Copenhagen, Center for non-coding RNA in Technology and Health, Frederiksberg C, DK-1870 Denmark; 9 Facultad de Ciencias, Universidad National de Colombia, Sede Bogotá, COL-111321 Colombia; 10 Santa Fe Institute, Santa Fe, NM87501 USA; 11 UFZ – Helmholtz Centre for Environmental Research, Department of Bioanalytical Ecotoxicology, Leipzig, D-04318 Germany; 12 Present Address: Plant Genome and Systems Biology, Helmholtz Zentrum München, Neuherberg, D-85764 Germany

**Keywords:** Transcriptomics, Freshwater ecology

## Abstract

Heat shock proteins/cognates 70 are chaperones essential for proper protein folding. This protein family comprises inducible members (Hsp70s) with expression triggered by the increased concentration of misfolded proteins due to protein-destabilizing conditions, as well as constitutively expressed cognate members (Hsc70s). Previous works on non-model amphipod species *Eulimnogammarus verrucosus* and *Eulimnogammarus cyaneus*, both endemic to Lake Baikal in Eastern Siberia, have only revealed a constitutively expressed form, expression of which was moderately further induced by protein-destabilizing conditions. Here we describe heat-inducible *hsp70s* in these species. Contrary to the common approach of using sequence similarity with *hsp/hsc70* of a wide spectrum of organisms and some characteristic features, such as absence of introns within genes and presence of heat shock elements in their promoter areas, the present study is based on next-generation sequencing for the studied or related species followed by differential expression analysis, quantitative PCR validation and detailed investigation of the predicted polypeptide sequences. This approach allowed us to describe a novel type of *hsp70* transcripts that overexpress in response to heat shock. Moreover, we propose diagnostic sequence features of this Hsp70 type for amphipods. Phylogenetic comparisons with different types of Hsp/Hsc70s allowed us to suggest that the *hsp/hsc70* gene family in Amphipoda diversified into cognate and heat-inducible paralogs independently from other crustaceans. Thus, the cognate and inducible *hsp70* types in distant taxa may not be recognized by sequence similarity.

## Introduction

The 70 kDa heat shock protein (*hsp70*) multigene family encodes proteins localized in different compartments of eukaryotic cells such as cytosol, endoplasmic reticulum, mitochondria, and plastids^1^. According to^2,3^, the members of the *hsp70* family are functionally divided into three groups: (1) solely constitutive (heat-shock cognates, *hsc70*), which provide folding of polypeptides under normal physiological conditions^4^; (2) solely inducible (*hsp70*), which are absent under normal physiological conditions and considerably up-regulated in response to proteotoxic stressors^5^; and (3) cognate-inducible (*hsp/hsc70*), which are expressed under normal physiological conditions and up-regulated in response to stress^6^. Thus, this protein family can be further referred to as the Hsp/Hsc70 family.

The number of *hsp/hsc70* family members, as well as the functional role of particular orthologs, varies significantly across taxa, and this variation may play a role in evolutionary adaptation to different environmental conditions^7^. Despite some features determined for the inducible members of the *hsp/hsc70* family (absence of introns in the corresponding genes, presence of specific heat shock elements in their promoters, as well as specific amino acid signatures and sequence similarity with other inducible forms^8^), no clear evidence for consistent interspecies identification of inducible form has been presented so far^3^.

All Hsp/Hsc70s consist of an N-terminal ATPase domain (nucleotide binding domain, NBD, 44 kDa), a substrate binding domain (SBD, 18 kDa) and a variable C-terminus (10 kDa)^9^. Their chaperone activity is controlled by interactions with co-chaperones and nucleotide exchange factors (NEFs), which determine nucleotide status and thus substrate-binding activity, via specific sites located mostly in the NBD and the C-terminus^10,11^. Hsp/Hsc70s act as molecular chaperones involved in the folding and transport of newly synthesized polypeptides across membranes and thus play important roles in many vital processes like apoptosis, cell proliferation and development, senescence and the immune response^8,12,13^.

As various chemical stress factors strongly induce *hsp70* transcripts/Hsp70 proteins, changes in the transcript levels or protein titers are used as molecular biomarkers in environmental monitoring^14,15^. As the *hsp70* genes have highly conserved sequences and structures across animal taxa, quantitative PCR (qPCR) primers can be designed relatively easily. Using changes in the *hsp70* expression level as a comprehensive biomarker in environmental monitoring requires fast and reliable detection of the strong increase in the mRNA concentration (two to three orders of magnitude increase compared to the control level). This pattern was observed in many model objects, as well as in some non-model organisms and *in vitro* systems (*Drosophila melanogaster* Meigen, 1830, *Saccharomyces cerevisiae* Meyen ex E.C. Hansen, *Xenopus laevis* Daudin, 1802, *Caenorhabditis elegans* (Maupas, 1900), mammalian cell cultures). However, recent research in an expanding number of non-model organisms revealed that in many cases the high level of *hsp/hsc70* expression is observed in the non-stressed (control) conditions, whereas stress conditions induce *hsp/hsc70* gene expression only slightly (two- to five-fold) or not at all^16,17^. These observations make some researchers emphasize cautions in applying these proteins to non-model organisms due to the high complexity of the *hsp/hsc70* multigene family^3^.

The number of *hsp70* paralogs varies between taxa and even lineages within one species with an average of ten^16^. Multiple isoforms of *hsc70* genes have also been described^16^. Even if the *hsp70* copies within a genome seem to be recent paralogs, their function in stress response may vary significantly from no effect to rapid and strong up-regulation^3,18–20^. One of the best examples illustrating this phenomenon is the specific expression pattern and functional properties of *hsp/hsc70* in Antarctic and Arctic species^3,8^. Without deeper consideration of this functional variability, erroneous interpretations are often possible^3^.

Amphipods (Amphipoda: Crustacea), comprising 354 endemic species and subspecies^21^, are one of the most diverse groups by species number and essential elements in the benthic food web of the ancient Lake Baikal and are therefore essential for evolutionary, eco-physiological research and ecotoxicity monitoring^22^. In our recent studies, we showed only a weak (up to two-fold) response in *hsp/hsc70* expression to different thermal treatments in two Baikal endemic amphipods *Eulimnogammarus verrucosus* (Gerstfeldt, 1858) and *E. cyaneus* (Dybowsky, 1874) using both qPCR and Northern hybridization^23^.

The weak up-regulation of the *hsp/hsc70* forms described to date in Baikal amphipods precludes their use in environmental monitoring of Lake Baikal. However, considering the complex evolution of the *hsp/hsc70* multigene family, it has been proposed that the key characteristics for determination of inducible and cognate forms of these proteins can vary significantly in different taxa^24^. Thus, it is essential to identify the heat-inducible forms of *hsp70* in amphipods. As demonstrated in the recent studies of krill, next-generation sequencing and whole-transcriptome differential gene expression analysis can serve as the best method to identify the most appropriate for biomonitoring *hsp70* orthologs^18,25,26^.

In this work, we aimed to identify the inducible *hsp70* in Baikal endemic amphipods using next-generation sequencing and whole-transcriptome differential gene expression analysis followed by qPCR validation and sequence analysis. Our results allow us to propose a method for rapid determination of heat-inducible *hsp70* in non-model taxa based on the specific structural characteristics of a predicted polypeptide on the example of amphipods. Based on the applied approach, we recommend using expression data for the studied or related organism to choose target sequences for qPCR and similar applications for biomonitoring in non-model organisms.

## Results

### Differential expression analysis of the *hsp/hsc70* multigene family

**Figure 1:**
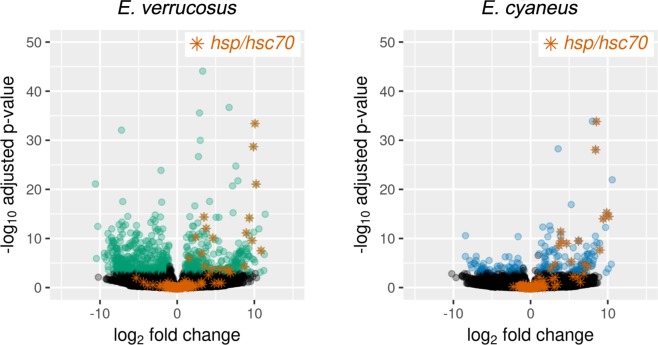
Volcano plots of *hsp/hsc70* transcripts in amphipods upon exposure to increased temperature. Depicted are fold changes in transcript levels and associated p-values. Differentially expressed transcript levels (absolute log2 fold change >1 and adjusted p-value < 0.001) are indicated by green (for *E. verrucosus*) or blue (for *E. cyaneus*) symbols; black symbols indicate non-significant changes in expression levels. Transcripts annotated as *hsp/hsc70* are marked with orange asterisks.

In the transcriptomes of the specimens of both species from the 24 h heat shock treatments, a variety of transcripts were found that were highly similar to *heat shock protein 70/71* of crustaceans or other metazoans. Twenty-three out of 155 transcripts for *E. verrucosus* and 19 out of 108 transcripts for *E. cyaneus* were significantly (p < 0.001, fold change >2) up-regulated (up to 1900-fold for *E. verrucosus* and up to 1100-fold for *E. cyaneus* (Fig. 1; Supplementary Table 1).

**Figure 2:**
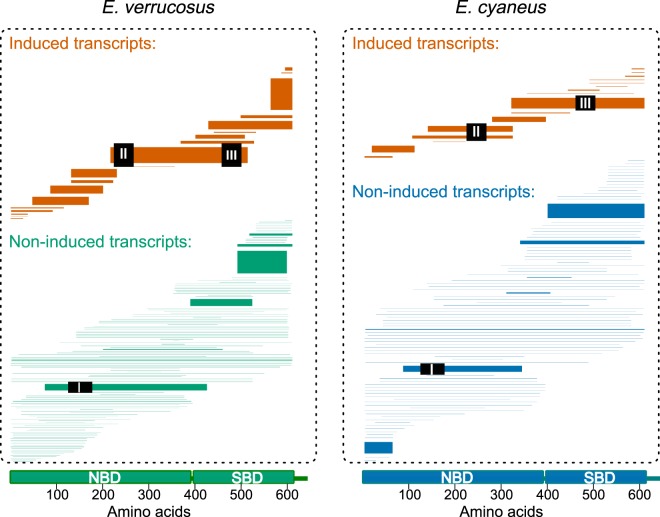
Graphical representation of Hsp/Hsc70 protein sequence alignment shows that most *hsc70* transcripts and all *hsp70* transcripts are represented by fragmented contigs. The position of the transcript contigs represents the region of the reference sequence (at the bottom of the figure) to which the contigs are aligned. The domain structure of the reference protein sequence is based on the alignment with the human HSP1A (P0DMV8), the domain structure of which is shown according to^65^. The transcripts with unchanged expression are shown in green for *E. verrucosus* and in blue for *E. cyaneus*, while the induced ones are shown in orange. The line width is proportional to the relative abundance of the corresponding transcript (transcripts per million).

All transcripts were aligned to the Hsp70b protein sequences previously identified^27^ for both amphipod species and used as reference sequences here (AEX65805 for *E. verrucosus* and AEX65807 for *E. cyaneus*) with blastx^28^. Fig. 2 shows a graphical representation of the alignments of each identified contig to the reference protein sequence; the width of each line is proportional to the overall abundance of reads for this contig. Interestingly, *de novo* assemblies resulted in contigs representing full-length transcripts only found for *hsc70* but not for *hsp70*. This result might be connected to higher variability between putative inducible *hsp70s*.

Further analysis of these transcripts showed that only one transcript in each species contained binding sites for the primer pair used previously^23,27^ (primer pair I as shown in Fig. 2). Neither of these transcripts was differentially expressed in our transcriptomic experiments.

Moreover, most *hsp/hsc70* transcripts had low abundances (quantities of mapped reads) across all samples. This fact might reflect either their low expression level or some assembly errors. However, twelve transcripts in *E. verrucosus* and ten transcripts in *E. cyaneus* were characterized by mean expression above 200 transcripts per million (or at least about 500 reads per transcript), and about half of them were induced by heat shock (Fig. 2, orange lines). Characteristics of these transcripts are summarized in Supplementary Table 2, and contig sequences are available as Supplementary Data 1. Interestingly, some of these transcripts represented reverse complement sequences. They may reflect imperfect strand specificity of the analyzed RNA sequencing library or presence of reverse complement transcripts and require further investigation.

### qPCR validation

To test the existence and differential expression of the specific heat-inducible *hsp70* transcript, we designed primers amplifying the most abundant induced transcript(s) (either pair of primers matched both *E. verrucosus* and *E. cyaneus* sequences; primer pairs II and III in Fig. 2).

**Figure 3:**
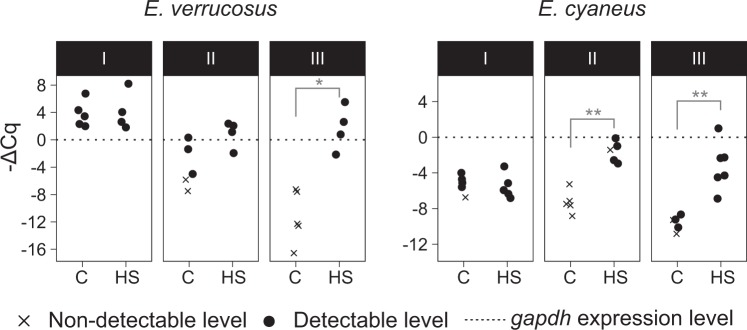
Relative expression of *hsp/hsc70* fragments amplified with different primer pairs. Quantification cycle (ΔCq) was calculated relative to the glyceraldehyde 3-phosphate dehydrogenase (*gapdh*) gene. C, control (6 °C). HS, heat shock (24.5 °C for *E. verrucosus* or 25.5 °C for *E. cyaneus*). *p < 0.05; **p < 0.01 (Mann-Whitney test with Holm correction for multiple comparisons).

Indeed, while the abundance of the fragment amplified by 5′-most pair (I) did not differ between the experimental and control groups, the abundances of the fragments amplified with primer pairs II and III were significantly higher under heat shock for one or both species (Fig. 3). The expression of these fragments in the control group was undetectable in most control samples. These data corroborate the RNA sequencing results and indicate that we have found an heat-inducible *hsp70*.

### Diversity of the putative *hsp70s* in amphipods

**Figure 4:**
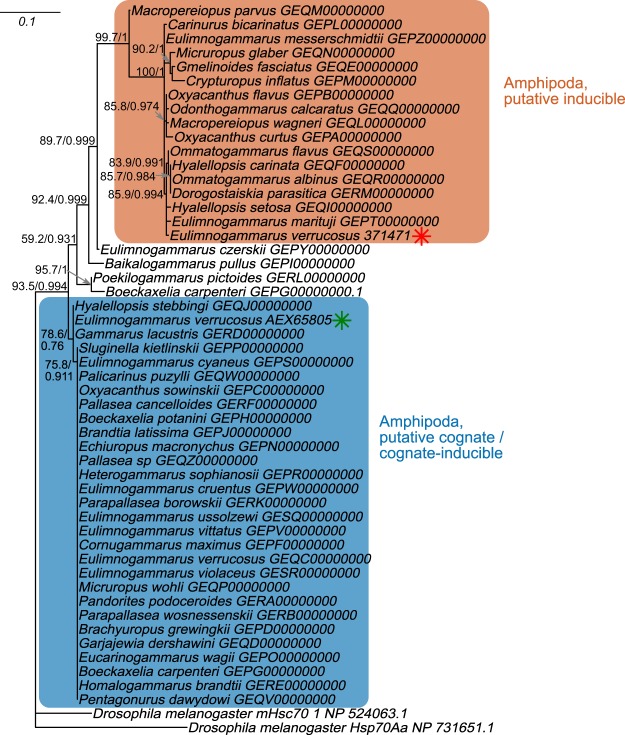
Maximum likelihood tree of 289 amino acid long sequences (corresponding to amino acids 217–514 in the reference *E. verrucosus* and *E. cyaneus* sequences) that constitute the best match for the heat-inducible Hsp70 of *E. verrucosus* in the corresponding transcriptomes of Baikal amphipods published by Naumenko *et al*.^29^. Systematic assignments are provided as in^29^. Shimodaira-Hasegawa approximate likelihood ratio test (SH-aLRT)/approximate Bayes (aBayes) support values for the clade are shown if one of them was above 70% or 0.7, respectively. *Drosophila melanogaster* sequences serve as outgroups.

In order to further characterize the putative heat-inducible *hsp70*, we used the predicted amino acid sequence of the longest of the major heat-inducible transcripts in *E. verrucosus* (298 amino acids) as a query to search for similar sequences in the 66 available transcriptomes of Baikal amphipods^29^ and recovered at least one such sequence in 48 of them. In 28 species we found the sequences similar to the Hsp/Hsc70 from *E. verrucosus* published earlier^23^ (Fig. 4, the upper part of the tree). However, in 16 species we found sequences similar to the heat-inducible Hsp70 we describe here. Besides, in four species we found different new *hsp/hsc70* sequences, which did not cluster within the two main described groups; however, fragments of *hsp/hsc70* found in *Eulimnogammarus czerskii* (Dybowsky, 1874) and *Baikalogammarus pullus* (Dybowsky, 1874) were similar to the clade of the heat-inducible Hsp70s (approx. 90% identity of either sequence to the consensus sequence of this group). In two species—*Poekilogammarus pictoides* Sowinsky, 1915 and *Boeckaxelia carpenterii* (Dybowsky, 1874)—a putatively distinct type of sequences was found, which is more similar to the described cognate-inducible consensus sequence (96% and 94% similarity, respectively) than to the heat-inducible Hsp70 fragment (91% and 90% similarity for *P. pictoides* and *B. carpenterii* sequences, respectively) in Baikal amphipods (Fig. 4).

### Gene model generation and structural analysis of *hsp/hsc70* sequences

To further characterize the transcript, a gene model containing the complete open reading frame (ORF) was generated by mapping transcriptome reads to the corresponding sequence of *Ommatogammarus flavus* (Dybowsky, 1874) ORF, as it was the closest relative to the *Eulimnogammarus* genus among all species in which we found the putative heat-inducible *hsp70*, according to Naumenko *et al*.^29^. The obtained seven consensus ORFs contained 1926 base pairs, corresponding to 641 amino acids, and were 98.6% identical to each other except for amino acid positions 5 (G/S), 83 (S/T), 171 (L/R), 250 (Y/F), 316 (D/E), 363 (T/S), 413 (T/A), 559 (L/V) and 633 (A/T). The obtained model included the nucleotide binding domain (amino acids 1–390), the substrate binding domain (amino acids 397–560) and the C-terminus (amino acids 561–641); it also possessed the major amino acid signatures characteristic for the cytosolic Hsp/Hsc70 (see below).

**Figure 5:**
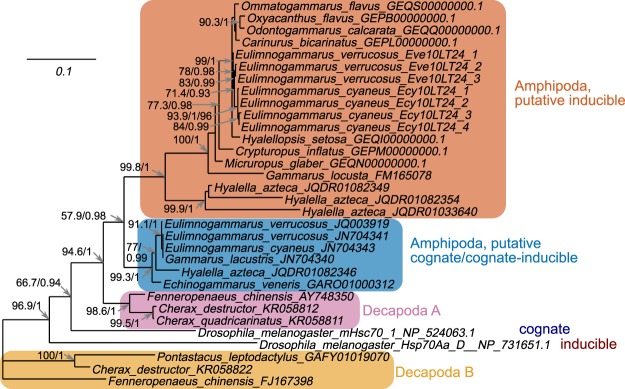
Maximum likelihood tree of all available full-length Hsp/Hsc70 protein sequences from amphipods and decapods. SH-aLRT/aBayes support values for the clade are shown if one of them was above 70% or 0.7, respectively. Sequences of some Decapoda species of group A are omitted for clarity. Sequences in bold are from this work. The alignment and phylogenetic tree used to make this figure are available as Supplementary Data 2 and 3, respectively.

We combined the obtained ORF sequences (eight full-length sequences obtained from the published transcriptomes of Baikal amphipods, three consensus sequences of *E. verrucosus* samples and four consensus sequences of *E. cyaneus* samples described above) with all available Amphipoda and Decapoda full-length *hsp/hsc70* sequences from a list published earlier^30^ to create a phylogenetic tree (Supplementary Data 3; Fig. 5).

The phylogenetic tree based on the complete ORFs exhibited a similar topology within amphipods as the tree based on the fragment of the protein sequence. Decapod sequences were divided into groups A and B, as described by Baringou *et al*.^30^, whereas all Amphipoda sequences formed a sister clade to the Decapoda A group of sequences. All *hsp/hsc70* sequences from Amphipoda were divided into two clades, one of each included all sequences similar to heat-inducible transcripts detected in this study. Sequences derived from the assembled genome of *Hyalella azteca* (Saussure, 1858) (JQDR00000000.1) included at least three sequences similar to heat-inducible *hsp70s*. The second clade of *hsp/hsc70* sequences included putative cognate or cognate/inducible forms, two of which from the studied species were described previously in^23^. Besides, the available in NCBI *hsp/hsc70* sequences from *Gammarus lacustris* (G. O. Sars, 1863), *Echinogammarus veneris* (Heller, 1865) and one gene containing an *hsp/hsc70* ORF from the genome assembly of *H. azteca* were clustered in this group.

**Figure 6:**
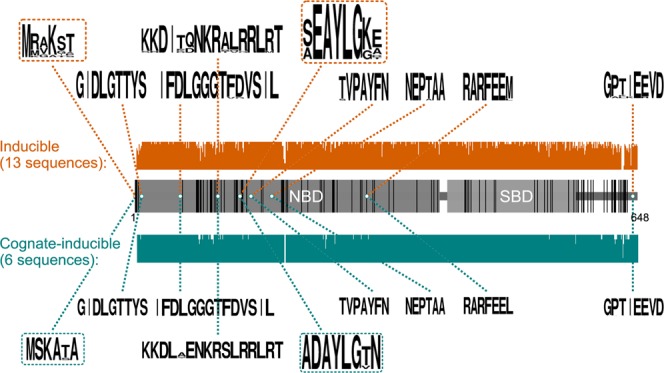
A schematic indicating the main features and conservative regions of putatively inducible (orange) and cognate/cognate-inducible (teal) Hsp70 proteins. Bar heights on the upper and lower panels correspond to % conservation in the alignment. Black bars on the middle panel designate positions that are different between consensus heat-inducible and putative cognate/cognate-inducible sequences. NBD, nucleotide-binding domain; SBD, substrate-binding domain. See the two top frames in Fig. 5 for the list of species in each group and Supplementary Data 2 for the full protein sequences. Sequence logos were generated with WebLogo (http://weblogo.berkeley.edu/logo.cgi).

For a more in-depth structural analysis, all cognate/cognate-inducible and all heat-inducible sequences from Amphipoda were used to make two consensus sequences. Interestingly, the inducible forms were more variable (Fig. 6). The N-terminal sequence was variable between these two forms with MSKATA in almost all putative cognate sequences and MRAKST being the most frequent sequence in the putative heat-inducible Hsp70s. The amino acids from 6/7 to 77/78 are almost identical in all amphipod Hsp/Hsc70 sequences analyzed, while the remaining part of the NBD contains variable sites. Moreover, three out of four *H. azteca* sequences contain an insertion of two amino acids in positions 191–192. It is worth mentioning that the specific amino acid signature of the ATP-binding site is profoundly different between the two putative orthologs: (S(A)EAYLGK(G)E(A) for Hsp70 and ADAYLGTN for Hsc70). All major Hsp/Hsc70-specific sequence signatures have been found in all sequences (Fig. 6): IDLGTTYS, TVPAYFND, NEPTAA^31,32^, the PO4–binding site IFDLGGGTFDVSIL^33^ (PROSITE ID HSP70_2 PS00329), and the putative nuclear localization signal KKXXXXXXXXXRRLRT, which participates in the translocation of Hsp70 into the nucleus under heat shock^34^. The specific signatures RARFEEL (for putative cognate form) and RARFEEM (for the described heat-induced form) and GPTIEEVD (in the C-terminal)^35^ are the evidence of the cytosolic localization of the obtained sequences. The putative eukaryotic ATP/GTP-binding site was found with the sequence ADAYLGTN for the putative cognate form and S(A)EAYLGK(G)E(A) for the described inducible form.

### Analysis of *hsp/hsc70* genes in the genome of *H. azteca*

As the presence of heat shock elements (HSEs) in the promoter area and absence of introns is sometimes used to characterize the inducible *hsp/hsc70* family member, we analyzed the gene structures and the promoter areas in the available genome of the amphipod *H. azteca* (JQDR00000000.1). Three *H. azteca* genomic scaffolds containing full-length *hsp/hsc70* homologs were retrieved from the arthropod *hsp/hsc70* database created by Baringou *et al*.^30^. None of these genes contained introns. One scaffold (JQDR01082354.1, 14767 bp) included two inverted copies similar to *hsp70* described in this study, one of which did not have EEVD signature, indicating its non-cytosolic localization. Two more scaffolds (JQDR01082349.1, 6839 bp and JQDR01033640.1, 34444 bp) included only one copy of sequences similar to *hsp70* and one more (JQDR01082346.1, 19421 bp), which was similar to the *hsp/hsc70* described in this study.

The promoter areas of the described *H. azteca hsp/hsc70* sequences have several distant HSEs and HSE-like elements. The canonical HSE contains at least three continuous repeats of pentanucleotides NTTCN and NGAAN, which are necessary for the binding of heat shock factors trimer^36^. In the heat-inducible forms of *H. azteca*, we found an HSE-like sequence AATCGAGAAATTCTGGAAG located 1187 bp upstream of the ORF start, and one HSE TTTCATGAACTTTCC located in 1565 bp upstream of the start codon in the inverted non-cytoplasmic *hsp70* (JQDR01033640.1); and CTTCTGGAACATCCA located 1159 bp upstream the start codon (JQDR01033640.1). The putative *hsp/hsc70* contained two canonical HSEs, AGAAAGTTCTAGAAC, located in 2380 bp upstream to the start codon, and TGAAATTTCCTGAA, located in 3178 bp upstream to the promoter area (JQDR01082346.1). We did not find any canonical HSEs or HSE-like sequences within the promoter area of the gene similar to the heat-inducible *hsp70* (JQDR01082349.1).

## Discussion

Previously, we have described two orthologs of *hsp/hsc70*, namely *hsp/hsc70a* and *hsp/hsc70b*, from the genus *Eulimnogammarus*. Based on their inducibility in response to acute and chronic thermal exposure measured by qPCR and Northern hybridization, these orthologs were classified as weakly inducible, as their expression was up-regulated up to 2.5-fold^23^. In the present study, using next-generation sequencing coupled with qPCR validation, sequence similarity analysis with other *hsp/hsc70s* and examination of conservative sequence regions, we detected *hsp70* transcripts that were up to 1900-fold up-regulated after 24 hours of heat shock exposure in these *Eulimnogammarus* species.

Further phylogenetic analysis revealed that the sequences of this type are members of the heat-inducible *hsp70* subfamily in all amphipods. These findings corroborate the results of Hauton *et al*.^37^, who identified in the amphipod *Gammarus locusta* (Linnaeus, 1758) an *hsp/hsc70* family member similar to the heat-inducible form described in this study that upon acute heat shock was up to 2000-fold up-regulated. Recent studies on the evolution of cytosolic *hsp/hsc70s* revealed that heat-inducible forms have evolved independently more than once in different species, suggesting convergent evolution^24^. Earlier, Baringou *et al*.^30^ demonstrated the same pattern on *hsp/hsc70* within Malacostraca. It was proposed that at least two different orthologous groups of cytosolic *hsp/hsc70* exist within Malacostraca. Specific amino acid signatures defining these putative orthologous groups (designated as A and B) have been described. Nevertheless, no distinctive pattern of inducible *hsp70* in Arthropods was found in this study^30^. At the same time, Baringou *et al*.^30^ suggested that amphipods should have “a more complex evolutionary scenario for *hsp/hsc70*” within the *hsp/hsc70* A group.

Our data supported the finding of Baringou *et al*.^30^, as our previously described putative cognate/cognate-inducible and heat-inducible forms of *hsp/hsc70* from the amphipod cluster within the *hsp/hsc70* A group. We can, therefore, propose that the amphipod *hsp/hsc70s* evolved from an ancestral gene forming *hsp/hsc70* group of Malacostraca with the following functional divergence into cognate (weakly inducible) and heat-inducible forms.

The phylogenetic tree based on the central fragment of the ORF retrieved from the full transcriptome sequences of 66 amphipod species indicates that the evolution of *hsp/hsc70* in amphipods does not correlate with species evolution. According to the sequence similarity analysis, multiple orthologous groups of both the *hsp/hsc70* family members may exist in amphipods. We found that the diversity of Hsp/Hsc70 cognate/inducible sequences is lower than that of the inducible Hsp70s sequences. This difference can indicate high conservation of Hsp/Hsc70 forms, as they play a vital role in normal cell physiology, being involved in housekeeping functions such as protein folding and transport. Contrary, heat-inducible forms are highly variable and may have arisen as recent paralogs in different species via gene duplication and conversion, which is a known feature of molecular evolution of the *hsp70* genes and an adaptation to specific environmental conditions in many taxa^7^. Interestingly, as only animals acclimated under laboratory conditions (*i.e*., non-stressed individuals) were used to obtain the transcriptomes, we would expect to find mostly putative cognate/cognate-inducible sequences; however, the heat-inducible forms were found not only, but mostly in deep-water species, which could experience stress after retrieval from deeper zones.

Despite the high similarity of the predicted amino acid sequences of Hsp/Hsc70 polypeptides, distinct substitutions, and even indels were described for these forms in amphipods. The functional meaning of the described differences requires additional investigation; however, it may reflect different activities of Hsp/Hsc70 proteins as molecular chaperones. The ATP-binding site (S(A)EAYLGK(G)E(A)), which we have described for the heat-inducible forms, is more similar to the canonical for Hsp70 in various taxa (AEAYLGQK(P)^35^) than to the one previously described for the putative cognate/cognate-inducible Hsp70 (ADAYLGTN^23^). Based on our phylogenetic analysis, we can propose this amino acid motive along with the MRAKST as diagnostic features for the heat-inducible Hsp70 determination in amphipods.

Other potential indicators are sometimes used to identify or to validate the stress-inducible *hsp70s*, namely the presence of heat shock elements and the absence of introns. HSEs are necessary for the heat shock factor (HSF) trimers binding to enhance stress-inducible *hsp70* expression^36,38^. However, our results have demonstrated that HSEs can be present/absent in the promoter area independently of the functional role of the *hsp/hsc70* family member. In our previously described genomic sequences of *hsp/hsc70*, we have found HSE and HSE-like near the putative cognate-inducible forms in both studied species^23^. In the *H. azteca* genome HSEs and HSE-like sequences were found both in the promoter area of genes similar to the here described cognate and inducible forms; however, they were located in more distant positions from the start codon, than in both *Eulimnogammarus* species. The role of such distant HSEs in facilitating gene expression is still unclear; however, we can conclude that the presence of HSE in promoters of *hsp/hsc70* genes should not be used as an indicator of *hsp70* in poorly investigated non-model organisms.

The absence/presence of introns also does not indicate if the *hsp/hsc70* gene is heat-inducible or cognate in amphipods. It was suggested that the lack of introns in the heat-inducible form allowed for a more rapid response to stress because mRNA splicing is not necessary^38^. According to the study of Baringou *et al*.^30^, a single intron is present only in the form B1 in Decapoda, whereas no intron was found in *hsp/hsc70* form A and B2. In our previous study, no introns were found in the putative cognate-inducible *hsp/hsc70* in the studied species^23^. Moreover, Qin *et al*.^39^ found introns in stress-inducible *hsp70* genes in the African migratory locust *Locusta migratoria* (Linnaeus, 1758). Introns exist in the heat inducible *hsp70* genes in the nematode *Caenorhabditis elegans* (Maupas, 1900)^40^. Thus, the role of introns in *hsp/hsc70* requires additional research, and the presence or absence of introns cannot be used to determine stress-inducible family members.

The previous study demonstrated the existence of multiple gene copies and putative pseudogenes among the *hsp/hsc70* family members, which vary significantly in closely related endemic Baikal amphipod species with different thermal tolerance^23^. Thus, a deeper investigation of this gene family and functional role of its members in evolution and adaptation can shed light on the peculiarity of micro-evolution and adaptation of different organisms to their microhabitats. Furthermore, these studies are critical to developing comprehensive molecular biomarkers for environmental monitoring of Lake Baikal and other aquatic ecosystems using amphipods.

In this work, we describe a novel type of heat-inducible *hsp70* transcripts in Lake Baikal endemic amphipods and propose distinctive sequence features to diagnose the heat-inducible Hsp70 form for the order Amphipoda. Our data highlight the importance of considering the particular *hsp70* sequence for designing primers or probes for precise qPCR, microarray or similar analyses. The approach used here can be instrumental in the characterization of a heat-inducible *hsp70* for environmental monitoring assays in different non-model organisms. Moreover, our phylogenetic analysis suggests that the *hsp/hsc70* gene family within the order Amphipoda diversified into cognate and heat-inducible paralogs independently from other crustaceans. Thus, the cognate and inducible *hsp70* types in distant taxa may not be recognized only by sequence similarity.

## Methods

### Animals and experiments

*Eulimnogammarus verrucosus* (Gerstfeldt, 1858) and *Eulimnogammarus cyaneus* (Dybowsky, 1874) are amphipod species endemic to Lake Baikal. The polyphyletic genus *Eulimnogammarus* belongs to the phylogenetic clade Acanthogammaridae^29^. Adult *E. verrucosus* individuals have body lengths of 20–50 mm. This species is one of the most abundant amphipods of the macrozoobenthos community along the stony littoral and sub-littoral zones at water depths of 0–25 m^41^. It reproduces during winter months and is stenotherm at low temperatures^22,23,42^. The body lengths of adult *E. cyaneus* (Dybowsky, 1874) are 11–15 mm. This species inhabits rocky shores reaching the highest population density in the shallow water. It reproduces in summer and was shown to be relatively thermotolerant^22,23,42^.

The amphipod individuals were collected with a hand net from 0.5–1.5 m. The animals used for the RNA sequencing experiments were collected in August 2013 (the water temperature at sampling varied between 12–16 °C) near the village Bolshie Koty (south-west coast of Baikal 51 54′11.67″N 105 4′7.61″E). Immediately after sampling, the amphipods were transferred to the laboratory for pre-acclimation for one week at 6 ± 1 °C in well-aerated Baikal water. Individuals of *E. verrucosus* for qPCR experiments were collected in April 2018 in Listvyanka (south-west coast of Baikal 51 52′14.07″N, 104 49′41.78″E) at 0.6 °C and acclimated for three weeks at 6 ± 1 °C. Individuals of *E. cyaneus* for qPCR experiments were collected in June 2018 near the Bolshie Koty village at the water temperature of 9 °C and acclimated in the lab for one week at 6 ± 1 °C. During acclimation, the amphipods were fed *ad libitum* with dried and ground invertebrates and algae from their habitat, and water was exchanged every three to four days. No mortality was observed during acclimation.

For the heat shock exposure experiments, the individuals were exposed at 24.8 ± 0.7 °C for 24 hours, while parallel controls were kept at 6 ± 1 °C (acclimation temperature) for the same time. After 24 hours the animals were quickly frozen in liquid nitrogen. The heat shock temperature was chosen to be close to the 50% lethality values determined for these species earlier, namely 23.5 °C (standard deviation = 6.9) for *E. verrucosus* and 25.5 °C (standard deviation = 3.2) for *E. cyaneus*^23^. In our experiments, the overall average mortality during the exposure was 17% and 15% for *E. verrucosus* and *E. cyaneus*, respectively, while median values across all preliminary experiments were equal to 10% for both species. No mortality was observed in parallel controls. More detailed information on mortality under heat shock treatment can be found in Supplementary Table 3.

All applicable international, national, and institutional guidelines for the care and use of animals were followed. The research was approved by the Animal Subjects Research Committee of the Institute of Biology at Irkutsk State University.

### RNA extraction and quantitative qPCR analysis

Total RNA for RNA sequencing was extracted from a single individual in the case of *E. verrucosus* and from a pool of four to five specimens in the case of *E. cyaneus* (up to 700 mg). Amphipods were homogenized in 1 ml of Qiazol Reagent (Qiagen) using an MM400 homogenizer (Retsch). RNA isolation from the aqueous phases was performed using a Qiacube (Qiagen) with the miRNeasy kit (Qiagen), then mRNA was isolated from the total RNA sample using the Oligotex mRNA Mini Kit (Qiagen). The quality of RNA was checked with the RNA 6000 Nano Kit on an Agilent Bioanalyzer 2100. Sequencing libraries were prepared with the ScriptSeqTM v2 RNA-Seq Library Preparation Kit (Epicentre, # SSV21124). Total RNA for qPCR analysis was extracted from single individuals of *E. verrucosus* or *E. cyaneus* by homogenizing frozen animals in TriReagent (MRC) according to the protocol provided by the manufacturer with 3 and 5 mm stainless steel beads (Qiagen) in a TissueLyser LT (Qiagen). The homogenate was centrifuged to remove the beads and mixed with chloroform according to the protocol of the manufacturer; centrifugation and phase separation steps were done in MaxTract high-density tubes (Qiagen). Total RNA was then isolated with the RNeasy Mini kit (Qiagen) according to the protocol, and the samples were treated with the RNase-free DNase set (Qiagen) according to the instructions in Appendix B of the miRNeasy Mini Handbook 12/2014, and RNA integrity was checked with agarose gel electrophoresis.

Complementary DNA (cDNA) synthesis was performed with Reverta (Interlabservice) or with Thermo Scientific RevertAid RT Kit RevertAid FirstStrand cDNA synthesis (Thermo Scientific) kit with random primers according to the recommendation of the manufacturers, and up to 5% volume of the cDNA synthesis reaction was used as a template for qPCR. Amplification was performed with a StepOnePlus (Applied Biosystems) using the SensiMix Hi-Rox reagents (Bioline). The same amount of the RNA sample treated the same way but without adding reverse transcriptase (-RT) was used to test for genomic DNA contamination. Primers used in this experiment are listed in Supplementary Table 4; the glyceraldehyde 3-phosphate dehydrogenase gene (*gapdh*) was used as a reference gene. Experimental (cDNA) samples with quantification cycles over 30 or -RT samples with those below 30 for *gapdh* were considered below detection or contaminated, respectively, and discarded from further analysis.

### Next-generation sequencing and data analysis

RNA sequencing was performed with a HiSeq. 2000 (Illumina) in three to four replicates for each species and condition (approximately 30 million 2 × 100-bp reads per sample). Transcriptome assemblies were based on 16–18 samples for each species. Before the assembly, raw reads were trimmed with trim_galore^43^ v0.4.1 to remove sequencing adapters, filtered with bowtie v2.1.0^44^ against a custom-made database of Gammaridae rRNA sequences and were corrected with rcorrector^45^ (cloned on 18th December 2017). Quality control at each stage was performed with FastQC^46^ v0.11.5 and MultiQC^47^ v1.2. At the next step, reads were assembled with Trinity v2.4.0^48^. The resulting assemblies were annotated with diamond^49^ v0.9.10.111 by mapping to the non-redundant NCBI protein sequence database (the version of 10th October 2017).

Sequencing reads were mapped to assembled and annotated transcriptomes of each species with salmon^50^ v0.9.1. The data were imported into R^51^ with the tximport package^52^, and differential expression was estimated with a custom script based on the DESeq2 package^53^ v1.14.1. The ggplot2 package^54^ v3.0.0 was used for plotting. Only transcripts with a best hit to heat shock protein 70/71 from some metazoan species with calculated p-values (155 *E. verrucosus* transcripts and 108 *E. cyaneus* transcripts) were selected for subsequent analysis. Raw sequencing reads, assemblies and mapping statistics are available from the GEO NCBI database (GSE122811).

The qPCR data were represented as relative quantitative cycles (ΔCq), obtained by subtraction of the quantitative cycle value for the reference gene from the corresponding value of the target gene. The samples of ΔCq values for at least four biological replicates for each condition (control or treatment) were compared with the Wilcoxon-Mann-Whitney test implemented in the coin package^55^ with Holm correction for multiple comparisons^56^ as implemented in the base R package.

Alignment of the assembled transcript sequences to the reference Hsp/Hsc70 protein sequences for the corresponding species was performed with standalone blastx^28^ v2.2.28 + . Search for the transcripts encoding the amino acid sequence specific for the heat-inducible Hsp70 was performed with exonerate^57^ v2.2.0. Multiple sequence analysis was performed with prank^58^ v170427 with default settings, and phylogenetic trees were built with IQ-Tree^59^ with default settings except for the addition of 10,000 Shimodaira-Hasegawa approximate likelihood ratio test (SH-aLRT) generations and calculation of posterior probabilities with the approximate Bayes (aBayes) test^60^. The ggtree package^61^ v1.12.7 was used to visualize the phylogenetic trees.

To generate gene models for the heat-inducible *hsp70*, we aligned all reads to the *Ommatogammarus flavus* (Dybowsky, 1874) ORF sequence with bowtie2^44^ v2.1.0 with subsequent filtering for properly mapped reads only (-f147, -f99) with samtools^62^ v0.1.19-96b5f2294. The resulting alignments were manually inspected for sufficient coverage, and the consensus sequences were generated with UGENE^63,64^ v1.30.0.

## Supplementary information

Supplementary material

## Data Availability

RNA sequencing and differential expression data are available from the NCBI GEO repository (GEO: GSE122811). All other data are included in this article and Supplementary Materials.
