# Supplementary material for: Description of strongly heat-inducible heat shock protein 70 transcripts from Baikal endemic amphipods
Source: Sci Rep. 2019 Jun 20;9:8907. doi: 10.1038/s41598-019-45193-0 (PMC6586656; doi:10.1038/s41598-019-45193-0)
Supplement: Supplementary file 1 — Supplementary material [file 41598_2019_45193_MOESM1_ESM.pdf]

Supplementary Information

Article Title:

Description of strongly heat-inducible *heat shock protein 70* transcripts from Baikal endemic amphipods

Authors:

Polina Drozdova[1,+], Daria Bedulina[1,2,+], Ekaterina Madyarova[1,2], Lorena Rivarola-Duarte[3,4,a], Stephan Schreiber[5], Peter F. Stadler[3,4,6-10], Till Luckenbach[11], and Maxim Timofeyev[1,2,*]

[1] Irkutsk State University, Institute of Biology, Irkutsk, 664003, Russia
[2] Baikal Research Centre, 664003, Irkutsk, Russia
[3] Leipzig University, Interdisciplinary Center for Bioinformatics, Leipzig, D-04107, Germany
[4] Leipzig University, Bioinformatics Group, Department of Computer Science, Leipzig, D-04107, Germany
[5] UFZ – Helmholtz Centre for Environmental Research, Young Investigators Group Bioinformatics and Transcriptomics, Leipzig, D-04318, Germany
[6] Leipzig University, LIFE-Leipzig Research Center for Civilization Diseases and Competence Center for Scalable Data Services and Solutions, Leipzig, D-04107, Germany
[7] University of Vienna, Institute for Theoretical Chemistry, Wien, A-1090, Austria
[8] University of Copenhagen, Center for non-coding RNA in Technology and Health, Frederiksberg C, DK-1870, Denmark
[9] Facultad de Ciencias, Universidad National de Colombia, Sede Bogotá, COL-111321, Colombia
[10] Santa Fe Institute, Santa Fe, NM87501, USA
[11] UFZ – Helmholtz Centre for Environmental Research, Department of Bioanalytical Ecotoxicology, Leipzig, D-04318, Germany
* m.a.timofeyev@gmail.com
[+] these authors contributed equally to this work
a Current Address: Plant Genome and Systems Biology, Helmholtz Zentrum München, Neuherberg, D-85764, Germany

Table of Contents

Supplementary Table 1. Differential expression of contigs annotated as hsp/hsc70....................................2
Supplementary Table 2. Characteristics of the most abundant hsp/hsc70 transcripts in the transcriptome assemblies....................................7
Supplementary Table 3. Mortality under heat shock....................................9
Supplementary Table 4. Oligonucleotides used in this work....................................10
Supplementary Data 1. Nucleotide sequences of contigs in Supplementary Table 2....................................11
Supplementary Data 2. Multiple sequence alignment for Fig. 5....................................15
Supplementary Data 3. Phylogenetic tree for Fig. 5....................................28

Supplementary Table 1. Differential expression of contigs annotated as *hsp/hsc70*. *E. verrucosus*, 24 h heat shock / control

Contig name	baseMean	log2FoldChange	padj	log10padj	Best nr hit
TRINITY_DN352603_c0_g3_i1	4.57	-5.31	0.011	1.973	XP_021360006.1 heat shock cognate 71 kDa protein [Mizuhopecten yessoensis]
TRINITY_DN351897_c2_g3_i1	3.62	-4.37	0.078	1.109	ABU89874.1 heat shock protein 70, partial [Ecteinascidia turbinata]
TRINITY_DN374290_c6_g5_i1	2.29	-3.84	0.320	0.495	ACD75767.1 heat shock cognate 70 [Chanodichthys ilishaeformis]
TRINITY_DN373011_c1_g2_i1	9.02	-3.14	0.291	0.536	KMQ97439.1 heat shock 70 kda protein cognate 4-like protein [Lasius niger]
TRINITY_DN380216_c2_g1_i10	3.58	-2.27	0.731	0.136	XP_019549197.1 PREDICTED: heat shock 70 kDa protein cognate 4 [Aedes albopictus]
TRINITY_DN373264_c1_g1_i2	2.94	-2.18	0.226	0.647	AIZ00749.1 heat shock cognate 70 protein, partial [Sesamia inferens]
TRINITY_DN380216_c2_g1_i9	5.67	-1.57	0.874	0.058	XP_019549197.1 PREDICTED: heat shock 70 kDa protein cognate 4 [Aedes albopictus]
TRINITY_DN380216_c2_g1_i8	43.86	-1.53	0.520	0.284	XP_019549197.1 PREDICTED: heat shock 70 kDa protein cognate 4 [Aedes albopictus]
TRINITY_DN350520_c4_g3_i3	6.13	-1.42	0.253	0.597	XP_007435687.1 PREDICTED: heat shock-related 70 kDa protein 2 [Python bivittatus]
TRINITY_DN380216_c2_g1_i5	18.57	-1.34	0.854	0.068	XP_019549197.1 PREDICTED: heat shock 70 kDa protein cognate 4 [Aedes albopictus]
TRINITY_DN370094_c4_g1_i3	8.07	-1.33	0.218	0.661	XP_015788504.1 PREDICTED: heat shock cognate 71 kDa protein-like [Tetranychus urticae]
TRINITY_DN350520_c4_g2_i2	9.06	-1.26	0.489	0.311	XP_003708652.1 PREDICTED: heat shock 70 kDa protein cognate 4 isoform X3 [Megachile rotundata]
TRINITY_DN351423_c1_g1_i4	2.43	-1.24	0.613	0.213	XP_017877146.1 PREDICTED: heat shock 70 kDa protein cognate 4-like [Ceratina calcarata]
TRINITY_DN378842_c0_g1_i1	140.33	-1.18	0.088	1.055	XP_017460793.1 PREDICTED: heat shock 70 kDa protein 1A-like isoform X2 [Rhagoletis zephyria]
TRINITY_DN372576_c0_g1_i11	3.89	-1.15	0.548	0.261	XP_012146197.1 PREDICTED: heat shock cognate 71 kDa protein-like isoform X2 [Megachile rotundata]
TRINITY_DN373264_c0_g1_i1	6.87	-1.15	0.503	0.298	XP_020468828.1 heat shock 70 kDa protein 1-like [Monopterus albus]
TRINITY_DN360827_c0_g3_i1	3.11	-1.07	0.799	0.098	CDS42930.1 heat shock protein 70 [Echinococcus multilocularis]
TRINITY_DN380832_c1_g1_i1	6.62	-1.02	0.594	0.227	CUT99763.1 heat shock protein 70 [Echinococcus multilocularis]
TRINITY_DN377277_c4_g1_i6	5.66	-0.99	0.945	0.025	AAG47839.1 heat shock protein 70 [Heterodera glycines]
TRINITY_DN350520_c4_g3_i4	3.98	-0.96	0.689	0.162	XP_019762664.1 PREDICTED: heat shock 70 kDa protein cognate 4-like [Dendroctonus ponderosae]
TRINITY_DN372576_c0_g4_i1	17.93	-0.94	0.396	0.402	XP_012255464.1 heat shock 70 kDa protein cognate 4-like [Athalia rosae]
TRINITY_DN358737_c4_g1_i2	111.78	-0.90	0.825	0.083	XP_018019096.1 PREDICTED: heat shock 70 kDa protein 14-like, partial [Hyalella azteca]
TRINITY_DN378216_c0_g2_i1	15.95	-0.76	0.758	0.120	XP_008321770.1 PREDICTED: heat shock cognate 71 kDa protein-like [Cynoglossus semilaevis]
TRINITY_DN360827_c0_g1_i8	14.44	-0.75	0.898	0.047	XP_009998966.1 PREDICTED: heat shock-related 70 kDa protein 2 [Chaetura pelagica]
TRINITY_DN350052_c0_g3_i1	2.69	-0.74	0.814	0.090	ABA02165.1 heat shock protein 70 [Homarus americanus]
TRINITY_DN372046_c0_g2_i1	9.57	-0.68	0.671	0.173	XP_004479764.1 PREDICTED: heat shock 70 kDa protein 6 [Dasypus novemcinctus]
TRINITY_DN351423_c1_g1_i15	7.75	-0.64	0.837	0.077	XP_017877146.1 PREDICTED: heat shock 70 kDa protein cognate 4-like [Ceratina calcarata]
TRINITY_DN354431_c2_g3_i1	52.18	-0.61	0.323	0.491	XP_022055649.1 heat shock 70 kDa protein 1 [Acanthochromis polyacanthus]
TRINITY_DN372576_c0_g1_i4	25.70	-0.59	0.641	0.193	XP_012146197.1 PREDICTED: heat shock cognate 71 kDa protein-like isoform X2 [Megachile rotundata]
TRINITY_DN371955_c0_g1_i3	2.99	-0.59	0.969	0.013	AAF87583.1 heat shock 70 protein [Parastrongyloides trichosuri]
TRINITY_DN363756_c1_g1_i4	11.32	-0.59	0.686	0.163	AMR60410.1 heat shock protein 70 [Sinanodonta woodiana]
TRINITY_DN375937_c7_g2_i1	26.48	-0.58	0.818	0.087	XP_018017811.1 PREDICTED: heat shock 70 kDa protein II-like [Hyalella azteca]
TRINITY_DN380216_c2_g1_i4	61.18	-0.57	0.901	0.045	XP_019549197.1 PREDICTED: heat shock 70 kDa protein cognate 4 [Aedes albopictus]
TRINITY_DN368994_c0_g1_i1	34.18	-0.56	0.967	0.014	BAO23806.1 heat shock 70 kDa protein 1 [Brachionus plicatilis]
TRINITY_DN354305_c2_g1_i4	4.42	-0.53	0.871	0.060	XP_022662585.1 heat shock cognate 71 kDa protein-like [Varroa destructor]
TRINITY_DN378567_c1_g1_i3	1046.53	-0.53	0.483	0.316	AFI60316.1 heat shock protein 70 [Eulimnogammarus verrucosus]
TRINITY_DN354313_c2_g2_i1	58.83	-0.52	0.774	0.111	ABA02164.1 heat shock protein 70 [Pachygrapsus marmoratus]
TRINITY_DN374774_c1_g4_i2	2.30	-0.48	0.923	0.035	XP_022643827.1 heat shock-related 70 kDa protein 2-like [Varroa destructor]
TRINITY_DN375872_c1_g3_i1	21.78	-0.44	0.708	0.150	XP_018521434.1 PREDICTED: heat shock 70 kDa protein-like [Lates calcarifer]
TRINITY_DN377277_c1_g1_i1	6.49	-0.44	0.821	0.086	XP_022691208.1 heat shock 70 kDa protein-like [Varroa jacobsoni]
TRINITY_DN378842_c0_g1_i2	2.75	-0.40	0.932	0.030	XP_004671914.1 PREDICTED: heat shock 70 kDa protein 1B [Jaculus jaculus]
TRINITY_DN362833_c0_g1_i1	18.82	-0.38	0.830	0.081	XP_019562417.1 PREDICTED: heat shock 70 kDa protein cognate 4-like [Aedes albopictus]
TRINITY_DN354305_c2_g1_i6	14.80	-0.38	0.871	0.060	XP_022662585.1 heat shock cognate 71 kDa protein-like [Varroa destructor]
TRINITY_DN368994_c0_g1_i2	77.38	-0.35	0.969	0.014	BAO23806.1 heat shock 70 kDa protein 1 [Brachionus plicatilis]
TRINITY_DN347208_c2_g1_i1	13.59	-0.32	0.902	0.045	ACJ03595.1 heat shock protein 70 [Hypophthalmichthys molitrix]
TRINITY_DN351802_c0_g2_i7	3.20	-0.32	0.943	0.025	XP_006772899.1 PREDICTED: heat shock 70 kDa protein 1-like [Myotis davidii]
TRINITY_DN362493_c1_g1_i6	21.91	-0.31	0.882	0.055	XP_015753655.1 PREDICTED: heat shock cognate 71 kDa protein-like [Acropora digitifera]
TRINITY_DN376172_c0_g1_i7	35.53	-0.23	0.922	0.035	XP_017135651.1 PREDICTED: heat shock 70 kDa protein cognate 1 [Drosophila miranda]
TRINITY_DN362765_c0_g2_i2	202.18	-0.21	0.937	0.028	ERG83796.1 heat shock protein 70 b2 [Ascaris suum]
TRINITY_DN370191_c0_g1_i9	15.93	-0.21	0.965	0.015	AFD62317.1 heat shock protein 70, partial [Styela plicata]
TRINITY_DN376921_c1_g1_i1	17.54	-0.18	0.932	0.031	XP_014237218.1 PREDICTED: heat shock 70 kDa protein cognate 4-like [Trichogramma pretiosum]

TRINITY_DN373264_c1_g1_i4	119.93	-0.18	0.898	0.047	XP_022528366.1 heat shock 70 kDa protein [Astyanax mexicanus]
TRINITY_DN370745_c0_g2_i3	2.10	-0.17	0.971	0.013	XP_018017811.1 PREDICTED: heat shock 70 kDa protein II-like [Hyalella azteca]
TRINITY_DN375175_c0_g1_i2	21.85	-0.17	0.918	0.037	CAQ60114.1 70kDa heat shock protein [Gammarus locusta]
TRINITY_DN372576_c0_g1_i6	3.32	-0.13	0.985	0.006	XP_012146197.1 PREDICTED: heat shock cognate 71 kDa protein-like isoform X2 [Megachile rotundata]
TRINITY_DN378566_c0_g1_i3	5.23	-0.11	0.979	0.009	XP_005627220.1 heat shock 70 kDa protein 1-like isoform X1 [Canis lupus familiaris]
TRINITY_DN365031_c0_g1_i3	9.87	-0.08	0.993	0.003	XP_022643827.1 heat shock-related 70 kDa protein 2-like [Varroa destructor]
TRINITY_DN374774_c1_g4_i3	5.98	-0.02	0.995	0.002	BAO23807.1 heat shock 70 kDa protein 2 [Brachionus plicatilis]
TRINITY_DN378567_c1_g1_i5	366.96	0.00	0.999	0.000	AFI60316.1 heat shock protein 70 [Eulimnogammarus verrucosus]
TRINITY_DN379719_c0_g1_i2	2.28	0.01	0.998	0.001	XP_018007836.1 PREDICTED: heat shock cognate 71 kDa protein-like [Hyalella azteca]
TRINITY_DN363756_c1_g1_i1	2.73	0.04	0.994	0.002	XP_018610869.1 PREDICTED: heat shock cognate 71 kDa protein [Scleropages formosus]
TRINITY_DN376921_c1_g1_i2	2.34	0.04	0.997	0.001	XP_004522113.1 heat shock 70 kDa protein cognate 1 [Ceratitis capitata]
TRINITY_DN377277_c4_g1_i1	5.79	0.04	0.997	0.001	AAG47839.1 heat shock protein 70 [Heterodera glycines]
TRINITY_DN380832_c1_g3_i1	4.23	0.10	0.979	0.009	ABP93403.1 heat shock cognate 70 protein [Omphisa fuscidentalis]
TRINITY_DN378567_c1_g1_i2	2281.25	0.12	0.934	0.030	AFI60316.1 heat shock protein 70 [Eulimnogammarus verrucosus]
TRINITY_DN379719_c0_g1_i4	2.75	0.12	0.983	0.008	XP_018007836.1 PREDICTED: heat shock cognate 71 kDa protein-like [Hyalella azteca]
TRINITY_DN380671_c0_g1_i2	41.65	0.13	0.952	0.021	BAO23807.1 heat shock 70 kDa protein 2 [Brachionus plicatilis]
TRINITY_DN424227_c0_g1_i1	2.19	0.14	0.985	0.006	KPM08853.1 Sar s 28 (heat shock protein 70-like protein 5) [Sarcoptes scabiei]
TRINITY_DN378567_c1_g2_i2	3121.75	0.20	0.762	0.118	AFI60316.1 heat shock protein 70 [Eulimnogammarus verrucosus]
TRINITY_DN370094_c4_g1_i1	39.87	0.21	0.890	0.051	XP_015788504.1 PREDICTED: heat shock cognate 71 kDa protein-like [Tetranychus urticae]
TRINITY_DN357424_c0_g2_i1	134.15	0.22	0.887	0.052	XP_006772899.1 PREDICTED: heat shock 70 kDa protein 1-like [Myotis davidii]
TRINITY_DN354305_c2_g1_i5	2.10	0.24	0.977	0.010	XP_022662585.1 heat shock cognate 71 kDa protein-like [Varroa destructor]
TRINITY_DN374774_c1_g1_i1	12.33	0.25	0.901	0.045	XP_018803158.1 PREDICTED: heat shock 70 kDa protein cognate 1 [Bactrocera latifrons]
TRINITY_DN351802_c0_g2_i2	5.36	0.27	0.954	0.020	XP_006772899.1 PREDICTED: heat shock 70 kDa protein 1-like [Myotis davidii]
TRINITY_DN365302_c3_g1_i1	8.58	0.28	0.908	0.042	XP_007064665.1 PREDICTED: heat shock 70 kDa protein 1-like [Chelonia mydas]
TRINITY_DN375872_c1_g2_i4	10.69	0.29	0.952	0.021	XP_017313781.1 PREDICTED: heat shock 70 kDa protein 1 [Ictalurus punctatus]
TRINITY_DN371955_c0_g1_i2	24.32	0.30	0.809	0.092	AAF87583.1 heat shock 70 protein [Parastrongyloides trichosuri]
TRINITY_DN362493_c1_g3_i2	4.57	0.34	0.944	0.025	XP_004522113.1 heat shock 70 kDa protein cognate 1 [Ceratitis capitata]
TRINITY_DN363756_c1_g1_i2	6.80	0.39	0.895	0.048	XP_010952055.1 PREDICTED: heat shock 70 kDa protein 6 [Camelus bactrianus]
TRINITY_DN358737_c4_g1_i1	112.12	0.40	0.794	0.100	XP_018019096.1 PREDICTED: heat shock 70 kDa protein 14-like, partial [Hyalella azteca]
TRINITY_DN377277_c4_g1_i2	18.62	0.42	0.853	0.069	AAF87583.1 heat shock 70 protein [Parastrongyloides trichosuri]
TRINITY_DN373028_c0_g2_i2	14.83	0.43	0.774	0.111	XP_022643829.1 LOW QUALITY PROTEIN: heat shock cognate 71 kDa protein-like [Varroa destructor]
TRINITY_DN378559_c4_g3_i1	19.17	0.46	0.815	0.089	NP_001274490.1 heat shock-related 70 kDa protein 2 [Pelodiscus sinensis]
TRINITY_DN355554_c0_g1_i1	850.47	0.47	0.607	0.217	XP_018012862.1 PREDICTED: heat shock 70 kDa protein cognate 5-like [Hyalella azteca]
TRINITY_DN379719_c2_g1_i2	72.60	0.48	0.546	0.263	BAO23807.1 heat shock 70 kDa protein 2 [Brachionus plicatilis]
TRINITY_DN372576_c0_g1_i3	7.27	0.52	0.906	0.043	BAO23806.1 heat shock 70 kDa protein 1 [Brachionus plicatilis]
TRINITY_DN379150_c1_g1_i1	4.06	0.54	0.877	0.057	XP_022644917.1 heat shock 70 kDa protein 1-like [Varroa destructor]
TRINITY_DN377357_c2_g1_i4	7.32	0.55	0.861	0.065	AAN78300.1 heat shock protein 70 A [Heterodera glycines]
TRINITY_DN370191_c0_g3_i1	2.12	0.55	0.919	0.037	CAQ60114.1 70kDa heat shock protein [Gammarus locusta]
TRINITY_DN347332_c2_g1_i1	833.04	0.56	0.297	0.527	AFI60316.1 heat shock protein 70 [Eulimnogammarus verrucosus]
TRINITY_DN374497_c0_g1_i4	2.00	0.56	0.922	0.035	XP_015797521.1 PREDICTED: heat shock 70 kDa protein 1 [Nothobranchius furzeri]
TRINITY_DN377277_c4_g1_i4	14.74	0.58	0.808	0.093	AAG47839.1 heat shock protein 70 [Heterodera glycines]
TRINITY_DN366648_c5_g1_i2	14.37	0.58	0.817	0.088	XP_011829864.1 PREDICTED: heat shock cognate 71 kDa protein-like isoform X1 [Mandrillus leucophaeus]
TRINITY_DN351802_c0_g2_i1	3.29	0.62	0.898	0.047	XP_006772899.1 PREDICTED: heat shock 70 kDa protein 1-like [Myotis davidii]
TRINITY_DN350052_c0_g2_i1	12.71	0.67	0.817	0.088	AGH32327.1 heat shock protein 70 [Cellana toreuma]
TRINITY_DN351286_c0_g3_i1	2.98	0.68	0.805	0.094	XP_011200265.1 PREDICTED: heat shock 70 kDa protein cognate 1 [Bactrocera dorsalis]
TRINITY_DN360827_c0_g1_i4	9.16	0.71	0.912	0.040	AFI60316.1 heat shock protein 70 [Eulimnogammarus verrucosus]
TRINITY_DN378216_c0_g2_i3	42.66	0.72	0.482	0.317	XP_014784774.1 PREDICTED: heat shock cognate 71 kDa protein-like [Octopus bimaculoides]
TRINITY_DN364182_c0_g1_i12	1.94	0.75	0.960	0.018	XP_015753655.1 PREDICTED: heat shock cognate 71 kDa protein-like [Acropora digitifera]
TRINITY_DN374497_c0_g1_i11	4.83	0.77	0.748	0.126	CAQ14843.1 novel protein similar to vertebrate heat shock cognate 70-kd protein (hsp70) [Danio rerio]
TRINITY_DN380425_c3_g1_i1	54.45	0.82	0.222	0.654	XP_017479226.1 PREDICTED: heat shock 70 kDa protein cognate 1 [Rhagoletis zephyria]
TRINITY_DN375872_c1_g1_i1	3.18	0.90	0.698	0.156	XP_022528366.1 heat shock 70 kDa protein [Astyanax mexicanus]
TRINITY_DN351755_c1_g3_i3	7.58	0.91	0.480	0.319	XP_009460485.1 PREDICTED: heat shock-related 70 kDa protein 2 [Nipponia nippon]
TRINITY_DN379150_c2_g5_i1	6.64	0.92	0.549	0.261	XP_018336683.1 PREDICTED: heat shock protein 70 A1-like [Agrilus planipennis]

Supplementary Table 1. Differential expression of contigs annotated as *hsp/hsc70*. *E. verrucosus*, 24 h heat shock / control

TRINITY_DN374946_c4_g1_i1	197.30	1.01	0.038	1.415	AFI60316.1 heat shock protein 70 [Eulimnogammarus verrucosus]
TRINITY_DN360138_c2_g1_i2	18.86	1.02	0.568	0.246	XP_017492203.1 PREDICTED: heat shock 70 kDa protein-like, partial [Rhagoletis zephyria]
TRINITY_DN355554_c0_g1_i3	251.13	1.02	0.626	0.203	XP_018012862.1 PREDICTED: heat shock 70 kDa protein cognate 5-like [Hyalella azteca]
TRINITY_DN362493_c1_g3_i1	10.95	1.13	0.312	0.505	XP_004522113.1 heat shock 70 kDa protein cognate 1 [Ceratitis capitata]
TRINITY_DN367665_c0_g2_i1	12.93	1.15	0.603	0.219	AFP54307.1 heat shock protein 70 cognate [Lycorma delicatula]
TRINITY_DN356069_c0_g2_i5	34.26	1.20	0.108	0.968	XP_012146197.1 PREDICTED: heat shock cognate 71 kDa protein-like isoform X2 [Megachile rotundata]
TRINITY_DN373011_c1_g2_i4	4.30	1.27	0.851	0.070	KMQ97439.1 heat shock 70 kda protein cognate 4-like protein [Lasius niger]
TRINITY_DN371955_c0_g1_i4	25.83	1.29	0.584	0.234	AAF87583.1 heat shock 70 protein [Parastrongyloides trichosuri]
TRINITY_DN350520_c4_g3_i5	2.46	1.33	0.741	0.130	XP_007435687.1 PREDICTED: heat shock-related 70 kDa protein 2 [Python bivittatus]
TRINITY_DN363376_c1_g2_i4	59.56	1.39	0.630	0.201	AKC91104.1 heat shock protein 70 [Stylophora pistillata]
TRINITY_DN350520_c4_g2_i1	8.37	1.43	0.371	0.431	XP_007435687.1 PREDICTED: heat shock-related 70 kDa protein 2 [Python bivittatus]
TRINITY_DN380671_c0_g1_i3	6.11	1.49	0.833	0.079	BAO23807.1 heat shock 70 kDa protein 2 [Brachionus plicatilis]
TRINITY_DN356069_c0_g2_i4	33.03	1.51	0.785	0.105	XP_022662585.1 heat shock cognate 71 kDa protein-like [Varroa destructor]
TRINITY_DN376172_c0_g1_i5	16.46	1.56	0.118	0.928	ASS36960.1 heat shock protein 70 [Mugilogobius chulae]
TRINITY_DN374556_c0_g1_i2	4501.32	1.57	0.000	5.947	XP_018017702.1 PREDICTED: LOW QUALITY PROTEIN: heat shock 70 kDa protein cognate 3-like [Hyalella azteca]
TRINITY_DN354305_c2_g1_i2	7.27	1.65	0.270	0.568	XP_022662585.1 heat shock cognate 71 kDa protein-like [Varroa destructor]
TRINITY_DN356069_c0_g2_i3	40.38	1.81	0.690	0.161	XP_022662585.1 heat shock cognate 71 kDa protein-like [Varroa destructor]
TRINITY_DN359242_c3_g3_i2	16.63	1.97	0.235	0.628	ADR00357.2 heat shock 70 kDa cognate protein [Ostrinia furnacalis]
TRINITY_DN376172_c0_g1_i6	5.37	2.07	0.522	0.283	XP_013096370.1 PREDICTED: heat shock protein 70 A1-like [Biomphalaria glabrata]
TRINITY_DN363376_c1_g2_i2	20.20	2.10	0.811	0.091	AKC91104.1 heat shock protein 70 [Stylophora pistillata]
TRINITY_DN360827_c0_g1_i9	1313.52	2.39	0.000	10.236	AEX65804.1 heat shock protein 70 [Gammarus lacustris]
TRINITY_DN363376_c1_g4_i1	2.12	2.40	0.554	0.256	AAF12746.1 heat shock protein 70 [Stylophora pistillata]
TRINITY_DN360827_c0_g4_i2	3.61	3.09	0.149	0.827	AFI60316.1 heat shock protein 70 [Eulimnogammarus verrucosus]
TRINITY_DN350520_c4_g3_i1	4.88	3.13	0.076	1.119	XP_007435687.1 PREDICTED: heat shock-related 70 kDa protein 2 [Python bivittatus]
TRINITY_DN361130_c0_g1_i1	791.50	3.18	0.000	6.907	AFI60316.1 heat shock protein 70 [Eulimnogammarus verrucosus]
TRINITY_DN363376_c1_g2_i1	35.64	3.23	0.242	0.615	AKC91104.1 heat shock protein 70 [Stylophora pistillata]
TRINITY_DN360827_c0_g1_i5	1578.51	3.47	0.000	14.372	XP_018015331.1 PREDICTED: heat shock cognate 71 kDa protein [Hyalella azteca]
TRINITY_DN361130_c0_g2_i1	99.78	3.54	0.000	4.330	XP_018416155.1 PREDICTED: heat shock 70 kDa protein isoform X1 [Nanorana parkeri]
TRINITY_DN378567_c1_g4_i1	477.32	3.76	0.000	12.031	AFI60316.1 heat shock protein 70 [Eulimnogammarus verrucosus]
TRINITY_DN352603_c0_g5_i1	9.14	4.05	0.003	2.577	AFI60316.1 heat shock protein 70 [Eulimnogammarus verrucosus]
TRINITY_DN360827_c0_g1_i6	178.90	4.54	0.000	3.746	AFI60316.1 heat shock protein 70 [Eulimnogammarus verrucosus]
TRINITY_DN377992_c2_g1_i2	54.80	4.62	0.000	10.085	AMQ67154.1 heat shock protein 70b, partial [Oncorhynchus mykiss]
TRINITY_DN370191_c0_g1_i10	2.58	4.80	0.137	0.863	XP_018017811.1 PREDICTED: heat shock 70 kDa protein II-like [Hyalella azteca]
TRINITY_DN345851_c0_g1_i1	4.09	5.37	0.116	0.937	CAQ60114.1 70kDa heat shock protein [Gammarus locusta]
TRINITY_DN360827_c0_g1_i7	635.20	5.51	0.000	3.685	AEX65804.1 heat shock protein 70 [Gammarus lacustris]
TRINITY_DN377992_c2_g1_i1	5.14	5.74	0.119	0.925	AMQ67154.1 heat shock protein 70b, partial [Oncorhynchus mykiss]
TRINITY_DN352603_c0_g1_i1	14.70	6.26	0.000	3.684	XP_018015328.1 PREDICTED: heat shock 70 kDa protein-like [Hyalella azteca]
TRINITY_DN378567_c0_g1_i1	12.45	6.76	0.000	3.596	BAD99026.1 heat shock protein 70 [Mytilus galloprovincialis]
TRINITY_DN360827_c0_g1_i3	42.04	6.92	0.001	3.004	AFI60316.1 heat shock protein 70 [Eulimnogammarus verrucosus]
TRINITY_DN378567_c0_g1_i3	77.98	8.70	0.000	4.326	XP_002733703.1 PREDICTED: heat shock cognate 71 kDa protein-like [Saccoglossus kowalevskii]
TRINITY_DN371471_c1_g2_i1	176.36	8.96	0.000	11.127	AHX41277.1 heat shock protein 70, partial [Gammarus lacustris]
TRINITY_DN348745_c2_g4_i1	249.01	9.35	0.000	14.174	XP_015342943.1 PREDICTED: heat shock 70 kDa protein 1-like [Marmota marmota marmota]
TRINITY_DN371471_c1_g1_i1	6425.13	9.45	0.000	127.724	CAQ60114.1 70kDa heat shock protein [Gammarus locusta]
TRINITY_DN377992_c3_g3_i2	157.12	9.70	0.000	9.583	CAQ60114.1 70kDa heat shock protein [Gammarus locusta]
TRINITY_DN378567_c0_g2_i1	969.34	9.89	0.000	28.690	CAQ60114.1 70kDa heat shock protein [Gammarus locusta]
TRINITY_DN354305_c1_g1_i1	1609.57	10.04	0.000	49.411	XP_018015327.1 PREDICTED: heat shock cognate 71 kDa protein-like [Hyalella azteca]
TRINITY_DN361336_c5_g1_i1	769.35	10.08	0.000	33.399	AFI60314.1 heat shock protein 70, partial [Eulimnogammarus marituji]
TRINITY_DN371471_c1_g2_i2	626.99	10.23	0.000	21.068	AHX41264.1 heat shock protein 70, partial [Gammarus lacustris]
TRINITY_DN377992_c3_g3_i4	6219.08	10.38	0.000	106.661	CAQ60114.1 70kDa heat shock protein [Gammarus locusta]
TRINITY_DN349252_c4_g6_i1	378.49	10.87	0.000	7.505	XP_011619500.1 PREDICTED: heat shock 70 kDa protein-like, partial [Takifugu rubripes]

Supplementary Table 1. Differential expression of contigs annotated as *hsp/hsc70*. *E. cyaneus*, 24 h heat shock / control

Contig name	baseMean	log2FoldChange	padj	log10padj	Best nr hit
TRINITY_DN509878_c5_g1_i1	5.60	-2.26	0.687	0.163	XP_012712026.1 heat shock cognate 71 kDa protein-like [Fundulus heteroclitus]
TRINITY_DN474476_c1_g3_i3	2.76	-1.56	0.571	0.243	ACI25099.1 heat shock protein 70 [Oreochromis niloticus]
TRINITY_DN506093_c7_g5_i2	5.01	-1.48	0.569	0.245	AGK45629.1 heat shock protein 70 [Brachionus calyciflorus]
TRINITY_DN477288_c0_g1_i1	4.52	-1.47	0.555	0.256	AAN78300.1 heat shock protein 70 A [Heterodera glycines]
TRINITY_DN482557_c0_g1_i6	7.03	-1.47	0.862	0.065	XP_003789082.1 PREDICTED: heat shock 70 kDa protein 1 [Otolemur garnettii]
TRINITY_DN503511_c0_g1_i1	2.91	-1.40	0.709	0.149	XP_018012862.1 PREDICTED: heat shock 70 kDa protein cognate 5-like [Hyalella azteca]
TRINITY_DN478567_c0_g1_i5	5.79	-1.32	0.512	0.291	KPM07870.1 Sar s 28 (heat shock protein 70-like protein 4), partial [Sarcoptes scabiei]
TRINITY_DN510091_c1_g1_i2	9.09	-1.30	0.453	0.344	XP_022643827.1 heat shock-related 70 kDa protein 2-like [Varroa destructor]
TRINITY_DN479809_c0_g3_i2	8.18	-1.19	0.430	0.366	ARK20000.1 heat shock 70 kDa protein cognate 4 [Ampulex compressa]
TRINITY_DN483662_c2_g3_i1	4.09	-1.17	0.873	0.059	BAO23807.1 heat shock 70 kDa protein 2 [Brachionus plicatilis]
TRINITY_DN510091_c1_g1_i6	47.51	-1.04	0.517	0.286	XP_022643827.1 heat shock-related 70 kDa protein 2-like [Varroa destructor]
TRINITY_DN510132_c2_g1_i1	28.82	-0.95	0.213	0.671	ACY69994.1 heat shock protein 70 [Pelophylax lessonae]
TRINITY_DN471750_c1_g7_i1	5.77	-0.94	0.677	0.169	XP_019762664.1 PREDICTED: heat shock 70 kDa protein cognate 4-like [Dendroctonus ponderosae]
TRINITY_DN485185_c6_g3_i2	3.58	-0.93	0.907	0.042	ABX89903.1 inducible heat shock protein 70 [Tigriopus japonicus]
TRINITY_DN497572_c2_g1_i2	6.25	-0.80	0.976	0.010	XP_011153497.1 PREDICTED: heat shock 70 kDa protein cognate 4 [Harpegnathos saltator]
TRINITY_DN505627_c0_g1_i4	34.04	-0.77	0.762	0.118	XP_015788504.1 PREDICTED: heat shock cognate 71 kDa protein-like [Tetranychus urticae]
TRINITY_DN510132_c2_g1_i3	11.81	-0.73	0.659	0.181	XP_020468877.1 heat shock 70 kDa protein 1 [Monopterus albus]
TRINITY_DN492220_c3_g3_i2	6.00	-0.69	0.956	0.019	XP_017479226.1 PREDICTED: heat shock 70 kDa protein cognate 1 [Rhagoletis zephyria]
TRINITY_DN505627_c0_g1_i2	4.72	-0.66	0.916	0.038	XP_015366535.1 PREDICTED: heat shock 70 kDa protein cognate 4-like [Diuraphis noxia]
TRINITY_DN492870_c2_g5_i1	2.85	-0.65	0.960	0.018	AGK45629.1 heat shock protein 70 [Brachionus calyciflorus]
TRINITY_DN510091_c1_g1_i1	31.36	-0.65	0.938	0.028	XP_022706516.1 heat shock-related 70 kDa protein 2-like [Varroa jacobsoni]
TRINITY_DN491353_c1_g1_i2	113.95	-0.61	0.329	0.482	XP_018019096.1 PREDICTED: heat shock 70 kDa protein 14-like, partial [Hyalella azteca]
TRINITY_DN471750_c0_g1_i3	2.20	-0.59	0.948	0.023	XP_022644917.1 heat shock 70 kDa protein 1-like [Varroa destructor]
TRINITY_DN511027_c1_g3_i2	235.26	-0.58	0.233	0.633	ABX89903.1 inducible heat shock protein 70 [Tigriopus japonicus]
TRINITY_DN481896_c0_g1_i1	2.87	-0.55	0.945	0.025	XP_015788504.1 PREDICTED: heat shock cognate 71 kDa protein-like [Tetranychus urticae]
TRINITY_DN460395_c0_g1_i1	6.71	-0.54	0.898	0.047	XP_019562417.1 PREDICTED: heat shock 70 kDa protein cognate 4-like [Aedes albopictus]
TRINITY_DN495692_c0_g2_i3	12.81	-0.51	0.940	0.027	XP_014237218.1 PREDICTED: heat shock 70 kDa protein cognate 4-like [Trichogramma pretiosum]
TRINITY_DN507307_c0_g1_i7	3.32	-0.48	0.966	0.015	XP_014237218.1 PREDICTED: heat shock 70 kDa protein cognate 4-like [Trichogramma pretiosum]
TRINITY_DN510278_c0_g1_i1	3.06	-0.38	0.969	0.014	XP_015269980.1 PREDICTED: LOW QUALITY PROTEIN: heat shock 70 kDa protein 1-like, partial [Gekko japonicus]
TRINITY_DN500648_c5_g1_i3	39.44	-0.35	0.842	0.075	XP_022528366.1 heat shock 70 kDa protein [Astyanax mexicanus]
TRINITY_DN489626_c2_g1_i1	6.25	-0.30	0.991	0.004	XP_022055649.1 heat shock 70 kDa protein 1 [Acanthochromis polyacanthus]
TRINITY_DN486605_c6_g1_i1	3.57	-0.25	0.983	0.008	XP_020444855.1 heat shock 70 kDa protein 1-like [Monopterus albus]
TRINITY_DN477288_c0_g4_i3	4.23	-0.19	0.985	0.007	ACU00685.1 heat shock protein 70 [Bursaphelenchus mucronatus]
TRINITY_DN511031_c0_g1_i3	7.60	-0.16	0.987	0.006	XP_007883910.1 PREDICTED: heat shock-related 70 kDa protein 2-like [Callorhinchus milii]
TRINITY_DN505003_c0_g2_i1	84.60	-0.15	0.966	0.015	XP_020468877.1 heat shock 70 kDa protein 1 [Monopterus albus]
TRINITY_DN478567_c0_g1_i3	6.81	-0.11	0.992	0.004	KPM07870.1 Sar s 28 (heat shock protein 70-like protein 4), partial [Sarcoptes scabiei]
TRINITY_DN505152_c1_g2_i2	50.56	-0.09	0.986	0.006	KPM07870.1 Sar s 28 (heat shock protein 70-like protein 4), partial [Sarcoptes scabiei]
TRINITY_DN511027_c1_g3_i1	29.40	-0.07	0.988	0.005	ABX89903.1 inducible heat shock protein 70 [Tigriopus japonicus]
TRINITY_DN479809_c0_g3_i1	10.54	-0.01	0.999	0.000	ARK20000.1 heat shock 70 kDa protein cognate 4 [Ampulex compressa]
TRINITY_DN482557_c0_g1_i7	12.07	0.03	0.999	0.001	XP_003789082.1 PREDICTED: heat shock 70 kDa protein 1 [Otolemur garnettii]
TRINITY_DN482557_c0_g1_i3	14.59	0.03	0.998	0.001	XP_003789082.1 PREDICTED: heat shock 70 kDa protein 1 [Otolemur garnettii]
TRINITY_DN485185_c6_g3_i1	7.31	0.03	0.998	0.001	ABX89903.1 inducible heat shock protein 70 [Tigriopus japonicus]
TRINITY_DN510091_c1_g1_i3	6.58	0.05	0.998	0.001	XP_022643827.1 heat shock-related 70 kDa protein 2-like [Varroa destructor]
TRINITY_DN497572_c2_g1_i1	17.68	0.06	0.996	0.002	XP_011153497.1 PREDICTED: heat shock 70 kDa protein cognate 4 [Harpegnathos saltator]
TRINITY_DN497572_c2_g1_i3	5.86	0.08	0.997	0.001	XP_011153497.1 PREDICTED: heat shock 70 kDa protein cognate 4 [Harpegnathos saltator]
TRINITY_DN506404_c0_g1_i1	43.66	0.10	0.991	0.004	BAO23807.1 heat shock 70 kDa protein 2 [Brachionus plicatilis]
TRINITY_DN499714_c0_g1_i1	31.01	0.18	0.961	0.017	XP_018015331.1 PREDICTED: heat shock cognate 71 kDa protein [Hyalella azteca]
TRINITY_DN478248_c8_g2_i1	28.19	0.18	0.979	0.009	XP_016974239.1 PREDICTED: heat shock 70 kDa protein cognate 1 [Drosophila rhopaloa]
TRINITY_DN507307_c0_g1_i6	27.08	0.24	0.968	0.014	XP_014237218.1 PREDICTED: heat shock 70 kDa protein cognate 4-like [Trichogramma pretiosum]
TRINITY_DN503511_c0_g2_i1	879.66	0.24	0.895	0.048	XP_018012862.1 PREDICTED: heat shock 70 kDa protein cognate 5-like [Hyalella azteca]
TRINITY_DN508803_c1_g2_i1	4.45	0.34	0.968	0.014	AGQ45967.1 heat shock protein 70 kDa [Diamesa cinerella]
TRINITY_DN490611_c1_g1_i2	34.97	0.35	0.884	0.054	XP_015371967.1 PREDICTED: heat shock protein 70 B2-like [Diuraphis noxia]
TRINITY_DN508779_c1_g1_i7	3.72	0.39	0.957	0.019	XP_014237218.1 PREDICTED: heat shock 70 kDa protein cognate 4-like [Trichogramma pretiosum]
TRINITY_DN481600_c0_g2_i1	10.05	0.50	0.927	0.033	AGH32327.1 heat shock protein 70 [Cellana toreuma]

Supplementary Table 1. Differential expression of contigs annotated as *hsp/hsc70*. *E. cyaneus*, 24 h heat shock / control

TRINITY_DN506939_c1_g6_i1	2241.76	0.50	0.340	0.468	AFI60316.1 heat shock protein 70 [Eulimnogammarus verrucosus]
TRINITY_DN506939_c1_g1_i3	5821.98	0.51	0.168	0.775	AFI60316.1 heat shock protein 70 [Eulimnogammarus verrucosus]
TRINITY_DN492220_c3_g3_i1	63.04	0.55	0.426	0.371	XP_021963158.1 heat shock 70 kDa protein cognate 4 [Folsomia candida]
TRINITY_DN481757_c2_g3_i1	79.72	0.56	0.377	0.424	AFI60316.1 heat shock protein 70 [Eulimnogammarus verrucosus]
TRINITY_DN489626_c2_g1_i4	17.78	0.59	0.950	0.022	XP_022055649.1 heat shock 70 kDa protein 1 [Acanthochromis polyacanthus]
TRINITY_DN507602_c0_g3_i1	8.39	0.63	0.833	0.079	AFX84560.1 70 kDa heat shock protein [Lygus hesperus]
TRINITY_DN487463_c1_g1_i1	16.50	0.66	0.772	0.112	XP_014289183.1 PREDICTED: major heat shock 70 kDa protein Ba-like [Halyomorpha halys]
TRINITY_DN491223_c3_g2_i3	744.25	0.67	0.132	0.880	XP_002754570.3 PREDICTED: heat shock cognate 71 kDa protein isoform X1 [Callithrix jacchus]
TRINITY_DN472333_c1_g1_i1	6.39	0.72	0.826	0.083	XP_018017702.1 PREDICTED: LOW QUALITY PROTEIN: heat shock 70 kDa protein cognate 3-like [Hyalella azteca]
TRINITY_DN496394_c0_g1_i2	15.85	0.75	0.576	0.239	NP_001298215.1 heat shock protein 70 B2-like [Biomphalaria glabrata]
TRINITY_DN497931_c0_g1_i8	10.11	0.83	0.753	0.123	XP_004479764.1 PREDICTED: heat shock 70 kDa protein 6 [Dasypus novemcinctus]
TRINITY_DN477288_c0_g2_i1	5.35	0.86	0.871	0.060	ASB34118.1 heat shock protein 70 [Eurytemora pacifica]
TRINITY_DN510573_c5_g1_i9	2.44	0.89	0.978	0.009	XP_007064665.1 PREDICTED: heat shock 70 kDa protein 1-like [Chelonia mydas]
TRINITY_DN489446_c0_g1_i3	4.07	0.89	0.915	0.039	XP_008052478.1 heat shock 70 kDa protein 6 [Carlito syrichta]
TRINITY_DN500039_c1_g2_i1	2.28	0.98	0.918	0.037	GAA37571.1 heat shock 70kDa protein 1/8 [Clonorchis sinensis]
TRINITY_DN506939_c1_g1_i1	36.13	1.08	0.286	0.544	XP_018011061.1 PREDICTED: heat shock 70 kDa protein cognate 4-like [Hyalella azteca]
TRINITY_DN489111_c0_g4_i1	89.27	1.10	0.008	2.089	XP_014237218.1 PREDICTED: heat shock 70 kDa protein cognate 4-like [Trichogramma pretiosum]
TRINITY_DN503324_c4_g1_i6	2934.08	1.13	0.222	0.654	XP_018017702.1 PREDICTED: LOW QUALITY PROTEIN: heat shock 70 kDa protein cognate 3-like [Hyalella azteca]
TRINITY_DN506093_c8_g1_i1	15.65	1.28	0.285	0.546	AFI60316.1 heat shock protein 70 [Eulimnogammarus verrucosus]
TRINITY_DN471750_c1_g2_i1	8.51	1.31	0.236	0.627	XP_004624350.1 PREDICTED: heat shock 70 kDa protein 1 isoform X1 [Octodon degus]
TRINITY_DN481757_c2_g2_i1	11.65	1.33	0.429	0.368	XP_018015331.1 PREDICTED: heat shock cognate 71 kDa protein [Hyalella azteca]
TRINITY_DN511031_c0_g1_i1	2.83	1.55	0.838	0.077	XP_007883910.1 PREDICTED: heat shock-related 70 kDa protein 2-like [Callorhinchus milii]
TRINITY_DN509878_c5_g1_i9	9.72	1.59	0.916	0.038	XP_012712026.1 heat shock cognate 71 kDa protein-like [Fundulus heteroclitus]
TRINITY_DN509878_c5_g1_i4	2.62	1.71	0.591	0.228	XP_012712026.1 heat shock cognate 71 kDa protein-like [Fundulus heteroclitus]
TRINITY_DN506939_c1_g1_i2	12.11	1.72	0.036	1.446	AFI60316.1 heat shock protein 70 [Eulimnogammarus verrucosus]
TRINITY_DN501569_c7_g1_i1	2.26	1.84	0.477	0.321	XP_007102348.1 PREDICTED: heat shock-related 70 kDa protein 2 [Physeter catodon]
TRINITY_DN491353_c1_g1_i1	6.29	1.92	0.921	0.036	XP_018019096.1 PREDICTED: heat shock 70 kDa protein 14-like, partial [Hyalella azteca]
TRINITY_DN464290_c0_g1_i1	76.08	2.58	0.000	3.800	AFI60316.1 heat shock protein 70 [Eulimnogammarus verrucosus]
TRINITY_DN506646_c2_g1_i1	10.81	2.78	0.023	1.642	ADJ96610.1 heat shock protein 70-p4 [Oxycera pardalina]
TRINITY_DN475692_c3_g1_i3	2.17	3.07	0.763	0.118	ABU89874.1 heat shock protein 70, partial [Ecteinascidia turbinata]
TRINITY_DN464290_c0_g1_i2	120.44	3.18	0.000	4.716	AFI60316.1 heat shock protein 70 [Eulimnogammarus verrucosus]
TRINITY_DN480896_c0_g3_i1	24.43	3.18	0.009	2.058	XP_005102694.1 PREDICTED: heat shock 70 kDa protein 4-like [Aplysia californica]
TRINITY_DN501569_c6_g1_i1	2.15	3.46	0.221	0.656	XP_013072146.1 PREDICTED: LOW QUALITY PROTEIN: heat shock 70 kDa protein-like [Biomphalaria glabrata]
TRINITY_DN491223_c3_g2_i2	323.26	3.68	0.000	8.546	AFI60316.1 heat shock protein 70 [Eulimnogammarus verrucosus]
TRINITY_DN506939_c0_g1_i4	619.96	3.91	0.000	11.344	AEX65804.1 heat shock protein 70 [Gammarus lacustris]
TRINITY_DN506939_c0_g1_i1	633.32	3.94	0.000	9.500	AEX65804.1 heat shock protein 70 [Gammarus lacustris]
TRINITY_DN501606_c2_g1_i1	40.96	4.66	0.000	8.999	XP_013790449.1 heat shock 70 kDa protein cognate 4-like [Limulus polyphemus]
TRINITY_DN501286_c2_g2_i1	40.13	5.30	0.000	5.264	XP_015797521.1 PREDICTED: heat shock 70 kDa protein 1 [Nothobranchius furzeri]
TRINITY_DN494491_c1_g1_i3	8.75	5.48	0.005	2.264	BAO23807.1 heat shock 70 kDa protein 2 [Brachionus plicatilis]
TRINITY_DN482083_c1_g3_i2	6.27	6.08	0.007	2.139	CAQ60114.1 70kDa heat shock protein [Gammarus locusta]
TRINITY_DN482083_c0_g2_i1	38.07	6.20	0.000	9.500	CAQ60114.1 70kDa heat shock protein [Gammarus locusta]
TRINITY_DN506646_c4_g1_i1	7.88	6.45	0.082	1.086	XP_006896234.1 PREDICTED: heat shock 70 kDa protein 1-like [Elephantulus edwardii]
TRINITY_DN503324_c4_g1_i3	10.72	6.92	0.002	2.793	XP_018017702.1 PREDICTED: LOW QUALITY PROTEIN: heat shock 70 kDa protein cognate 3-like [Hyalella azteca]
TRINITY_DN407492_c0_g1_i1	11.05	6.93	0.000	4.750	AHX41289.1 heat shock protein 70, partial [Gammarus decorosus]
TRINITY_DN510477_c2_g2_i2	15.93	7.45	0.000	4.318	AFI60316.1 heat shock protein 70 [Eulimnogammarus verrucosus]
TRINITY_DN510477_c2_g1_i1	493.64	8.18	0.000	52.595	CAQ60114.1 70kDa heat shock protein [Gammarus locusta]
TRINITY_DN510477_c2_g3_i1	1311.90	8.32	0.000	90.525	CAQ60114.1 70kDa heat shock protein [Gammarus locusta]
TRINITY_DN510477_c2_g3_i2	723.91	8.42	0.000	28.068	CAQ60114.1 70kDa heat shock protein [Gammarus locusta]
TRINITY_DN475381_c10_g1_i1	587.07	8.52	0.000	33.822	CAQ60114.1 70kDa heat shock protein [Gammarus locusta]
TRINITY_DN510477_c2_g2_i4	6655.88	8.67	0.000	56.782	CAQ60114.1 70kDa heat shock protein [Gammarus locusta]
TRINITY_DN510477_c2_g2_i3	57.91	8.97	0.000	7.646	CAQ60114.1 70kDa heat shock protein [Gammarus locusta]
TRINITY_DN510477_c2_g2_i1	115.41	9.42	0.000	14.017	XP_018015326.1 PREDICTED: heat shock cognate 71 kDa protein-like [Hyalella azteca]
TRINITY_DN482083_c1_g3_i1	100.96	9.86	0.000	15.249	CAQ60114.1 70kDa heat shock protein [Gammarus locusta]
TRINITY_DN482083_c0_g2_i2	128.92	10.13	0.000	14.484	CAQ60114.1 70kDa heat shock protein [Gammarus locusta]

Supplementary Table 2. Characteristics of the most abundant *hsp/hsc70* transcripts in the transcriptome assemblies.

Assembly	Contig name	qstart	qend	sstart	send	evalue	Best nr hit	overexpressed	log2FC	padj
E. verrucosus (EveBCdTP1)	TRINITY_DN347332_c2_g1_i1	2	406	390	524	5,81E-83	AFI60316.1 heat shock protein 70 [Eulimnogammarus verrucosus]	FALSE	0,6	2,97E-01
	TRINITY_DN349252_c4_g6_i1	326	3	401	508	2,01E-70	XP_011619500.1 PREDICTED: heat shock 70 kDa protein-like, partial [Takifugu rubripes]	TRUE	10,9	3,13E-08
	TRINITY_DN361130_c0_g1_i1	1	339	499	611	3,67E-70	AFI60316.1 heat shock protein 70 [Eulimnogammarus verrucosus]	TRUE	3,2	1,24E-07
	TRINITY_DN361336_c5_g1_i1	346	2	87	201	4,85E-63	AFI60314.1 heat shock protein 70, partial [Eulimnogammarus marituji]	TRUE	10,1	3,99E-34
	TRINITY_DN371471_c1_g1_i1	1	894	217	514	0	CAQ60114.1 70kDa heat shock protein [Gammarus locusta]	TRUE	9,5	1,89E-128
	TRINITY_DN371471_c1_g2_i2	2	301	132	231	6,26E-60	AHX41264.1 heat shock protein 70, partial [Gammarus lacustris]	TRUE	10,2	8,56E-22
	TRINITY_DN374556_c0_g1_i2	3	554	429	611	9,98E-70	XP_018017702.1 PREDICTED: LOW QUALITY PROTEIN: heat shock 70 kDa protein cognate 3-like [Hyalella azteca]	TRUE	1,6	1,13E-06
	TRINITY_DN377992_c3_g3_i2	3	53	595	611	8,24E-08	CAQ60114.1 70kDa heat shock protein [Gammarus locusta]	TRUE	9,7	2,61E-10
	TRINITY_DN377992_c3_g3_i4	3	146	564	611	1,77E-12	CAQ60114.1 70kDa heat shock protein [Gammarus locusta]	TRUE	10,4	2,18E-107
	TRINITY_DN378567_c0_g2_i1	1	369	48	170	7,21E-69	CAQ60114.1 70kDa heat shock protein [Gammarus locusta]	TRUE	9,9	2,04E-29
	TRINITY_DN378567_c1_g1_i2	1	324	492	599	2,66E-73	AFI60316.1 heat shock protein 70 [Eulimnogammarus verrucosus]	FALSE	0,1	9,34E-01
	TRINITY_DN378567_c1_g2_i2	3	1058	75	426	0	AFI60316.1 heat shock protein 70 [Eulimnogammarus verrucosus]	FALSE	0,2	7,62E-01
E. cyaneus (EcyBCdTP1)	TRINITY_DN475381_c10_g1_i1	356	3	279	396	4,38E-66	CAQ60114.1 70kDa heat shock protein [Gammarus locusta]	TRUE	8,5	1,51E-34
	TRINITY_DN491223_c3_g2_i3	73	258	1	62	1,5E-41	XP_002754570.3 PREDICTED: heat shock cognate 71 kDa protein isoform X1 [Callithrix jacchus]	FALSE	0,7	1,32E-01
	TRINITY_DN503324_c4_g1_i6	3	815	340	611	3,6E-105	XP_018017702.1 PREDICTED: LOW QUALITY PROTEIN: heat shock 70 kDa protein cognate 3-like [Hyalella azteca]	FALSE	1,1	2,22E-01
	TRINITY_DN506939_c1_g1_i3	1	636	400	611	1E-142	AFI60316.1 heat shock protein 70 [Eulimnogammarus verrucosus]	FALSE	0,5	1,68E-01
	TRINITY_DN506939_c1_g6_i1	1	780	85	344	0	AFI60316.1 heat shock protein 70 [Eulimnogammarus verrucosus]	FALSE	0,5	3,40E-01
	TRINITY_DN510477_c2_g1_i1	2	283	17	110	8,12E-57	CAQ60114.1 70kDa heat shock protein [Gammarus locusta]	TRUE	8,2	2,54E-53
	TRINITY_DN510477_c2_g2_i4	1	873	321	611	0	CAQ60114.1 70kDa heat shock protein [Gammarus locusta]	TRUE	8,7	1,65E-57
	TRINITY_DN510477_c2_g3_i1	7	564	139	324	1,7E-120	CAQ60114.1 70kDa heat shock protein [Gammarus locusta]	TRUE	8,3	2,99E-91
	TRINITY_DN510477_c2_g3_i2	1	660	105	324	1,7E-140	CAQ60114.1 70kDa heat shock protein [Gammarus locusta]	TRUE	8,4	8,55E-29

Supplementary Table 2. Characteristics of the most abundant *hsp/hsc70* transcripts in the transcriptome assemblies.

Assembly	Contig name	Amino acid motifs						
		TVPAYFND	NEPTAA	IFDLGGGTF DVSIL	RARFEEL (cytos.)	GPTIEEVD (cytos.)	ADAYLGTN	C-end GG*P
E. verrucosus (EveBCdTP1)	TRINITY_DN347332_c2_g1_i1							
	TRINITY_DN349252_c4_g6_i1							
	TRINITY_DN361130_c0_g1_i1					V		V (GGMP)
	TRINITY_DN361336_c5_g1_i1	V	V					
	TRINITY_DN371471_c1_g1_i1							
	TRINITY_DN371471_c1_g2_i2	V	V	V				
	TRINITY_DN374556_c0_g1_i2							
	TRINITY_DN377992_c3_g3_i2					V		
	TRINITY_DN377992_c3_g3_i4					V		
	TRINITY_DN378567_c0_g2_i1	V	V					
	TRINITY_DN378567_c1_g1_i2							
	TRINITY_DN378567_c1_g2_i2	V	V	V	V		V	
E. cyaneus (EcyBCdTP1)	TRINITY_DN475381_c10_g1_i1							
	TRINITY_DN491223_c3_g2_i3							
	TRINITY_DN503324_c4_g1_i6							
	TRINITY_DN506939_c1_g1_i3	V	V	V	V		V	
	TRINITY_DN506939_c1_g6_i1					V		
	TRINITY_DN510477_c2_g1_i1							
	TRINITY_DN510477_c2_g2_i4					V		
	TRINITY_DN510477_c2_g3_i1	V	V	V				
	TRINITY_DN510477_c2_g3_i2	V	V	V				

Supplementary Table 3. Mortality under heat shock.

Species	Temperature tested, °C	Experimental period	Number of animals tested	Number of dead animals	Percent mortality	Mean percent mortality (per animal)	Median percent mortality (by experiment)
E. verrucosus	24	September (2013)	10	1	10%	17%	10%
	24	September (2013)	10	1	10%		
	24	September (2013)	10	0	0%		
	25	September (2013)	10	1	10%		
	25	September (2013)	10	3	30%		
	25	September (2013)	10	3	30%		
	24.5	May (2018)	12	3	25%		
E. cyaneus	24	September (2013)	10	1	10%	15%	10%
	24	September (2013)	10	4	40%		
	24	September (2013)	10	2	20%		
	25	September (2013)	10	1	10%		
	25	September (2013)	10	0	0%		
	25	September (2013)	10	0	0%		
	25.5	June (2018)	14	3	21%		

Supplementary Table 4. Oligonucleotides used in this work.

Primer pair name	Forward primer sequence 5’ to 3’	Reverse primer sequence 5’ to 3’	Region	Reference
Ecyaneus_gapdh	ACTCTACTCACGGCGTCTTCAAG	CGCTGGACTCTACGATGTACTCAG	*gapdh* , the 5’ part of the gene	Protopopova *et al.* 2014
HSP70_Eve (Eve_hsp70_I)	CCAAGATGAAGGAGACTGCTGATG	CGCCGTGGGTTCGTTGATAATC	*hsp70* , thre region coding for the NBD (approx. amino acids 125-178)	Protopopova *et al.* 2014
HSP70_Ecy (Ecy_hsp70_I)	GCCATTGCCTACGGTCTTGAC	CTCCTGCGTGAAGTGGTTGAC	*hsp70* , the region coding for the NBD (approx. amino acids 125-178)	Protopopova *et al.* 2014
HSP70_EveEcy_tr_3 (hsp70_II)	GACACCCATCTTGGAGGAGA	GCGCTTGTTTTGTGTGATGT	*hsp70* , the region coding for the NBD (approx. amino acids 256-259)	This work
HSP70_EveEcy_tr_1 (hsp70_III)	GCTTTCTGGACTTCCACCAG	CGTTGGTGATGGTGATCTTG	*hsp70* , the region coding for the SBD (approx. amino acids 462-506)	This work

Supplementary Data 1. Nucleotide sequences of contigs in Supplementary Table 2.

>EveBCdTP1_TRINITY_DN347332_c2_g1_i1
AGATCTGCTGCTCCTTGATGTTGCACCTCTTTCCATGGGCATTGAAACTGCCGGAGGTGT
GATGACTGCTCTCATCAAGCGTAACACAACCATTCCTACCAAACAGACCCAGACCTTCAC
CACCTACTCTGACAACCAGCCTGGTGTGTTGATCCAGGTCTACGAGGGCGAGCGTGCCAT
GACCAAGGATAACAACCTCCTCGGGAAGTTCGAGCTCACCGGAATTCCCCCCGCGCCCCG
TGGCGTTCCTCAGATCGAGGTGACCTTCGACATCGATGCCAACGGTATCCTGAACGTGTC
TGCCGTGGACAAGTCGACAGGCAAGGAAAACAAGATCACCATTACCAACGACAAGGGTCG
TCTGAGCAAGGAGGAGATCGAGCGTATGGTCCAGGACGCCGAGAGGAACA
>EveBCdTP1_TRINITY_DN349252_c4_g6_i1
CGTCCTTTGTCGTTGGTGATGGTGATCTTGTTCTCTTTGCCAGTGGATTTGTCAACGGCT
GACACGTTCAAAATGCCGTTGGCGTCGATGTCGAAAGTGACTTCAATCTGCGGCACTCCC
CTTGGAGCTGGTGGAAGTCCAGAAAGCTCAAACTTACCGAGGAGATTGTTATCTCGGGTC
ATCGCGCGTTCTCCTTCAAACACCTGGATCAGAACACCAGGCTGATTGTCGGCGTACGTC
GAGAAAACCTGTGAAGTTTTCGTCGGGATTGTCGTGTTACGTTTGATCAGTGCAGTCATC
ACACCTCCAGCGGTTTCAATTCCCATG
>EveBCdTP1_TRINITY_DN361130_c0_g1_i1
AACAAGATCACAATCACCAACGACAAGGGTCGTCTGAGCAAGGAGGAGATCGAGCGCATG
GTAAAGGATGCAGAGAAGTACAAAAGCGACGATGACAACCAGCGCGAGCGCATCTCTGCC
AAGAACGGTCTTGAGTCCTACTGCTTCAACATGAAGTCTACTATTGAGGACGATAAGGTG
AAAGACAAGATCTCTGAGACCGACCGCAAGACTATCATGAATGCTTGCGACGAAGCAGTA
AAGTGGTTGGATGGTAACCAACTCGCCGAGATTGAAGAGTACAAGCATAAGCAGAAGGAA
ATCGAGAAGGTGTGCACGCCAATCATCACAAAGATGTACGGCGGTGCGGATGGCATGCCC
GGTGGCATGCATGGCGGTATGCCTGGTGGAGCGACAGCTGCAGCGGGTGGACCAACCATT
GAGGAAGTAGACTAACTTCGCAGAACTACTTTTCCCTAATTCGTTCAACTATCTTTCATC
TGTCTCATTGATTGAACTAAATAGTTTATTTAAACAAGGAAATTCGAGTCATTGTATGTA
AGTTTGGCGTTGTAGCTGACTTGGGGCTGCCTCTTTAACCGGTTCCTGTGTTGTATGCTA
CTTTAGATTTGTTTGTGTATTTACCAAAGGAAGCTTTTAAAAAGATTCATTTATATTTGT
AGCGTTAAATGTTCATGTTCTTTTTTCGCCATCTTTATAGGTTCGG
>EveBCdTP1_TRINITY_DN361336_c5_g1_i1
CGCCCAAGTCGAAGATCAGGATGTTTTTCTCGACACTCTCTATCTTGTCAAGGCCGTACG
CGATGGCGGCGGCTGTTGGTTCGTTGATGATCCGAAGCACGTTGAGACCAGCGATGGCAC
CGGCATCCTTGGTGGCTTGGCGCTGAGAGTCGTTGAAGTAGGCCGGCACCGTGATTACGG
CATCCTTCACTTCTTTACCTAGATAGGCCTCCGATATTTCCCTCATCTTTGTCAGGACCA
TGGAAGAGATCTCCTCTGGTGAGAAGGTCTTGCTCTCCCCCTTGAACTCCACGCCGATCA
TAGGCTTTCCTCCATCGTTGACCACATTAAAGGGCCAGTGTTCCATGCCCCG
>EveBCdTP1_TRINITY_DN371471_c1_g1_i1
TTTGAAGTCAAAGCTACAGCTGGTGACACCCATCTTGGAGGAGAGGACTTCGACAATCGC
TTGGTGGACCACTTCGCCCAAGAGTTCAAGAGGAAGTTCAAGAAGGACATCACACAAAAC
AAGCGCGCTCTAAGACGCCTGAGAACAGCGTGCGAGAGAGCCAAGAGAACACTGTCCTCG
TCCGCTCAAGCCAGCATTGAGATCGACTCCCTCTATGAAGGTACTGACTTCTATACGTCC
ATCACTCGAGCAAGGTTCGAAGAGATGTGCTCGGATCTTTTCAGGGGAACATTGGATCCT
GTGGAAAAATCCCTTCGGGACGCAAAGATGGACAAGGGAAATATCCACGAGATCGTACTG
GTTGGCGGATCAACTCGCATTCCCAAGATCCAGAAGCTGCTTCAAGATTTTTTCAATGGG
AAAGAGCTCAATAAAACCATCAACCCCGACGAGGCTGTGGCCTACGGGGCGGCCGTCCAG
GCGGCTATTCTGACTGGAGACAATTCAGAGGCTGTGCGAGACCTGCTGCTGTTGGATGTG
GCTCCCCTTTCCATGGGAATTGAAACCGCTGGAGGTGTGATGACCGCACTGATCAAACGT
AACACGACAATCCCGACGAAAACTTCACAGGTTTTCTCGACGTACGCCGACAATCAGCCT
GGTGTTCTGATCCAGGTGTTTGAAGGAGAACGCGCGATGACCCGAGATAACAATCTCCTC
GGTAAGTTTGAGCTTTCTGGACTTCCACCAGCTCCAAGGGGAGTGCCGCAGATTGAAGTC
ACTTTCGACATCGACGCCAACGGCATTTTGAACGTGTCAGCCGTTGACAAGTCCACTGGC
AAAGAGAACAAGATCACCATCACCAACGACAAAGGACGTCTTAGCAAAGAAGACAT
>EveBCdTP1_TRINITY_DN371471_c1_g2_i2
GGAGGCCTATCTAGGTAAAGAAGTGAAGGATGCCGTAATCACGGTGCCGGCCTACTTCAA
CGACTCTCAGCGCCAAGCCACCAAGGATGCCGGTGCCATCGCTGGTCTCAACGTGCTTCG
GATCATCAACGAACCAACAGCCGCCGCCATCGCATACGGCCTTGACAAGAAAGGGAAAGG
CGAAAAGAACATCCTGATCTTCGACTTGGGTGGAGGAACATTTGATGTATCTATTCTAAC
AATTGATGATGGCATCTTTGAAGTCAAAGCTACAGCTGGCGACACCCATCTTGGAGGAGA
GGA
>EveBCdTP1_TRINITY_DN374556_c0_g1_i2
TCTCGACGGCCTCTGATAACCAGCACACCGTCACCATCCAGGTGTTCGAGGGCGAGCGTC
CCATGACTAAGGACAACCACGTCCTTGGCAAGTTCGACCTTACCGGAATCCCACCTGCGC
CCCGTGGTGTGCCTCAGATTGAAGTGACCTTCGAAATCGACGCCAACGGCATCCTGCAAG
TGTCTGCTGAGGATAAGGGTACCGGCAACAAGGAGAAGATCACCATCACAAACGATCAGA
ACAGACTTACTCCGGAGGATATCGAAAGAATGATTAAGGATGCCGAAGTTTTCGCCGATG
AGGACAAGAAACTAAAGGAGAGGGTGGATGCTCGCAACGAGCTCGAGTCTTACTCGTACA
GTCTCAAGAACCAGATAAACGACAAGGAAAAGTTGGGGGCGAAGCTCTCTGATGAGGAGA
AAGAAAAGATGGAGGGCGCTATCGACGAATCTATCAAGTGGTTGGAAGATCATCCTGAAG

CTGAGGCCGAAGAGTACAAGAGCCAAAAGAAAGAATTGGAAGATATTGTCCAACCCATCA
TTGCTAAACTGTACCAAGGAGCACCACCTCCCGGTGGAGAAGGAGCTTCTGAAGGAGAGG
ACGAAGATGGCGGAGATTTCAAGGATGAATTGTAAAGCTTTTAGGCTTGTATAAATTATC
AACAAACTTGTAATGATAAGGTTAGCAATAGTGGCAAACTTCAAACGTTGTAAGGATTTT
TTTTGTTACTTGGGTGGAGAAAGCGTGTACATACTGACCTACTGTATGTGTGGTAGTACT
TTTACTGTGAACGAATAACTGAATAGTAGAATACTCCGTGTATGAATGTTTATTTATTTC
ATTTGTTAAAGGGTGAACGGAGAAATTTGCTCTAGTATATGAGACGGGGGGCATTCACGT
CATGTTTTACATCAGCGGAATTTCATCTCATTACTGTGATAGGCTCACTAGGCAATAACA
AGACTTTTTGAATGACTGATGTTCATAATAAATTGTTGAAAATTAAATTTACCAATTTCG
AATCCTTTCCAGAAGTAGAAAATTGCCTTGTCAACAAACTCTCGATTCCATCTATAAATT
AAACTGTTCGGTAGATTTAGAATTGAAATTCTGCACTGTACGAGGACACACAAAAGTTGA
GCTACTAGTATTGTTTGATCTAAACAAAATCTGATTAAATTTTGTGGATATACTCTGTGC
AAGCATTTATAGATCCCCTGAACTCAATTAGACTTGTTGAATCGTATGCATTTGTGCAAT
AAACTCTACTAGCC
>EveBCdTP1_TRINITY_DN377992_c3_g3_i2
ACAAGCAGAAGGAGGTAGAGAAGGTGTGCTCCCCAATCATCAGCAAGCTGTACGGAGGGG
GAGCTGCTGGCTGCTCGCCGGGGGCTGGGCCCATGCCGGGGGCAGCTAATGGGGCAGGAC
CCACCATAGAAGAAGTAGACTAAGCTTATAAAACTCATTAGTTAATACCATGTTCATTCA
GAGTCATTGTTATTAGCATTCCGTATTGTTACATTTGCCATACTAGTCTTTAT
>EveBCdTP1_TRINITY_DN377992_c3_g3_i4
GCGAGTCCGATCGCAGCAAAGTGTTGGAGGCTTGCAACGAAGTCCTCAAGTGGCTGGACG
CCAACCAGCTCGGCGAGAAGGACGAGTACGAGTACAAGCAGAAGGAGGTAGAGAAGGTGT
GCTCCCCAATCATCAGCAAGCTGTACGGAGGAGCTGCTGGCTGCTCGCCGGGGGCTGGGC
CCATGCCGGGGGCAGCTAATGGAGCAGGACCCACCATAGAAGAAGTAGATTAAAAGTTAG
GATATCGTTGTGATCATATTGATTCATTTTCTTCAAGCACGCTGTACCTTTCATTCCTAT
CAATGGTAGCATTGATGTTCTGTTTTGCGTTACATAAAAGTAACTAGTATACGTGCTATT
GCATTTGCCATCCTTCACAATATATTATATTTTGTAATAAATATTTTGTCATACATATGT
ACGTTTTAAATAATGTCTTTAATTATCGCTCACTTGTACATATTTGTTGTTATTTTATGT
CTTGTTAACCTTGGTAGAAGAATACAAATATATTTGATTTAAATGTTTTTATTTCCTTCA
AATTTTCGTGTGCACGACTTATTATTTTCGCTAACTTTTTTACTCCCTTTATCACATGGC
TTTATCACATGGCTTTATCACATGGCTTTATCACATGGCTTTATCAC
>EveBCdTP1_TRINITY_DN378567_c0_g2_i1
GAGAGGCTCATAGGAGACGCCGCCAAGAACCAGGTGGCTCTGAACCCCACCAACACCGTC
TTTGATGCCAAGAGGCTCATCGGACGCAAGTTCGGCGACTCTTCCGTCAACAAGGACATG
GAACACTGGCCCTTTAAGGTGGTCAACGATGGAGGAAAGCCTATGATCGGCGTGGAGTTC
AAGGGGGAGAGCAACACCTTCTCACCAGAGGAGATCTCTTCCATGGTCCTGACAAAGATG
AGGGAAATATCGGAGGCCTATCTAGGTAAAGAAGTGAAGGATGCCGTAATCACGGTGCCG
GCCTACTTCAACGACTCTCAGCGCCAAGCCACCAAGGATGCCGGTGCCATCGCTGGTCTC
AACGTGCTTC
>EveBCdTP1_TRINITY_DN378567_c1_g1_i2
GACAAGTCGACAGGCAAGGAAAACAAGATCACCATTACCAACGACAAGGGTCGTCTGAGC
AAGGAGGAGATCGAGCGTATGGTCCAGGACGCCGAGAAGTACAAGAATGACGACGACAAC
CAGCGTGAACGCATCTCTGCCAAGAACGGTCTCGAGTCTTATTGCTTCAACATGAAGTCT
ACCGTCGAGGACGATAAGGTCAAAGATAAGATCTCCGAGGATGACCGCAAGAAGATCATG
GAGGCTTGCGACGAAGCCATCAAGTGGTTGGATGGTAACCAGCTTGCCGAGAAGGAAGAG
TACGAGCACAAGCAGCAGAAGATCGGAAGGCACACCAGCTG
>EveBCdTP1_TRINITY_DN378567_c1_g2_i2
TCGGGCGCAAGTTCGACGAAGCCACTGTGCAGAGTGACATGAAGCACTGGCCCTTCGAGG
TGGTCAATGAGAACACCAAGCCCAAAATCAGTGTGGACTACAAAGGTGAGAAGAAGACCT
TCACCCCTGAAGAGATCTCCTCTATGGTCCTCACCAAGATGAAGGAGACTGCTGATGCTT
ACCTCGGCACTAACATCAAGGACGCTGTTGTCACAGTGCCAGCTTATTTCAACGATTCTC
AGCGTCAGGCAACAAAGGATGCGGGTACCATCTCTGGCCTCAACGTTTTGAGGATTATCA
ACGAACCCACGGCGGCCGCTATTGCCTACGGTCTTGACAAGAAGGTCGGAGGCGAGCGCA
ACGTCCTTATCTTCGATTTGGGCGGTGGTACTTTCGATGTGTCTATCCTGACTATTGAGG
ATGGCATCTTTGAGGTGAAGTCTACTGCTGGAGACACCCATCTTGGTGGTGAAGATTTCG
ACAATCGCATGGTCAACCACTTCATGCAAGAGTTCAAGCGCAAGTACAAGAAGGATCTCT
CTGAGAATAAGCGTTCACTGAGGAGGCTCAGAACTGCCTGCGAACGCGCAAAGCGTACCC
TTTCCTCTTCAACCCAGGCCAGTATCGAGATTGATTCTCTTTACGATGGTATCGACTTCT
ATACCTCGGTCACACGTGCTCGATTCGAAGAGCTGTGCGCCGATCTTTTCCGTGGTACCC
TCGACCCCGTCGAGAAGTCTCTCCGTGATGCTAAGCTCGACAAGGGCCAGATTCAGGAAA
TCGTACTTGTTGGAGGCTCCACTCGTATCCCCAAGATCCAGAAGCTCCTTCAGGACTTTT
TCAACGGCAAGGAACTAAATAAGTCCATCAACCCTGATGAGGCCGTCGCCTACGGTGCTG
CCGTTCAGGCTGCTATTCTTTCCGGAGACAAGTCTGAGGCTGTCCAGGACCTGCTGCTCC
TTGATGTTGCCCCTCTTTCCATGGGCATTGAAACTGCCGGCGGTGTGATGACTGCTCTCA
TCAAGCGTAACACAACCATTCCTACCAAACAGACCCAGAC
>EcyBCdTP1_TRINITY_DN475381_c10_g1_i1
GCCACATCCAACAGCAGCAGGTCTCGCACAGCCTCTGAATTGTCGCCTGTCAGAATAGCC
GCCTGGACGGCCGCCCCGTAGGCCACAGCCTCGTCGGGGTTGATGGATTTATTGAGCTCT
TTGCCATTGAAGAAATCTTGAAGCAGCTTCTGGATCTTGGGAATGCGAGTTGATCCGCCA
ACCAGTACGATCTCGTGGATCTGTCCCTTGTCCATCTTTGCGTCCCGAAGGGATTTTTCC
ACAGGTTCCAACGTTCCCCTGAAAAGATCCGAGCACATCTCTTCGAACCTTGCTCGAGTG

ATGGACGTATAGAAGTCAGTACCTTCATAGAGGGAGTCGATCTCAATGCTTGCTTGGGCG
>EcyBCdTP1_TRINITY_DN491223_c3_g2_i3
TTTGAGTGCTGATCGTGGGCACGAGTGCACGTTCTAAATTTTGAGGACCTAATTTACATT
CCGAACAACACCATGTCGAAGGCAACTGCTGTGGGTATCGATCTGGGCACCACGTACTCG
TGCGTGGGCGTCTTCCAGCATGGAAAGGTTGAGATCATTGCCAATGACCAGGGAAACAGG
ACCACGCCTTCCTACGTTGCCTTCACCGACACCGAGAGGCTCATCGGTGATGCTGCCAAG
AACCAGGTGGCCATGAAT
>EcyBCdTP1_TRINITY_DN503324_c4_g1_i6
GTTCCACTCGTATACCTAAGGTCCAGCAGCTCGTAAAGGAGTTCTTCAACGGCAAGGAGC
CATCCCGAGGCATCAACCCCGACGAGGCTGTGGCGTACGGTGCCGCCGTCCAGGCCGGTG
TTCTCTCTGGTGAAGACGATACCAGCGACTTGGTTCTCTTGGACGTGAACCCTCTGACCC
TCGGTATCGAGACAGTTGGAGGAGTGATGACCAAGCTCATTAACAGGAACACTGTCATTC
CCACCAAAAAGTCGCAGATCTTCTCGACGGCCTCTGATAACCAGCACACCGTCACCATCC
AGGTGTTTGAGGGCGAGCGTCCCATGACTAAAGACAACCACGTCCTTGGAAAGTTCGACC
TTACCGGAATCCCACCTGCGCCCCGTGGTGTGCCTCAGATTGAAGTGACCTTCGAAATCG
ACGCCAACGGCATCCTGCAAGTGTCTGCTGAGGATAAGGGTACCGGCAACAAGGAGAAGA
TCACAATCACAAACGATCAGAACAGACTTACTCCGGAGGATATCGAAAGAATGATTAAGG
ATGCCGAAGTTTTCGCCGATGAGGACAAGAAACTGAAGGAGAGGGTGGATGCTCGCAACG
AGCTCGAGTCTTACTCGTACAGTCTCAAGAACCAGATTAACGACAAGGAAAAGTTGGGGG
CGAAGCTCTCTGATGAGGAGAAAGAAAAGATGGAGGGCGCCATCGACGAATCTATCAAGT
GGTTGGAAGATCATCCTGAAGCAGAGGCCGAAGAGTACAAGAGCCAAAAGAAAGAATTGG
AAGATATTGTCCAGCCCATCATTGCTAAACTGTACCAAGGAGCACCACCTCCCGGTGGGG
AAGGAGCTTCTGAAGGCGAGGACGAAGATGGCGGAGATTTCAAGGATGAATTGTAAAGCT
TTTAGGCTTGTATAAATATCAACAAACTTGTAATGATAAGGTTAGCAATAGCGGCAAACT
TGGAACGTTGTAAGGATTTTTTTGTTACTTGGGTGGAGAAAGCGTGTACATACTGACCTA
CTGTATGTGTGGTAGTACTTTTACTGTGAACGAATAACTGAATAGTAGAATACTCCGTGT
ATGAATGTTTATTTATTTCATTTGTTAACGAGTGAACGGAGAAATTTGCTCTAGTATATG
AGACAGGGGGCATTCACGTCATGTTTTACATCAGCGGTCATTTCATCTCATTACTGTGAT
AGGCTCACTAGGCAATAACAAGACTTCTTCAATGACTGATGTTCATAATAAATTGTTGAA
AATTAAATTTACCAATTTCGAATCCTTTCCAGAAGTAGAAAATTTCCTTGTCAACAAACT
CTCGATTCCATCTATAAATTAAACTGTGTTCGGTAGATTTAGAATTGAAACTGCACTGTA
CGGGGACACACAAAAATTGAGCTACTAGTATTGTTTGATTTAAACAAAATCTGATTAAAT
TTTGTGGATATATTGTTCTGTGCAAGCATTTATAGATCCCCTGAACTGAATTAGACTTGT
TGAATAGTATGCATTTGTGCAATAAACTCTACTAACCGAACTATTCGACATGACAACACT
TCTGAAATCAATACCAGATGAACCACGTGAAGTCTGGTGTTGGGGAGAATGATTTTATAA
TTTGGCTTGGGATATTAATAGTGTTTATTTAGGCCAGGGGATATTTTTGACACTTCTGTA
CGATATCTACTCTTCATTAGATTATTGATCTGATTTACGGCTCTGCTCTCCAGTTCTCAC
TCTTGACGCTGTTGGCTAAACTTCACGGATTCAATGATCGGTCAAATTATCAAGTTGAGA
CTTCTCAAAATTTGCTCCCGGC
>EcyBCdTP1_TRINITY_DN506939_c1_g1_i3
TCCATGGGCATTGAAACTGCCGGCGGTGTAATGACTACTCTCATCAAGCGAAACACAACC
ATTCCTACCAAACAGACCCAGACCTTCACCACCTACTCTGACAACCAGCCTGGTGTGTTG
ATCCAGGTCTACGAGGGCGAGCGTGCCATGACCAAGGATAACAACCTCCTCGGGAAGTTC
GAGCTCACCGGAATTCCCCCCGCGCCCCGTGGCGTTCCTCAGATCGAGGTGACCTTCGAC
ATCGATGCCAACGGTATCCTGAACGTGTCTGCCGTGGACAAGTCGACAGGCAAGGAGAAC
AAGATCACCATTACCAACGACAAGGGTCGTCTGAGCAAGGAGGAGATTGAGCGTATGGTC
CAGGACGCCGAGAAGTACAAGAATGACGACGACAACCAGCGTGAGCGCATCTCTGCCAAG
AACGGTCTCGAGTCTTATTGCTTCAACATGAAGTCTACCGTCGAGGACGATAAGGTCAAG
GATAAGATCTCTGAGGATGACCGCAAGAAGATCATGGAGGCTTGCGACGAAGCCATCAAG
TGGTTGGATGGTAACCAGCTTGCCGAGAAGGAAGAGTATGAGCACAAGCAGAAAGAAGTC
GAGAAGGTGTGCACACCTATCATTACTAAACTGTATGGAGGTGCCGGAGCTGCTCCCCCT
CCCGGTGCCGCCCCTGGTACCGGTGCCGCCCCCGGAGCCGGAGGCGCTGGCGGGCCTACC
ATCGAGGAAGTAGACTAATCTATTCGTCCTTATTAGTCTTACCTTCCCCTTTCTGCGGTA
CTGATTGACGTTTGCTTATTGCCTTCTTCGATTGAACTGTCACGAAGTAAGAAATTGTTT
GCATCATTTTAATAGGTCCCAACGTTGGAACTTATAATTGTGAGTCAACAGATCGTATTT
CGCAATAGGTTACTTAGCAAGG
>EcyBCdTP1_TRINITY_DN506939_c1_g6_i1
AGTGACATGAAGCACTGGCCCTTCGAGGTGGTCAATGAGAACACCAAGCCCAAAATCAGT
GTGGACTACAAAGGTGAGAAGAAGACCTTCACCCCTGAAGAGATCTCCTCTATGGTCCTC
ACCAAGATGAAGGAGACTGCTGATGCTTACCTCGGCACTAACATCAAGGACGCCGTTGTC
ACAGTGCCAGCTTATTTCAACGATTCTCAGCGTCAGGCAACAAAGGATGCTGGTACCATC
TCTGGCCTCAACGTTCTGAGGATTATCAACGAACCCACGGCGGCCGCTATTGCCTACGGT
CTTGACAAGAAGGTCGGAGGCGAGCGCAACGTCCTTATCTTCGATTTGGGCGGTGGTACT
TTCGATGTGTCTATCCTGACAATTGAGGATGGCATCTTTGAGGTGAAGTCTACTGCTGGA
GACACCCATCTTGGTGGTGAAGATTTCGACAATCGCATGGTCAACCACTTCATGCAAGAG
TTCAAGCGCAAGTACAAGAAGGATCTCTCTGAGAATAAGCGTTCACTGAGGAGGCTCAGA
ACTGCCTGCGAGCGCGCAAAGCGTACCCTTTCCTCTTCAACCCAGGCCAGTATCGAGATT
GATTCTCTTTACGATGGTATCGACTTCTATACCTCGGTCACACGTGCTCGATTCGAAGAG
CTGTGCGCCGATCTTTTCCGTGGTACCCTCGACCCCGTCGAGAAGTCTCTCCGTGATGCT
AAGCTCGACAAGGGCCAGATCCAGGAAATCGTACTTGTTGGAGGCTCCACTCGTATCCCC
AA

>EcyBCdTP1_TRINITY_DN510477_c2_g1_i1
GTGCGTGGGCGTGTTCCAGCATGGCAAGGTCGAGATCATCGCCAACGACCAGGGTAACAG
GACCACTCCTTCCTACGTCGCCTTCACCGACACCGAGAGGCTCATAGGAGACGCCGCCAA
GAACCAGGTGGCTCTGAACCCCACCAACACCGTCTTTGATGCCAAGAGGCTCATCGGACG
CAAGTTCGGCGACTCTTCCGTCAACAAGGACATGGAACACTGGCCCTTTAAGGTGGTCAA
CGATGGAGGAAAGCCTATGATCGGCGTGGAGTTCAAGGGGGAGAG
>EcyBCdTP1_TRINITY_DN510477_c2_g2_i4
CTTCGGGACGCAAAGATGGACAAGGGACAGATCCACGAGATCGTACTGGTTGGCGGATCA
ACTCGCATTCCCAAGATCCAGAAGCTGCTTCAAGATTTCTTCAATGGCAAAGAGCTCAAT
AAATCCATCAACCCCGACGAGGCTGTGGCCTACGGGGCGGCCGTCCAGGCGGCTATTCTG
ACAGGCGACAATTCAGAGGCTGTGCGAGACCTGCTGCTGTTGGATGTGGCTCCCCTTTCC
ATGGGAATTGAAACCGCTGGAGGTGTGATGACCACACTGATCAAACGTAACACGACAATC
CCGACGAAAACTTCACAGGTTTTCTCGACCTACGCCGACAATCAGCCTGGTGTTCTGATC
CAGGTGTATGAAGGAGAACGCGCGATGACCCGAGATAACAATCTCCTCGGTAAGTTTGAG
CTTTCTGGACTTCCACCAGCTCCAAGGGGAGTGCCGCAGATTGAAGTCACTTTCGACATC
GACGCCAACGGCATTTTGAACGTGTCAGCCGTTGACAAGTCCACTGGCAAAGAGAACAAG
ATCACCATCACCAACGACAAAGGACGTCTTAGCAAAGAAGACATCGAACGGCTGGTCCAA
GACGCCGAAAAGTACAAAGCAGACGACGATCTACAGAGAGAGAAAATTGCTGCCAAGAAT
GGCCTGGAGTCCTATTGCTTCAACATGAAGTCCACGGTTGAGGACGACAAGGTGAAGGAC
AAGATCAGCGAGTCCGATCGCAGCAAAGTGTTGGAGGCTTGCAATGAAGTCCTCAAGTGG
CTGGACGCCAACCAGCTCGGCGAGAAGGACGAGTACGAGCACAAGCAGAAGGAGGTAGAG
AAGGTGTGCTCCCCAATCATCAGCCAGCTGTACGGAGGGGGAGCTGCTGGCTGCTCGCCG
GGGGCTGGGCCCATGCCGGGGGCAGCTAATGGGACAGGACCCACCATAGAAGAAGTAGAC
TAAGCGTATAAAACTCATTAGTTAATACCATGTTCATTCAGAGTCATTGTAATTATCATT
CCGTATTGTTACATTTGCCATACTAGTCTTTACATCGCATAAAGTGATTCAGTTGTTGGT
GCTTTGAGTTATATATGCTGTTTATTTGCATACTGAGTAGTTTGTTCGTTAAATATCGTG
TCATCCTTTGCGCGTTCAGTCGGATTGTTCGGGCTTTTTTTTGTAAATTATTGTACTTTA
TTATCAGAATTCAACATCCAATGTGTTATGCTGCAAGAAGAGCATTTATCACCGAATGAA
TGAATGTATGTTCGTAATTATTTATTGTAATATTAATGCAATGGAAGAAAGATTTATGAA
TATAAACATATTTTCTAAAAAAAAAAAAAAAAAAAAAAAAAAAAAAAA
>EcyBCdTP1_TRINITY_DN510477_c2_g3_i1
AAAGAAGTGAAGGATGCCGTAATCACGGTGCCGGCCTACTTCAACGACTCTCAGCGCCAA
GCCACCAAGGATGCCGGTGCCATCGCTGGTCTCAACGTGCGTCGGATCATCAACGAACCA
ACAGCCGCCGCCATCGCATACGGTCTTGACAAGAAAGGGAAAGGCGAAAAGAACATCCTG
ATCTTCGACTTGGGTGGAGGAACATTTGATGTATCTATTCTAACAATTGATGATGGCATC
TTTGAAGTCAAAGCTACAGCTGGCGACACCCATCTTGGAGGAGAGGACTTCGACAATCGC
TTGGTGGACCACTTCGCCCAAGAGTTCAAGAGGAAGTACAAGAAGGACATCACACAAAAC
AAGCGCGCTCTGAGACGCCTGAGAACAGCGTGCGAGAGAGCCAAGAGAACACTGTCCTCG
TCCGCTCAAGCCAGCATTGAGATCGACTCCCTCTATGAAGGTACTGACTTCTATACGTCC
ATCACTCGAGCAAGGTTCGAAGAGATGTGCTCGGATCTTTTCAGAGGAACGTTGGAACCT
GTGGAGAAATCCCTTCGGGACGGCAA
>EcyBCdTP1_TRINITY_DN510477_c2_g3_i2
GTGGAGTTCAAGGGGGAGAGCAAGACCTTCTCACCAGAGGAGATCTCTTCCATGGTCCTG
ACAAAGATGAGGGAAATATCGGAGGCTTATCTAGGTAAAGAAGTGAAGGATGCCGTAATC
ACGGTGCCGGCATACTTCAACGACTCTCAGCGCCAAGCCACCAAGGATGCCGGTGCCATC
GCTGGTCTCAACGTGCTTCGGATCATCAACGAACCAACTGCCGCTGCCATCGCGTACGGC
CTTGACAAGATAGAGAGTGGCGAGAAAAACATCCTGATCTTCGACTTGGGCGGAGGAACA
TTTGATGTATCTATTCTAACAATTGATGATGGCATCTTTGAAGTCAAAGCTACAGCTGGC
GACACCCATCTTGGAGGAGAGGACTTTGACAATCGCTTGGTGGACCACTTCGCCCAAGAG
TTCAAGAGGAAGTACAAGAAGGACATCACACAAAACAAGCGCGCTCTGAGACGCCTGAGA
ACAGCGTGCGAGAGAGCCAAGAGAACACTGTCCTCGTCCGCTCAAGCCAGCATTGAGATC
GACTCCCTCTATGAAGGTACTGACTTCTATACGTCCATCACTCGAGCAAGGTTCGAAGAG
ATGTGCTCGGATCTTTTCAGAGGAACGTTGGAACCTGTGGAGAAATCCCTTCGGGACGGC
AA

Supplementary Data 2. Multiple sequence alignment for Fig. 5.

```
>Fenneropenaeus_chinensis_FJ167398
-MA-GPVIGIDLGTTYSCVGVFQQGKVEILANDQGNRTTPSYVAFGDAERLVGDAAKNQA
AMNPCNTVFDAKRLIGRKFEDAAVQSDMKHWPFRVVSEDGKAKISVRFKGEDKSFNPEEI
SSVVLAKMKETAEAYLGNSVKNAVITVPAYFNDSQRQATKDAGTIAGINVLRIINEPTAA
AIAYGLDKQ--SVGK--AERNVLIIDLGGGT-FDVSILSIDDG-VFEVKSTAGDTHLGGE
DFDNRMVTHFVQEFQRKYKKDLTSNKRALRRLRTACERAKRTLSSSTQASLEIDSLFEGI
DYYTSITRARFEELCSDLFRGTLEPVEKALRDAKLDKSSIHDIVLVGGSTRIPKVQKLLQ
GFFNGKELNKSINPDEAVAYGAAVQAAILRGDQSEAVKDMLLLDVAPLSLGIETAGGVMT
ALIKRNTTIPTKHSQIFTTYSDNQPGVSIQVYEGERTMTRDNNLLGKFELSGIAPAPRGV
PQIEVTFDIDANGILNVSAVDKSTGKENRISINNDKGRLSKEEIERMVNEAENYREEDAK
QRERIEAKNRLEALCYSIKSSLSDTAVADKLPSDEKRLVEEKAEETLKWLDNNQLAEKEE
YEYKVKELEQVWRPLASKLH---GSGT------------G--------------------
---------------SSG---------TGPTVEEVD
>Cherax_destructor_KR058822
-MS-APAIGIDLGTTYSCVGVFQQGKVEIIANDQGNRTTPSYVAFTDTERLIGDAAKNQA
ALNPNNTIFDAKRLIGRKFNDPTVQTDKKHWPFDVVSDNGKPKIRVEYKGESKSFNPEEI
SSMVLIKMKETAEAYLGQKVKDAVITVPAYFNDSQRQATKDAGVIAGLNVLRIINEPTAA
AIAYGLDKKAGTEDK--RERNVLIFDLGGGT-FDVSVLSIDDG-IFEVKSTAGDTHLGGE
DFDNRIVNHFMQEFQRKYKKDISSNKRALRRLRTACERAKRTLSSSTQANVEIDSLFEGI
DFYSSITRARFEELCSDLFRNTLLPVEKALRDAKMDKSQIHDIVLVGGSTRIPKVQKLLQ
DFFNGKNLNKSINPDEAVAYGAAVQGAILRGDQSDGVKDLLLLDVAPLSLGIETAGGVMT
ALIKRNTTIPTKQTQIFTTYSDNQPGVLIQVYEGERAMTKDNNLLGKFELSGIPPAPRGV
PQIEVTFDIDANGILNVSAEDKSTGKQNKITITNDKGRLSKEEIDRMVQEAEKYSEEDSR
QRERIESKNRLESLCLNLKSSLSEESVSSKLTEEEKKSVTAQVEETLVWLDNNQLAEKEE
FESQLSKLEDHWKPLAAKIY---GSGA------------P---------------SSAGR
GPV--PPN---T---SSR---------PGPTIEEVD
>Pontastacus_leptodactylus_GAFY01019070
-MA-AQIIGIDLGTTYSCVGVFQQGKVEIIANDQGNRTTPSYVAFTDTERLIGDAAKNQT
ALNPSNTIFDAKRLIGRKFNDPTVQSDKKHWPFNVVDCNGKPKIQVEYKGEKKTFNPEEI
SSMVLTKMKETAEAYLGQKVKDAVITVPAYFNDSQRQATKDAGVIAGLNVLRIINEPTAA
AIAYGLDKKVASGNS--KERNVLIFDLGGGT-FDVSILTIDEG-IFEVKSTAGDTHLGGE
DFDNRMVSHFMQEFQRKYKKDISGNKRSVRRLRTACERAKRTLSSSTQANVEIDSMFEGI
DFYSSITRARFEELCSDLFRNTMLPVEKALKDAKMDKSKVDDIVLVGGSTRIPKVQKLLQ
DFFNGKDLNRSINPDEAVAYGAAVQGAILRGDQTDGVKDLLLLDVAPLSLGIETVGGVMT
ALIKRNTTIPTKQTQVFTTNADNQPGVLIQVYEGERTMTKDNSWLGKFELSGIPPAPRGV
PQVDVTFDIDANGILNVSAEDKSTGKQNKITITNDKGRLSKEEIDRMVQEAEKYSEEDCR
QRERIESKNSLESMCLDLKKSLGEEGISSKLTEEEKKAVTSLVEETLAWIDSNQMAEKDE
FESQLKKLEDHWKPLVAKIY---GQGS------------R---------------GPAGR
GPAGRKPA---TNSSSSG---------PGPTIEEVD
>Drosophila_melanogaster_Hsp70Aa_D__NP_731651.1
----MPAIGIDLGTTYSCVGVYQHGKVEIIANDQGNRTTPSYVAFTDSERLIGDPAKNQV
AMNPRNTVFDAKRLIGRKYDDPKIAEDMKHWPFKVVSDGGKPKIGVEYKGESKRFAPEEI
SSMVLTKMKETAEAYLGESITDAVITVPAYFNDSQRQATKDAGHIAGLNVLRIINEPTAA
ALAYGLDKN--L--K--GERNVLIFDLGGGT-FDVSILTIDEGSLFEVRSTAGDTHLGGE
DFDNRLVTHLADEFKRKYKKDLRSNPRALRRLRTAAERAKRTLSSSTEATIEIDALFEGQ
DFYTKVSRARFEELCADLFRNTLQPVEKALNDAKMDKGQIHDIVLVGGSTRIPKVQSLLQ
DFFHGKNLNLSINPDEAVAYGAAVQAAILSGDQSGKIQDVLLVDVAPLSLGIETAGGVMT
KLIERNCRIPCKQTKTFSTYADNQPGVSIQVYEGERAMTKDNNALGTFDLSGIPPAPRGV
PQIEVTFDLDANGILNVSAKEMSTGKAKNITIKNDKGRLSQAEIDRMVNEAEKYADEDEK
HRQRITSRNALESYVFNVKQAV-EQAPAGKLDEADKNSVLDKCNDTIRWLDSNTTAEKEE
FDHKLEELTRHCSPIMTKMH---QQGA------------G---------------AGAGG
------PG---A---NCGQQAGGFGGYSGPTVEEVD
>Charybdis_japonica_KM277361
-MSRGAAVGIDLGTTYSCVGVFQHGKVEIIANDQGNRTTPSYVAFTDTERLIGDAAKNQV
AMNPNNTVFDAKRLIGRKFNDHHIQSDMKHWPFEVIDDSTKPKIRVEYKGEKKSFYPEEI
SSMVLMKMKETAEAYLGAAVKDAVITVPAYFNDSQRQATKDAGTISGVNVLRIINEPTAA
AIAYGLDKK--V--G--GERNVLIFDLGGGT-FDVSILTIEDG-IFEVKSTAGDTHLGGE
DFDNRMVNHFLQEFKRKYKKDPSESKRALRRLRTACERAKRTLSSSTQASVEIDSLFEGI
DFYTSVTRARFEELCADLFRGTLEPVEKALRDAKLDKAQIHDIVLVGGSTRIPKIQKLLQ
DFFNGKELNKSINPDEAVAYGAAVQAAILCGDKSEAVQDLLLLDVTPLSLGIETAGGVMT
ALIKRNTTIPTKQTQTFTTYSDNQPGVLIQVYEGERAMTKDNNLLGKFELTGIPPAPRGV
PQIEVTFDIDANGILNVPAVDKSTGKENKITITNDKGRLSKEEIERMVQDAEKYKAEDEK
QRDRIGAKNALESYCFNMKSTVEEEKFKDKVSEEDRNKILEACNEAIKWLDANQLGEKDE
YEHKQKELEQICNPIITKMYQAAGG-AP---------PGGMPGGFPGA--G---GA---P
GG---APG-------GGS---------SGPTIEEVD
>Scylla_serrata_JQ780845
-MSKGAAVGIDLGTTYSCVGVFQHPKVEIIANDQGNRTTPSYVAFTDTERLIGDAAKNQV
AMNPNNTVFDAKRLIGRKFNDHHIQSDMKHWPFEVIDDSTKPKIRVEYKGEKKSFYPEKI
```

```
SSMVLMKMKKTAEAYLGAAVKDAVITVPAYFNDSQRQATKDAGTISGVNVLRIIPEPTAA
AIAYGLDKK--V--G--GERNVLIFDLGGGT-FDVSILTIEDG-IFEVKSTAGDTHLGGE
DFDNRMVNHFLQEFKRKYKKDPSESKRALRRLRTACERAKRTLSSSTQASVEIDSLFEGI
DFYTSVTRARFEELCADLFRGTLEPVEKALRDAKLDKAQIHDIVLVGGSTRIPKIQKLLQ
DFFNGKELNKSINPDEAVAYGAAVQAAILCGDKSEAVQDLLLLDVTPLSLGIETAGGVMT
ALIKRNTTIPTKQTQTFTTYSDNQPGVLIQVYEGERAMTKDNNLLGKFELTGIPPAPRGV
PQIEVTFDIDANGILNVSAVDKSTGKENKITITDDKGRLSKEEIERMVQDAEKYKAEDEK
QRDRIGAKNALESYCFNMKSTVEEEKFKDKVSEEDRNKILEACNEAIKWLDANQLGEKEE
YEHKQKDLEQICNPIITKMYQAAGG-AP---------PGGMPGGFPGA--G---GA---P
GG--AAPG-------GGS---------SGPTIEEVD
>Scylla_paramamosain_EU754021
-MSKGAAVGIDLGTTYSCVGVFQHGKVEIIANDQGNRTTPSYVAFTDTERLIGDAAKNQV
AMNPNNTVFDAKRLIGRKFNDHHIQSDMKHWPFEVIDDSTKPKIRVEYKGEKKSFYPEEI
SSMVLMKMKETAEAYLGAAVKDAVITVPAYFNDSQRQATKDAGTISGVNVLRIINEPTAA
AIAYGLDKK--V--G--GERNVLIFDLGGGT-FDVSILTIEDG-IFEVKSTAGDTHLGGE
DFDNRMVNHFLQEFKRKYKKDPSESKRALRRLRTACERAKRTLSSSTQASVEIDPLFEGI
DFYTSVTRARFEELCADLFRGTLEPVEKALRDAKLDKAQIHDIVLVGGSTRIPKIQKLLQ
DFFNGKELNKSINPDEAVAYGAAVQAAILCGDKSEAVQDLLLLDVTPLSLGIETAGGVMT
ALIKRNTTIPTKQTQTFTTYSDNQPGVLIQVYEGERAMTKDNNLLGKFELTGIPPAPRGV
PQIEVTFDIDANGILNVSAVDKSTGRENKITITNDKGRLSKEEIERMVQDAEKYKAEDDK
QRDRIGAKNALESYCFNMKSTVEEEKFKDKVSEEDRNKILEACNEAIKWLDANQLGEKDE
YEHKQKELEQICNPIITKMYQAAGG-AP---------PGGMPGGFPGA--G---GA---P
GG---APG-------GGS---------SGPTIEEVD
>Scylla_paramamosain_JX913782
-MSKGAAVGIDLGTTYSCVGVFQHGKVEIIANDQGNRTTPSYVAFTDTERLIGDAAKNQV
AMNPNNTVFDAKRLIGRKFNDHHIQSDMKHWPFEVIDDSTKPKIRVEYKGEKKSFYPEEI
SSMVLMKMKETAEAYLGAAVKDAVITVPAYFNDSQRQATKDAGTISGVNVLRIINEPTAA
AIAYGLDKK--V--G--GERNVLIFDLGGGT-FDVSILTIEDG-IFEVKSTAGDTHLGGE
DFDNRMVNHFLQEFKRKYKKDPSESKRALRRLRTACERAKRTLSSSTQASVEIDPLFEGI
DFYTSVTRARFEELCADLFRGTLEPVEKALRDAKLDKAQIHDIVLVGGSTRIPKIQKLLQ
DFFNGKELNKSINPDEAVAYGAAVQAAILCGDKSEAVQDLLLLDVTPLSLGIETAGGVMT
ALIKRNTTIPTKQTQTFTTYSDNQPGVLIQVYEGERAMTKDNNLLGKFELTGIPPAPRGV
PQIEVTFDIDANGILNVSAVDKSTGRENKITITNDKGRLSKEEIERMVQDAEKYKAEDDK
QRDRIGAKNALESYCFNMKSTVEEEKFKDKVSEEDRNKILEACNEAIKWLDANQLGEKDE
YEHKQKELEQICNPIITKMYQAAGG-AP---------PGGMPGGFPGA--G---GA---P
GG---APG-------GGS---------SGPTIEEVD
>Portunus_trituberculatus_FJ527835
-MSKGAAVGIDLGTTYSCVGVFQHGKVEIIANDQGNRTTPSYVAFTDTERLIGDAAKNQV
AMNPNNTVFDAKRLIGRKFTDHHVQSDMKHWPFEVIEDSTKPKIRVEYKGEKKSFYPEEI
SSMVLMKMKETAEAYLGAAVKDAVITVPAYFNDSQRQATKDAGTISGVNVLRIINEPTAA
AIAYGLDKK--V--G--GERNVLIFDLGGGT-FDVSILTIEDG-IFEVKSTAGDTHLGGE
DFDNRMVNHFLQEFKRKYKKDPTESKRALRRLRTACERAKRTLSSSTQASVEIDSLFEGI
DFYTSVTRARFEELCADLFRGTLEPVEKALRDAKLDKAQIHDIVLVGGSTRIPKTQKLLQ
DFFNGKELNKSINPDEAVAYGAAVQAAILCGDKSEAVQDLLLLDVTPLSLGIETAGGVMT
ALIKRNTTIPTKQTQTFTTYSDNQPGVLIQVYEGERAMTKDNNLLGKFELTGIPPAPRGV
PQIEVTFDIDANGILNVSAVDKSTGKENKITITNDKGRLSKEEIERMVQDAEKYKAEDDK
QRDRIGAKNALESYCFNMKSTVEEEKFKDKVSEEDRNKILEACNEAIKWLDANQLGEKDE
YEHKQKELEQICNPIIAKMYQAAGG-AP---------PGGMPGGFPGA--G---GA---P
GG---APG-------GGS---------SGPTIEEVD
>Callinectes_sapidus_DQ663760
-MSKGAAVGIDLGTTYSCVGVFQHGKVEIIANDQGNRTTPSYVAFTDTERLIGDAAKNQV
AMNPNNTVFDAKRLIGRKFTDHHVQSDMKHWPFEVIEDSTKPKIRVEYKGEKKSFYPEEI
SSMVLMKMKETAEAYLGAAVKDAVITVPAYFNDSQRQATKDAGTISGVNVLRIINEPTAA
AIAYGLDKK--V--G--GERNVLIFDLGGGT-FDVSILTIEDG-IFEVKSTAGDTHLGGE
DFDNRMVNHFLQEFKRKYKKDPTESKRALRRLRTACERAKRTLSSSTQASVEIDSLFEGI
DFYTSVTRARFEELCADLFRGTLEPVEKALRDAKLDKAQIHDIVLVGGSTRIPKIQKLLQ
DFFNGKELNKSINPDEAVAYGAAVQAAILCGDKSEAVQDLLLLDVTPLSLGIETAGGVMT
ALIKRNTTIPTKQTQTFTTYSDNQPGVLIQVYEGERAMTKDNNLLGKFELTGIPPAPRGV
PQIEVTFDIDANGILNVSAVDKSTGKENKITITNDKGRLSKEEIERMVQDAEKYKAEDDK
QRDRIGAKNALESYCFNMKSTVEEEKFKDKVSEEDRNKILEACNEAIKWLDANQLGEKDE
YEHKQKELEQICNPIITKMYQAAGG-AP---------PGGMPGGFPGA--G---GA---P
GG---APG-------GGS---------SGPTIEEVD
>Chaceon_affinis_KU613091
-MAKGAAVGIDLGTTYSCVGVFQHGKVEIIANDQGNRTTPSYVAFTDTERLIGDAAKNQV
AMNPNNTVFDAKRLIGRKFNDHNVQSDMKHWPFEVVDDSTKPRIRVEYKGEKKSFYPEEI
SSMVLMKMKETAEAYLGSAVKDAVITVPAYFNDSQRQATKDAGTISGVNVLRIINEPTAA
AIAYGLDKK--V--G--GERNVLIFDLGGGT-FDVSILTIEDG-IFEVKSTAGDTHLGGE
DFDNRMVNHFHQEFKRKYKKDPAESKRALRRLRTACERAKRTLSSSTQASVEIDSLFEGI
DFYTSVTRARFEELCADLFRGTLEPVEKALRDAKMDKAQIHDIVLVGGSTRIPKIQKLLQ
DFFNGKELNKSINPDEAVAYGAAVQAAILCGDKSEAVQDLLLLDVTPLSLGIETAGGVMT
ALIKRNTTIPTKQTQTFTTYSDNQPGVLIQVYEGERAMTKDNNLLGKFELTGIPPAPRGV
```

PQIEVTFDIDANGILNVSAVDKSTGKENKITITNDKGRLSKEDIERMVQDAEKYKCEDEK
QRDRIGAKNALESYCFNMKSTVEEEKFKDKVSDEDRNKILEACNEAIKWLDANQLGEKDE
YEHKQKEIEQICNPIITKMYAAAGG-PP---------PGGMPG-FPGA--GA-GGA---P
GGGGAAPG-------GGS---------SGPTIEEVD
>Xantho_pilipes_KU613099
-MAKGAAVGIDLGTTYSCVGVFQHGKVEIIANDQGNRTTPSYVAFTDTERLIGDAAKNQV
AMNPNNTVFDAKRLIGRKFADHNVQADMKHWPFDVIDDSTKPRIRVEYKGEKKSFYPEEI
SSMVLMKMKETAEAYLGAAVKDAVITVPAHFNDSQRQATKDAGTISGVNVLRIINEPTAA
AIAYGLDKK--V--G--GERNVLIFDLGGGT-FDVSILTIEDG-IFEVKSTAGDTHLGGE
DFDNRMVNHFLQEFKRKYKKDPSESKRALRRLRTACERAKRTLSSSTQASVEIDSLFEGI
DFYTSVTRARFEELCADLFRGTLEPVEKALRDAKLDKAQIHDIVLVGGSTRIPKIQKLLQ
DFFNGKELNKSIKPDEAVAYGAAVQAAILCGDKSEAVQDLLLLDVTPLSLGIETAGGVMT
ALIKRNTTIPTKQTQTFTTYSDNQPGVLIQVYEGERAMTKDNNLLGKFELTGIPPAPRGV
PQIEVTFDIDANGILNVSAVDKSTGKENKITITNDKGRLSKEEIERMVQDAEKYKAEDEK
QRDRIGAKNALESYCFNMKSTVEEEKFKDKVSDEDRNKILEACNDAIKWLDANQLGEKDE
YEHKQKEIEQICNPIITKMYQAAGG-AP---------PGGMPGGFPGAPGGAPGGA---P
GGGG------------GS---------SGPTIEEVD
>Cyanagraea_praedator_KU613100
-MAKGAAVGIDLGTTYSCVGVFQHGKVEIIANDQGNRTTPSYVAFTDTERLIGDAAKNQV
AMNPNNTVFDAKRLIGRKFADHNVQADMKHWPFDVIDDSTKPRIRVEYKGEKKSFYPEEI
SSMVLMKMKETAEAYLGAAVKDAVITVPAYFNDSQRQATKDAGTISGVNVLRIINEPTAA
AIAYGLDKK--V--G--GERNVLIFDLGGGT-FDVSILTIEDG-IFEVKSTAGDTHLGGE
DFDNRMVNHFLQEFKRKYKKDPSESKRALRCLRTACERAKRTLSSSTQASVEIDSLFEGI
DFYTSVTRARFEELCADLFRGTLEPVEKALRDAKLDKAQIHDIVLVGCSTRIPKIQKLLQ
DFFNGFELNKSINPDEAVAYGAAVQAAILCGDKSEAVQDLLLLDVTPLSLGIETAGDVMT
ALIKRNTTIPTKQTQTFNTYSDNQPGVLIQVYEGERAMTKDNNLLGKFELTGIPPAPRGV
PQIEVTFDIDANGIVNVCAVDKSTGKENKITITNDKGRLSKEEIERMVQDAEKYKAEDEK
QRDRIGAKNALESYCFNMKSTVEEEKFKDKVSDEDRNKILEACNDAIKWLDANQLGEKDE
YEHKQKEIEQICNPIITKMYQAAGG-AP---------PGGMPGGFPGAPGGAPGGA---P
GGGG------------GS---------SGPTIEEVD
>Segonzacia_mesatlantica_KU613101
-MAKGAAVGIDLGTTYSCVGVFQHGKVEIIANDQGNRTTPSYVAFTDTERLIGDAAKNQV
AMNPNNTVFDAKRLIGRKFADHNVQADMKHWPFDVIDDSTKPRIRVEYKGEKKSFYPEEI
SSMVLMKMKETAEAYLGAAVKDAVITVPAYFNDSQRQATKDAGTISGVNVLRIINEPTAA
AIAYGLDKK--V--G--GERNVLIFDLGGGT-FDVSILTIEDG-IFEVKSTAGDTHLGGE
DFDNRMVNHFLQEFKRKYKKDPSESKRALRRLRTACERAKRTLSSSTQASVEIDSLFEGI
DFYTSITRARFEELCADLFRGTLEPVEKALRDAKLDKAQIHDIVLVGGSTRIPKIQKLLQ
DFFNGKELNKSINPDEAVAYGAAVQAAILCGDKSEAVQDLLLLDVTPLSLGIETAGGVMT
ALIKRNTTIPTKQTQTFTTYSDNQPGVLIQVYEGERAMTKDNNLLGKFELTGIPPAPRGV
PQIEVTFDIDANGILNVSAVGKSTGKENKITITNDKGRLSKEEIERMVQDAEKYKAEDEK
QRDRIGAKNALESYCFNMKSTVEEEKFKDKVSDEDRNKILEACNDAIKWLDANQLGEKDE
YEHKQKEIEQICNPIITKMYQAAGG-AP---------PGGMPGGFPGAPGGAPGGA---P
GGGG------------GS---------SGPTIEEVD
>Cardisoma_armatum_KU613075
-MAKGAAVGIDLGTTYSCVGVFQHGKVEIIANDQGNRTTPSYVAFTDTERLIGDAAKNQV
AMNPNNTVFDAKRLIGRKFNDHTVQSDMKHWPFDVIDDHTKPKIKVDYKGETKSFYPEEI
SSMVLIKMKETAEAYLGTTVKDAVITVPAYFNDSQRQATKDAGTISGLNVLRIINEPTAA
AIAYGLDKK--V--G--GERNVLIFDLGGGT-FDVSILTIEDG-IFEVKSTAGDTHLGGE
DFDNRMVNHFLQEFKRKYKKDPSESKRALRRLRTACERAKRTLSS-AQASVEIDSLFEGI
DFYTSITRARFEELCADLFRGTLEPVEKSLRDAKMDKAQIHDIVLVGGSTRIPKIQKLLQ
DFFNGKELNKSINPDEAVAYGAAVQAAILCGDKSEAVQDLLLLDVTPLSLSIETAGGVMT
ALIKRNTTIPTKQTQTFTTYSDNQPGVLIQVYEGERAMTKDNNLLGKFELTGIPPAPRGV
PQIEVTFDIDANGILNVSAVDKSTGKENKITITNDKGRLSKEEIERMVQDAEKYKAEDEK
QRDRIGAKNALESYCFNMKSTVEEEKFKDKVSEEDRNKILEACNDAIKWLDANQLGEKEE
YEHKQKDLEQICNPIITKMYQAAGG-AP---------PGGMPGGFPGA--G---GA---P
-G--AAPG-------GGS---------SGPTVEEVD
>Cardisoma_armatum_KU613076
-MAKGAAVVIDLGTTYSCVGVFQHGKVEIIANDQGNRTTPSYVAFTDTERLIGDAAKNQV
AMNPNNTVFDAKRLIGRKFNDHTVQSDMKHWPFDVIDDHTKPKIKVDYKGETKSFYPEEI
SSMVLIKMKETAEAYLGTTVKDAVITVPAYFNDSQRQATKDAGTISGLNVLRIINEPTAA
AIAYGLDKK--V--G--GERNVLIFDLGGGT-FDVSILTIEDG-IFEVKSTAGDTHLGGE
DFDNRMVNHFLQEFKRKYKKDPSESKRALRRLRTACERAKRTLSS-AQASVEIDSLFEGI
DFYTSITRARFEELCADLFRGTLEPVEKSLRDAKMDKAQIHDIVLVGGSTRIPKIQKLLQ
DFFNGKELNKSINPDEAVAYGAAVQAAILCGDKSEAVQDLLLLDVTPLSLSIETAGGVMT
ALIKRNTTIPTKQTQTFTTYSDNQPGVLIQVYEGERAMTKDNNLLGKFELTGIPPAPRGV
PQIEVTFDIDANGILNVSAVDKSTGKENKITITNDKGRLSKEEIERMVQDAEKYKAEDEK
QRDRIGAKNALESYCFNMKSTVEEEKFKDKVSEEDRNKILEACNDAIKWLDANQLGEKEE
YEHKQKDLEQICNPIITKMYQAAGG-AP---------PGGMPGGFPGA--G---GA---P
-G--AAPG-------GGS---------SGPTVEEVD
>Cardisoma_armatum_KU613077
-MSKGAAVGIDLGTTYSCVGVFQHGKVEIIANDQGSRTTPSYVAFTDTERLIGDAAKNQV

AMNPNNTVFDAKRLIGRKFNDHTVQSDMKHWPFDVIDDNTKPKIKVDYKGETKSFYPEEI
SSMVLIKMKETAEAYLGTTVKDAVITVPAYFNDSQRQATKDAGTISGLNVLRIINEPTAA
AIAYGLDKK--V--G--GERNVLIFDLGGGT-FDVSILTIEDG-IFEVKSTAGDTHLGGE
DFDNRMVNHFLQEFKRKYKKDPSESKRALRRLRTACERAKRTLSSSAQASVEIDSLFEGI
DFYTSITRARFEELCADLFRGTLEPVEKSLRDAKMDKAQIHDIVLVGGSTRIPKIQKLLQ
DFFNGKELNKSINPDEAVAYGAAVQAAILCGDKSEAVQDLLLLDVTPLSLGIETAGGVMT
ALIKRNTTIPTKQTQTFTTYSDNQPGVLIQVYEGERAMTKDNNLLGKFELTGIPPAPRGV
PQIEVTFDIDANGILNVSAVDKSTGKENKITITNDKGRLSKEEIERMVQDAEKYKAEDEK
QGDRIGAKNALESYCFNTKSTVEEEKFKDKVSEEDRNKILEACNDAIKWLDANQLGEKEE
YEHKQKDLEQICNPIITKMYQAAGG-AP---------PGGMPGGSPGA--G---GA---P
-G--AAPG-------GGS---------SGPTIEEVD
>Pachygrapsus_marmoratus_KU613082
-MAKGAAVGIDLGTTYSCVGVFQHGKVEIIANDQGNRTTPSYVAFTDTERLIGDAAKNQV
AMNPNNTVFDAKRPIGRKFNDHNVQSDMKHWPFDVIDDNTKPKIKVEYKGEAKSFYPEEI
SSMVLIKMKETAEAYLGSVVKDAVVTVPAYFNDSQRQATKDAGTISGLDVLRIINEPTAA
AIAYGLDKK--V--G--GERNVLIFDLGGGT-FDVSILTIEDG-IFEVKSTAGDTHLGGE
DFDNRMVNHFLQEFKRKYKKDPSESKRALRRLRTACERAKRTLSSSAQASVEIDSLFEGI
DFYTSITRARFEELCADLFRGTLEPVEKALRDAKMDKAQIHDIVLVGGSTRIPKIQKLLQ
DFFNGKELNKSINPDEAVAYGAAVQAAILCGDKSEAVQDLLLLDVTPLSLGIETAGGVMT
ALIKRNTTIPTKQTQTFTTYSDNQPGVLIQVYEGERAMTKDNNLLGKFELTGIPPAPRGV
PQIEVTFDIDANGILNVSTVDKSTGKENKITITNDKGRLSKEEIERMVQDAEKYKVEDEK
QRDRIGAKNALESYCFNMKSTVEEDKFKDKVSEEDRNKIMEACNETIKWLDANQLGEKEE
YEHKQKDIEQICNPIITKMYQAAGG-AP---------PGGMPGGFPGA--G---GA---P
-G--AAPG-------GGS---------SGPTIEEVD
>Pachygrapsus_marmoratus_KU613084
-MAKGAAVGIDLGTTYSCVGVFQHGKVEIIANDQGNRTTPSYVAFTDTERLIGDAAKNQV
AMNPNNTVFDAKRLIGRKFNDHNVQSDMKHWPFDVIDDNTKPKIKVEYKGEAKSFYPEEI
SSMVLIKMKETAEAYLGSVVKDAVVTVPAYFNDSQRQATKDAGTISGLNVLRIINEPTAA
AIAYGLDKK--V--G--GERNVLIFDLGGGT-FDVSILTIEDG-IFEVKSTAGDTHLGGE
DFDNRMVNHFLQEFKRKYKKDPSESKRALRRLRTACERAKRTLSSSAQASVEIDSLFEGI
DFYTSITRARFEELCADLFRGTLEPVEKALRDAKMDKAQIHDIVLVGGSTRIPKIQKLLQ
DFFNGKELNKSINPDEAVAYGAAVQAAILCGDKSEAVQDLLLLDVTPLSLGIETAGGVMT
ALIKRNTTIPTKQTQTFTTYSDNQPGVLIQVYEGERAMTKDNNLLGKFELTGIPPAPRGV
PQIEVTFDIDANGILNVSAVDKSTGKENKITITNDKGRLSKEEIERMVQDAEKYKVEDEK
QRDRIGAKNALESYCFNMKSTVEEDKFKDKVSEEDRNKIMEACNETIKWLDANQLGEKEE
YEHKQKDIEQICNPIITKMYQAAGG-AP---------PGGMPGGFPGA--G---GA---P
-G--AAPG-------GGS---------SGPTIEEVD
>Pachygrapsus_marmoratus_KU613083
-MAKGAAVGIDLGTTYSCVGVFQHGKVEIIANDQGNRTTPSYVAFTDTERLIGDAAKNQV
AMNPNNTVFDAKRLIGRKFNDHNVQSDMKHWPFDVIDDNTKPKIKVEYKGEAKSFYPEEI
SSMVLIKMKETAEAYLGSVVKDAVVTVPAYFNDSQRQATKDAGTISGLNVLRIINEPTAA
AIAYGLDKK--V--G--GERNVLIFDLGGGT-FDVSILTIEDG-IFEVKSTAGDTHLGGE
DFDNRMVNHFLQEFKRKYKKDPSESKRALRRLRTACERAKRTLSSSAQASVEIDSLFEGI
DFYTSITRARFEELCADLFRGTLEPVEKALRDAKMDKAQIHDIVLVGGSTRIPKIQKLLQ
DFFNGKELNKSINPDEAVAYGAAVQAAILCGDKSEAVQDLLLLDVTPLSLGIETAGGVMT
ALIRRNTTIPTKQTQTFTTYSDNQPGVLIQVYEGERAMTKDNNLLGKFELTGIPPAPRGV
PQIEVTFDIDANGILNVSAVDKSTGKENKITITNDKGRLSKEEIERMVQDAEKYKVEDEK
QRDRIGAKNALESYCFNMKSTVEEDKFKDKVSEEDRNKIMEACNETIKWLDANQLGEKEE
YEHKQKDIEQICNPIITKMYQAAGG-AP---------PGGMPGGFPGA--G---GA---P
-G--AAPG-------GGS---------SGPTVEEVD
>Pachygrapsus_marmoratus_AM410078
-MAKGAAVGIDLGTTYSCVGVFQHGKVEIIANDQGNRTTPSYVAFTDTERLIGDAAKNQV
AMNPNNTVFDAKRLIGRKFNDHNVQSDMKHWPFDVIDDNTKPKIKVEYEGEAKSFYPEEI
SSMGLIKMKETAEAYLGSVVKDAVVTVPAYFNDSQRQATKDAGTISGLNVLRIISEPTAA
AIAYGLDKK--V--G--GERNVLIFDLGGGT-FDVSILTIEDG-IFEVKSTAGDTHLGGE
DFDNRMVNHFLQEFKRKYKKGSSESKRALRRLRIACERAKRTLSSSAQASVEIDSLFEGI
DFYTSITRARFEELCADLFRGTLEPVKKVLRDAKMDKAQIHDIVLVGGCTRIPKIQKLLQ
DFFNGKELNKSINPDEAVAYGAAVQAAILCGDKSETVQDLLLLDVTPLSLGIETAGGVMT
ALIKRNTTIPTKQTQTFTTYSDNQPGVLIQVYEGERAMTKDNNLLGKFELTGIPPAPRGV
PQIEVTFDIDANGILNVSAVDKSTGKENKITITNDKGRLSKEEIERMVQDAEKYKVEDEK
QRDRIGAKNALESYCFNMKSTVEEDKFKDKVSEEDRNKIMEACNETIKWLDANQLGEKEE
YEHKQKDIEQICSPIITKMYQAAGG-AP---------PGGMPGGFPGA--G---GA---P
-G--AAPG-------GGS---------SGPTIEEVD
>Pachygrapsus_marmoratus_DQ173922
-MSKGAAVGIDLGTTYSCVGVFQHGKVEIIANDQGNRTTPSYVAFTDTERLIGDAAKNQV
AMNPNNTVFDAKRLIGRKFNDHNVQSDMKHWPFDVIDDNTKPKIKVEYKGEAKSFYPEEI
SSMVLIKMKETAEAYLGSVVKDAVVTVPAYFNDSQRQATKDAGTISGLNVLRIINEPTAA
AIAYGLDKK--V--G--GERNVLIFDLGGGT-FDVSILTIEDG-IFEVKSTAGDTHLGGE
DFDNRMVNHFLQEFKRKYKKDPSESKRALRRLRTACERAKRTLSSSAQASVEIDSLFEGI
DFYTSITRARFEELCADLFRGTLEPVEKALRDAKMDKAQIHDIVLVGGSTRIPKIQKLLQ
DFFNGKELNKSINPDEAVAYGAAVQAAILCGDKSEAVQDLLLLDVTPLSLGIETAGGVMT

ALIKRNTTIPTKQTQTFTTYSDNQPGVLIQVYEGERAMTKDNNLLGKFELTGIPPAPRGV
PQIEVTFDIDANGILNVSAVDKSTGKENKITITNDKGRLSKEEIERMVQDAEKYKVEDEK
QRDRIGAKNALESYCFNMKSTVEEDKFKDKVSEEDRNKIMEACNETIKWLDANQLGEKEE
YEHKQKDIEQICNPIITKMYQAAGG-AP---------PGGMPGGFPGA--G---GA---P
-G--AAPG-------GGS---------SGPTIEEVD
>Hemigrapsus_nudus_KU613086
-MAKGAAVGIDLGTTYSCVGVFQHGKVEIIANDQGNRTTPSYVAFTDTERLIGDAAKNQV
AMNPNNTVFDAKRLIGRKFNDHNVQSDMKHWPFDVIDDNTKPKIQVEYKGEAKSFYPEEI
SSMVLIKMKETAEAYLGSIVKDAVITVPAYFNDSQRQATKDAGTISGLNVLRIINEPTAA
AIAYGLDKK--V--G--GERNVLIFDLGGGT-FDVSILTIEDG-IFEVKSTAGDTHLGGE
DFDNRMVNHFLQEFKRKYKKDPSESKRALRRLRTACERAKRTLSSSAQASVEIDSLFEGI
DFYTSITRARFEELCADLFRGTLEPVEKALRDAKMDKAQIHDIVLVGGSTRIPKIQKLLQ
DFFNGKELNKSINPDEAVAYGAAVQAAILCGDKSEAVQDLLLLDVTPLSLGIETAGGVMT
ALIKRNTTIPTKQTQTFTTYSDNQPGVLIQVYEGERAMTKDNNLLGKFELTGIPPAPRGV
PQIEVTFDIDANGILNVSAVDKSTGKENKITITNDKGRLSKEEIERMVQDAEKYKAEDEK
QRDRIGAKNALESYCFNMKSTMGEDKFKDKVSEEDRNKILEACNETIKWLDANQLGEKEE
YEHKQKDIEQICNPIITKMYQAAGG-AP---------PGGMPGSFPGA--G---GA---P
-G--AAPG-------GGS---------SGPTIEEVD
>Pontastacus_leptodactylus_GAFY01000931
-MAKTTAVGIDLGTTYSCVGVFQHGKVEIIANDQGNRTTPSYVAFTDTERLIGDAAKNQV
AMNPNNTVFDAKRLIGRKFDDHNVQADMKHWPFDVINENTKPKIQVNYKGDTKTFYPEEI
SSMVLIKMKETAEAYLGVTVKDAVVTVPAYFNDSQRQATKDAGTISGLNVLRIINEPTAA
AIAYGLDKK--V--G--GERNVLIFDLGGGT-FDVSILTIEDG-IFEVKSTAGDTHLGGE
DFDNRMVNHFIQEFKRKYKKDPTENKRALRRLRTACERAKRTLSSSTQASVEIDSLFEGI
DFYTSITRARFEELCADLFRGTLEPVEKALRDAKMDKAQIHDIVLVGGSTRIPKIQKLLQ
DFFNGKELNKSINPDEAVAYGAAVQAAILCGDKSEAVQDLLLLDVTPLSLGIETAGGVMT
ALIKRNTTIPTKQTQTFTTYSDNQPGVLIQVYEGERAMTKDNNLLGKFELTGIPPAPRGV
PQIEVTFDIDANGILNVSAVDKSTGKENKITITNDKGRLSKEEIERMVQDADKYKAEDEK
QRDRISAKNSLESYCFNMKSTVEEEKFKDKVSEEDRNKIMEACNDAIKWLDANQLGEKEE
YEHKQKEIEQVCNPIITKMYQAAGG-APGGMPGG--MPGGMPGGFPGAP-G---GG---T
-G--AAPG---S---GGS----------GPTIEEVD
>Astacus_astacus_KU613104
-MAKTTAVGIDLGTTYSCVGVFQHGKVEIIANDQGNRTTPSYVAFTDTERLIGDAAKNQV
AMNPNNTVFDAKRLIGRKFDDHNVQADMKHWPFDVINENTKPKIQVNYKGDTKTFYPEEI
SSMVLIKMKETAEAYLGVTVKDAVVTVPAYFNDSQRQATKDAGTISGLNVLRIINEPTAA
AIAYGLDKK--V--G--GERNVLIFDLGGGT-FDVSILTIEDG-IFEVKSTAGDTHLGGE
DFDNRMVNHFIQEFKRKYKKDPTENKRALRRLRTACERAKRTLSSSTQASVKIDSLFEGI
DFYTSITRARFEELCADLFRGTLEPVEKALRDAKMDKAQIHDIVLVGGSTRIPKIQKLLQ
DFFNGKELNKSINPDEAVAYGAAVQAAILCGDKSEAVQDLLLLDVTPLSLGIETAGGVMT
ALIKRNTTIPTKQTQTFTTYSDNQPGVLIQVYEGERAMTKDNNLLGKFELTGIPPAPRGV
PQIEVTFDIDANGILNVSAVDKSTGKENKITITNDKGRLSKEEIERMVQDADKYKAEDEK
QRDRISAKNSLESYCFNMKSTVEEEKFKDKVSEEDRNKIMEACNDAIKWLDANQLGEKEE
YEHKQKEIEQVCNPIITKMYQAAGG-APGGMPGG--MPGGMPGGFPGAP-G---GG---T
-G--AAPG---S---GGS----------GPTIEEVD
>Homarus_americanus_DQ173923
-MAKTTAVGIDLGTTYSCVGVFQHGKVEIIANDQGNRTTPSYVAFTDTERLIGDAAKNQV
AMNPNNTVFDAKRLIGRKFDDHNVQSDMKHWPFEVINENTKPKIQVEYKGDKKTFYPEEI
SSMVLIKMKETAEAYLGTTVKDAVVTVPAYFNDSQRQATKDAGTISGLNVLRIINEPTAA
AIAYGLDKK--V--G--GERNVLIFDLGGGT-FDVSILTIEDG-IFEVKSTAGDTHLGGE
DFDNRMVNHFLQEFKRKYKKDPQENKRALRRLRTACERAKRTLSSSTQASVEIDSLFEGI
DFYTSVTRARFEELCADLFRGTLEPVEKALRDAKMDKAQIHDIVLVGGSTRIPKIQKLLQ
DFFNGKELNKSINPDEAVAYGAAVQAAILCGDKSEAVQDLLLLDVTPLSLGIETAGGVMT
ALIKRNTTIPTKQTQTFTTYSDNQPGVLIQVYEGERAMTKDNNLLGKFELTGIPPAPRGV
PQIEVTFDIDANGILNVSAVDKSTGKENKITITNDKGRLSKEEIERMVQDAEKYKAEDEK
QRDRISAKNSLESYCFNMKSTVEEDKFKDKVSEEDRNKILEACNDAIKWLDANQLGEKEE
YEHKQKEIEQICNPIITKMYQAAGG-AP---PGG--MPGGMPGGFPGAP-G---GA---P
-G--AAPG---G---GGS---------SGPTIEEVD
>Cherax_cainii_KR058813
-MAKARAVGIDLGTTYSCVGVFQHGKVEIIANDQGNRTTPSYVAFTDTERLIGDAAKNQV
AMNPNNTVFDAKRLIGRKFDDHNVQADMKHWPFTVINENTKPKIQVEYKGDKKAFYPEEI
SSMVLIKMKETAEAYLGATVKDAVVTVPAYFNDSQRQATKDAGTISGLNVLRIINEPTAA
AIAYGLDKK--V--G--GERNVLIFDLGGGT-FDVSILTIEDG-IFEVKSTAGDTHLGGE
DFDNRMVNHFMQEFKRKYKKDPSENKRALRRLRTACERAKRTLSSSTQASVEIDSLFEGI
DFYTSITRARFEELCADLFRGTLEPVEKALRDAKMDKAQIQDIVLVGGSTRIPKIQKLLQ
DFFNGKELNKSINPDEAVAYGAAVQAAILCGDKSEAVQDLLLLDVTPLSLGIETAGGVMT
ALIKRNTTIPTKQTQTFTTYSDNQPGVLIQVYEGERAMTKDNNLLGKFELTGIPPAPRGV
PQIEVTFDIDANGILNVSAVDKSTGKENKITITNDKGRLSKEEIERMVQDAEKYKAEDEK
QRERISAKNSLESYCFNMKSTVEEEKFKDKVSEEDRNKILEACNDAIKWLDSNQLGEKEE
YEHKQKEIEQICNPIITQMYQAAGG-APGGMPGG------MPGGFPGAP-G---GA----
----GAPG-------GGS----------GPTIEEVD
>Cherax_quadricarinatus_KR058811

```
-MAKARAVGIDLGTTYSCVGVFQHGKVEIIANDQGNRTTPSYVAFTDTERLIGDAAKNQV
AMNPNNTVFDAKRLIGRKFDDHNVQADMKHWPFTVINENTKPKIQVEYKGDKKTFYPEEI
SSMVLIKMKETAEAYLGATVKDAVITVPAYFNDSQRQATKDAGTISGLNVLRIINEPTAA
AIAYGLDKK--V--G--GERNVLIFDLGGGT-FDVSILTIEDG-IFEVKSTAGDTHLGGE
DFDNRMVNHFMQEFKRKYKKDPSENKRALRRLRTACERAKRTLSSSTQASVEIDSLFEGI
DFYTSITRARFEELCADLFRGTLEPVEKALRDAKMDKSQIQDIVLVGGSTRIPKIQKLLQ
DFFNGKELNKSINPDEAVAYGAAVQAAILCGDKSEAVQDLLLLDVTPLSLGIETAGGVMT
ALIKRNTTIPTKQTQTFTTYSDNQPGVLIQVYEGERAMTKDNNLLGKFELTGIPPAPRGV
PQIEVTFDIDANGILNVSAVDKSTGKENKITITNDKGRLSKEEIERMVQDAEKYKAEDEK
QRDRISAKNSLESYCFNMKSTVEEEKFKDKVSEEDRNKILEACNDAIKWLDSNQLGEKEE
YEHKQKEIEQICNPIITKMYQAAGG-APGGMPGG--MPGGMPGGFPGAP-G---GA----
----GAPG-------GGS----------GPTIEEVD
>Cherax_destructor_KR058812
-MAKARAVGIDLGTTYSCVGVFQHGKVEIIANDQGNRTTPSYVAFTDTERLIGDAAKNQV
AMNPNNTVFDAKRLIGRKFDDHNVQADMKHWPFTVINENTKPKIQVEYKGDKKTFYPEEI
SSMVLIKMKETAEAYLGATVKDAVITVPAYFNDSQRQATKDAGTISGLNVLRIINEPTAA
AIAYGLDKK--V--G--GERNVLIFDLGGGT-FDVSILTIEDG-IFEVKSTAGDTHLGGE
DFDNRMVNHFTQEFKRKYKKDPSENKRALRRLRTACERAKRTLSSSTQASVEIDSLFEGI
DFYTSVTRARFEELCADLFRGTLEPVEKALRDAKMDKAQIQDIVLVGGSTRIPKIQKLLQ
DFFNGKELNKSINPDEAVAYGAAVQAAILCGDKSEAVQDLLLLDVTPLSLGIETAGGVMT
ALIKRNTTIPTKQTQTFTTYSDNQPGVLIQVYEGERAMTKDNNLLGKFELTGIPPAPRGV
PQIEVTFDIDANGILNVSAVDKSTGKENKITITNDKGRLSKEEIERMVQDAEKYKAEDEK
QRDRISAKNSLESYCFNMKSTVEEEKFKDKVSEEDRNKILEACNDAIKWLDSNQLGEKEE
YEHKQKEIEQICNPIITKMYQAAGG-APGGMPGG--MPGGMPGGFPGAP-G---GT----
----GAPG-------GGS----------GPTIEEVD
>Litopenaeus_vannamei_AY645906
-MAKAPAVGIDLGTTYSCVGVFQHGKVEIIANDQGNRTTPSYVAFTDTERLIGDAAKNQV
AMNPNNTVFDAKRLIGRKFEDHTVQSDMKHWPFTIINESTKPKIQVEYKGDKKTFYPEEI
SSMVLIKMKETAEAYLGSTVKDAVVTVPAYFNDSQRQATKDAGTISGLNVLRIINEPTAA
AIAYGLDKK--V--G--GERNVLIFDLGGGT-FDVSILTIEDG-IFEVKSTAGDTHLGGE
DFDNRMVNHFIQEFKRKYKKDPSENKRSLRRLRTACERAKRTLSSSTQASVEIDSLFEGI
DFYTSITRARFEELCADLFRGTLEPVEKSLRDAKMDKAQIHDIVLVGGSTRIPKIQKLLQ
DFFNGKELNKSINPDEAVAYGAAVQAAILCGDKSEAVQDLLLLDVTPLSLGIETAGGVMT
ALIKRNTTIPTKQTQTFTTYSDNQPGVLIQVYEGERAMTKDNNLLGKFELSGIPPAPRGV
PQIEVTFDIDANGILNVSAVDKSTGKENKITITNDKGRLSKEEIERMVQDAEKYKADDEK
QRDRISAKNSLESYCFNMKSTVEDEKFKEKISEEDRNKILETCNETIKWLDMNQLGEKEE
YEHKQKEIEQVCNPIITKMYAAAGG-AP---------PGGMPGGFP----G---GA---P
GAGGAAPG---A---GGS---------SGPTIEEVD
>Litopenaeus_vannamei_EF495128
-MAKAPAVGIDLGTTYSCVGVFQHGKVEIIANDQGNRTTPSYVAFTDTERLIGDAAKNQV
AMNPNNTVFDAKRLIGRKFEDHTVQSDMKHWPFTIINESTKPKIQVEYKGDKKTFYPEEI
SSMVLIKMKETAEAYLGSTVKDAVVTVPAYFNDSQRQATKDAGTISGLNVLRIINEPTAA
AIAYGLDKK--V--G--GERNVLIFDLGGGT-FDVSILTIEDG-IFEVKSTAGDTHLGGE
DFDNRMVNHFIQEFKRKYKKDPSENKRSLRRLRTACERAKRTLSSSTQASVEIDSLFEGI
DFYTSITRARFEELCADLFRGTLEPVEKSLRDAKMDKAQIHDIVLVGGSTRIPKIQKLLQ
DFFNGKELNKSINPDEAVAYGAAVQAAILCGDKSEAVQDLLLLDVTPLSLGIETASGVMT
ALIKRNTTIPTKQTQTFTTYSDNQPGVLIQVYEGERAMTKDNNLLGKFELSGIPPAPRGV
PQIEVTFDIDANGILNVSAVDKSTGKENKITITNDKGRLSKEEIERMVQDAEKYKADDEK
QRDRISAKNSLESYCFNMKSTVEDEKFKEKISEEDRNKILETCNETIKWLDMNQLGEKEE
YEHKQKEIEQVCNPIITKMYAAAGG-AP---------PGGMPGGFP----G---GA---P
GAGGAAPG---A---GGS---------SGPTIEEVD
>Fenneropenaeus_chinensis_AY748350
-MAKAPAVGIDLGTTYSCVGVFQHGKVEIIANDQGNRTTPSYVAFTDTERLIGDAAKNQV
AMNPNNTVFDAKRLIGRKFEDHTVQSDMKHWPFTIINESTKPKIQVEYKGDKKTFYPEEI
SSMVLIKMKETAEAYLGSTVKDAVVTVPAYFNDSQRQATKDAGTISGLNVLRIINEPTAA
AIAYGLDKK--V--G--GERNVLIFDLGGGT-FDVSILTIEDG-IFEVKSTAGDTHLGGE
DFDNRMVNHFIQEFKRKYKKDPSENKRSLRRLRTACERAKRTLSSSTQASVEIDSLFEGI
DFYTSITRARFEELCADLFRGTLEPVEKSLRDAKMDKAQIHDIVLVGGSTRIPKIQKLLQ
DFFNGKELNKSINPDEAVAYGAAVQAAILCGDKSEAVQDLLLLDVTPLSLGIETAGGVMT
ALIKRNTTIPTKQTQTFTTYSDNQPGVLIQVYEGERAMTKDNNLLGKFELSGIPPAPRGV
PQIEVTFDIDASGILNVSAVDKSTGKENKITITNDKGRLSKEEIERMVQDAEKYKADDEK
QRDRISAKNSLESYCFNMKSTVEDEKFKEKISEEDRNKILETCNETIKWLDMNQLGEKEE
YEHKQKEIEQVCNPIITKMYAAAGG-AP---------PGGMPGGFP----G---GA---P
GAGGAAPG---A---GGS---------SGPTIEEVD
>Penaeus_monodon_KJ746596
-MAKAPAVGIDLGTTYSCVGVFQHGKVEIIANDQGNRTTPSYVAFTDTERLIGDAAKNQV
AMNPNNTVFDAKRLIGRKFEDHTVQSDMKHWPFTIINESTKPKIQVEYKGDKKTFYPEEI
SSMVLIKMKETAEAYLGSTVKDAVVTVPAYFNDSQRQATKDAGTISGLNVLRIINEPTAA
AIAYGLDKK--V--G--GERNVLIFDLGGGT-FDVSILTIEDG-IFEVKSTAGDTHLGGE
DFDNRMVNHFIQEFKRKYKKDPSENKRSLRRLRTACERAKRTLSSSTQASVEIGSLFEGI
DFYTSITRARFEELCADLFRGTLEPVEKSLRDAKMDKAQIHDIVLVGGSTRIPKIQKLLQ
```

DFFNGKELNKSINPDEAVAYGAAVQAAILCGDKSEAVQDLLLLDVTPLSLGIETAGGVMT
ALIKRNTTIPTKQTQTFTTYSDNQPGVLIQVYEGERAMTKDNNLLGKFELSGIPPAPRGV
PQIEVTFDIDANGILNVSAVDKSTGKENKITITNDKGRLSKEEIERMVQDAEKYKADDEK
QRDRISAKNSLESYCFNMKSTVEDEKFKEKISEEDRNKILETCNETIKWLDMNQLGEKEE
YEHKQKEIEQVCNPIITKMYAAAGG-AP---------PGGMPGGFP----G---GA---P
GAGGAAPG---A---GGS---------SGPTIEEVD
>Penaeus_monodon_EF472918
-MAKAPAVGIDLGTTYSCVGVFQHGKVEIIANDQGNRTTPSYVAFTDTERLIGDAAKNQV
AMNPNNTVFDAKRLIGRKFEDHTVQSDMKHWPFTIINESTKPKIQVEYKGDKKTFYPEEI
SSMVLIKMKETAEAYLGSTVKDAVVTVPAYFNDSQRQATKDAGTISGLNVLRIINEPTAA
AIAYGLDKK--V--G--GERNVLIFDLGGGT-FDVSILTIEDG-IFEVKSTAGDTHLGGE
DFDNRMVNHFIQEFKRKYKKDPSENKRSLRRLRTACERAKRTLSSSTQASVEIDSLFEGI
DFYTSITRARFEELCADLFRGTLEPVEKSLRDAKMDKAQIHDIVLVGGSTRIPKIQKLLQ
DFFNGKELNKSINPDEAVAYGAAVQAAILCGDKSEAVQDLLLLDVTPLSLGIETAGGVMT
ALIKRNTTIPTKQTQTFTTYSDNQPGVLIQVYEGERAMTKDNNLLGKFELSGIPPAPRGV
PQIEVTFDIDANGILNVSAVDKSTGKENKITITNDKGRLSKEEIERMVQDAEKYKADDEK
QRDRISAKNSLESYCFNMKSTVEDEKFKEKISEEDRNKILETCNETIKWLDMNQLGEKEE
YEHKQKEIEQVCNPIITKMYAAAGG-AP---------PGGMPGGFP----G---GA---P
GAGGAAPG---A---GGS---------SGPTIEEVD
>Penaeus_monodon_AF474375
-MAKAPAVGIDLGTTYSCVGVFQHGKVEIIANDQGNRTTPSYVAFTDTERLIGDAAKNQV
AMNPSNTVFDAKRLIGRKFEDHTVQSDMKHWPFTIINESTKPKIQVEYKGDKKTFYPEEI
SSMVLIKMKETAEAYLGSTVKDAVVTVPAYFNDSQRQATKDAGTISGLNVLRIINEPTAA
GIAYGLDKK--V--G--GERNVLIFDLGGGT-FDVSILTIEDG-IFEVKSTAGDTHLGGE
DFDNRMVNHFIQEFKRKYKKDPSENKRSLRRLRTACERAKRTLSSSTQASVEIDSLFEGI
DFYTSITRARFEELCADLFRGTLEPVEKSLRDAKMDKAQIHDIVLVGGSTRIPKIQKLLQ
DFFNGKELNKSINPDEAVAYGAAVQAAILCGDKSEAVQDLLLLDVTPLSLGIETAGGVMT
ALIKRNTTIPTKQTQTFTTYSDNQPGVLIQVYEGERAMTKDNNLLGKFELSGIPPAPRGV
PQIEVTFDIDANGILNVSAVDKSTGKENKITITNDKGRLSKEEIERMVQDAEKYKADDEK
QRDRISAKNSLESYCFNMKSTVEDEKFKEKISEEDRNKILETCNETIKWLDMNQLGEKEE
YEHKQKEIEQVCNPIITKMYAAAGG-AP---------PGGMPGGFP----G---GA---P
GAGGAAPG---A---GGS---------SGPTIEEVD
>Marsupenaeus_japonicus_AB520826
-MAKAPAVGIDLGTTYSCVGVFQHGKVEIIANDQGNRTTPSYVAFTDTERLIGDAAKNQV
AMNPNNTVFDAKRLIGRKFDDHTVQSDMKHWPFEIINESTKPKIQVEYKGDKKTFYPEEI
SSMVLIKMKETAEAYLGSTVKDAVVTVPAYFNDSQRQATKDAGTISGLNVLRIINEPTAA
AIAYGLDKK--V--G--GERNVLIFDLGGGT-FDVSILTIEDG-IFEVKSTAGDTHLGGE
DFDNRMVNHFIQEFKRKYKKDPSENKRSLRRLRTACERAKRTLSSSTQANVEIDSLFEGI
DFYTSITRARFEELCADLFRGTLEPVEKSLRDAKMDKAHIHDIVLVGGSTRIPKIQKLLQ
DFFNGKELNKSINPDEAVAYGAAVQAAILCGDKSEAVQDLLLLDVTPLSLGIETAGGVMT
ALIKRNTTIPTKQTQTFTTYSDNQPGVLIQVYEGERAMTKDNNLLGKFELSGIPPAPRGV
PQIEVTFDIDANGILNVSAVDKSTGKENKITITNDKGRLSKEEIERMVQDAEKYKADDEK
QRDRISAKNSLESYCFNMKSTVEDEKFKEKISEEDRNKILETCNETIKWLDMNQLGEKEE
YEHKQKEIEQVCNPIITKMYAAAGG-AP---------PGGMPGGFP----G---GA---P
GAGGAAPG---A---GGS---------SGPTIEEVD
>Marsupenaeus_japonicus_EF091692
-MAKAPAVGIDLGTTYSCVGVFQHGKVEIIANDQGNRTTPSYVAFTDTERLIGDAAKNQV
AMNPNNTVFDAKRLIGRKFDDHTVQSDMKHWPFEIINESTKPKIQVEYKGDKKTFYPEEI
SSMVLIKMKETAEAYLGSTVKDAVVTVPAYFNDSQRQATKDAGTISGLNVLRIINEPTAA
TIAYGLDKK--V--G--GERNVLIFDLGGGT-FDVSILTIEDG-IFEVKSTAGDTHLGGE
DFDNRMVNHFIQEFKRKYKKDPSENKRSLRRLRTACERAKRTLSSSTQASVEIDSLFEGI
DFYTSITRARFEELCADLFRGTLEPVEKSLRDAKMDKAQIHDIVLVGGSTRIPKIQKLLQ
DFFNGKELNKSINPDEAVAYGAAVQAAILCGDKSEAVQDLLLLDVTPLSLGIETAGGVMT
ALIKRNTTIPTKQTQTFTTYSDNQPGVLIQVYEGERAMTKDNNLLGKFELSGIPPAPRGV
PQIEVTFDIDANGILNVSAVDKSTGKENKITITNDKGRLSKEEIERMVQDAEKYKADDEK
QRDRISAKNSLESYCFNMKSTVEDEKFKEKISEEDRNKILETCNETIKWLDMNQLGEKEE
YEHKQKEIEQVCNPIITKMYAAAGG-AP---------PGGMPGGFP----G---GA---P
GAGGAAPG---A---GGS---------SGPTIEEVD
>Metapenaeus_ensis_DQ486134
-MSKASAVGIDLGTTYSCVGVFQHGKVEIIANDQGNRTTPSYVAFTDTERLIGDAAKNQV
AMNPNNTVFDAKRLIGRKFDDATVQSDMKHWPFTIVNESTKPKIQVEYKGDKKTFYPEEI
SSMVLIKMKETAEAYLGATVKDAVVTVPAYFNDSQRQATKDAGTISGLNVLRIINEPTAA
AIAYGLDKK--V--G--GERNVLIFDLGGGT-FDVSILTIEDG-IFEVKSTAGDTHLGGE
DFDNRMVNHFIQEFKRKYKKDPSENKRSLRRLRTACERAKRTLSSSTQASVEIDSLFEGI
DFYTSITRARFEELCADLFRGTLEPVEKSLRDAKMDKAQIHDIVLVGGSTRIPKIQKLLQ
DFFNGKELNKSINPDEAVAYGAAVQAAILCGDKSEAVQDLLLLDVTPLSLGIETAGGVMT
ALIKRNTTIPTKQTQTFTTYSDNQPGVLIQVYEGERAMTKDNNLLGKFELSGIPPAPRGV
PQIEVTFDIDANGILNVSAVDKSTGKENKITITNDKGRLSKEEIERMVQDAEKYKADDEK
QRDRISAKNSLESYCFNMKSTVEDEKFKDKISEEDRTKILEMCNEAIKWLDGNQLGEKEE
YEHKQKEIEQVCNPIITKMYGAAGG-PP---------PGGMPGGM---------------
--GGAAPGGAGT---GGS---------SGPTIEEVD

```
>Metapenaeus_ensis_KJ511266
-MSKASAVGIDLGTTYSCVGVFQHGKVEIIANDQGNRTTPSYVAFTDTERLIGDAAKNQV
AMNPNNTVFDAKRLIGRKFDDATVQSDMKHWPFTIVNESTKPKIQVEYKGDKKTFYPEEI
SSMVLIKMKETAEAYLGATVKDAVVTVPAYFNDSQRQATKDAGTISGLNVLRIINEPTAA
AIAYGLDKK--V--G--GERNVLIFDLGGGT-FDVPILTIEDG-IFEVKSTAGDTHLGGE
DFDNRMVNHFIQEFKRKYKKDPSENKRSLRRLRTACERAKRTLSSSTQASVEIDSLFEGI
DFYTSITRARFEELCADLFRGTLEPVEKSLRDAKMDKAQIHDIVLVGGSTRIPKIQKLLQ
DFFNGKELNKSINPDEAVAYGAAVQAAILCGDKSEAVQDLLLLDVTPLSLGIETAGGVMT
ALIKRNTTIPTKQTQTFTTYSDNQPGVLIQVYEGERAMTKDNNLLGKFELSGIPPAPRGV
PQIEVTFDIDANGILNVSAVDKSTGKENKITITNDKGRLSKEEIERMVQDAEKYKADDEK
QRDRISAKNSLESYCFNMKSTVEDEKFKDKISEEDRTKILEMCNEAIKWLDGNQLGEKEE
YEHKQKEIEQVCNPIITKMYGAAGG-PP---------PGGMPGGM---------------
--GGAAPGGAGT---GGS---------SGPTIEEVD
>Portunus_trituberculatus_FJ830635
-MAIARAVGIDLGTTYSCVGVFQHGKVEIITNDQGNRTTPSYVAFTDTERLIGDAAKNQV
AMNPNNTVFDAKRLIGRKFEDATVQSDMKQWPFTVISDGGKPKISVEYKGEAKKFFPEEI
SSMVLIKMKETAEAYLGTTVKDAVVTVPAYFNDSQRQATKDAGTISGMNVLRIINEPTAA
AIAYGLDKR--V--G--GERNVLIFDLGGGT-FDVSILTIEDG-IFEVKSTAGDTHLGGE
DFDNRMVNFFIQEFKRKYKKDLSTNKRAIRRLRTACERAKRTLSSSTQASIEIDSLFEGI
DFYTSVTRARFEELCSDLFRGTLEPVEKSLRDAKLDKAQIHDIVLVGGSTRIPKIQKLLQ
DFFNGKELNKSINPDEAVAYGAAVQAAILCGDKSEEVQDLLLLDVTPLSLGIETAGGVMT
ALIKCNTTIPTKQTQTFTTYSDNQPGVLIQVYEGERAMTRDNNLLGKFELTGIPPAPRGV
PQIEVTFDIDANGILNVSAVDKSTGKENKITITNDKGRLSKEEIERMVNEADKYRAEDEQ
QRERISAKNNLESYCFNMKSTVDDEKFKDKIPESDRTAILDKCNETIQWLDANQLAEKDE
YEHRQKELGKVCNPIITKMYAAAGG-APGGMPGG--MPGGMPGGPQ----G---GS---T
G--------------GGS---------SGPTIEEVD
>Procambarus_clarkii_KU613184
-MPK--AVGIDLGTTFSCVGVFQHGKVEIIANDQGNRTTPSYVAFTDAERLIGDAAKNQV
AMNPNNTVFDAKRLIGRKFNDASVQADMKHWPFTVISDGGKPKIQIEYKGETKSFYPEEI
SSMVLIKMKETAEAYLGSTVKDAVITVPAYFNDSQRQATKDAGTISGMNVLRIINEPTAA
AIAYGLDKK--V--G--GERNVFIFDLGGGT-FDVSILPIEDG-IFEVKSTAGDTHLGGE
DFDNRMVTHFIQEFKRKYKKDMSDNKRAVRRLRTACERAKRTLSSSTQASIEIDSLYEGV
DFYTSITRARFEEMCADLFRGALDPVEKSLRDAKMDKSQINEIVMVGGSTRIPKIQKLLQ
DFFNGKELNKSINPDEAVAYGAAVQAAILCGDKSEAVQDLLLLDVAPLSLGIETAGGVMT
ALIKRNTTIPTKQTQTFTTYSDNQPGVLIQVYEGERAMTKDNNLLGKFELTGIPPAPRGV
PQIEVTFDIDANGILNVSAADKSTGKENKITITNDKGRLSKEEIEKMVQDAEKYKADDEN
QRERIAAKNSLESYCFNMKSTVEDEKFKDKVSSTDRSKILDACNEAIKWLDSNQSAEKDE
FEHKQKEVEQICNPIITKMYASSGG-APG-APGG--APEG---------------A----
-----STG---G---G-------------PTIEEVD
>Pontastacus_leptodactylus_GAFY01000699
-MPK--AVGIDLGTTFSCVGVFQHGKVEIIANDQGNRTTPSYVAFTDAERLIGDAAKNQV
AMNPNNTVFDAKRLIGRKFTDATVQADMKHWPFAVINDGGKPKIQIEYKGEMKSFYPEEI
SSMVLIKMKETAEAYLGSTVKDAVITVPAYFNDSQRQATKDAGTISGMNVLRIINEPTAA
AIAYGLDKK--V--G--GERNVLIFDLGGGT-FDVSILTIEDG-IFEVKSTAGDTHLGGE
DFDNRMVNHFIQEFKRKYKKDLSDNKRAVRRLRTACERAKRTLSSSTQASIEIDSLYEGV
DFYTSITRARFEEMCADLFRGTLDPVEKSLRDAKMDKSQINEIVMVGGSTRIPKIQKLLQ
DFFNGKELNKSINPDEAVAYGAAVQAAILCGDKSEAVQDLLLLDVAPLSLGIETAGGVMT
ALIKRNTTIPTKQTQTFTTYSDNQPGVLIQVYEGERAMTKDNNLLGKFELTGIPPAPRGV
PQIEVTFDIDANGILNVSAADKSTGKENKITITNDKGRLSKEEIEKMVQDAEKYKADDEN
QRERISAKNSLESYCFNMKSTVEDDKFKDKVSSNDRSKILDACNDAIKWLDSNQSAEKDE
FEHKQKEVEQICNPIITKMYASSGG-APGGAPGG--APEG---------------A----
-----STG---G---G-------------PTIEEVD
>Cherax_cainii_KR058823
-MTK--AVGIDLGTTFSCVGVFQHGKVEIIANDQGNRTTPSYVAFTDAERLIGDAAKNQV
AMNPNNTVFDAKRLIGRKFNDSTVQADMKHWPFMVINDGGKPKIQIEYKGEMKSFYPEEI
SSMVLIKMKETAEAYLGATVKDAVITVPAYFNDSQRQATKDAGTISGMNVLRIINEPTAA
AIAYGLDKK--V--G--GERNVLIFDLGGGT-FDVSILTIEDG-IFEVKSTAGDTHLGGE
DFDNRMVTHFIQEFKRKYKKDLSDNKRAVRRLRTACERAKRTLSSSTQASVEIDSLYEGV
DFYTSITRARFEEMCADLFRGTLDPVEKALRDAKMDKSQINDIVMVGGSTRIPKIQKLLQ
DFFNGKELNKSINPDEAVAYGAAVQAAILSGDKSEAVQDLLLLDVAPLSLGIETAGGVMT
ALIKRNTTIPTKQTQTFTTYSDNQPGVLIQVYEGERAMTKDNNLLGKFELTGIPPAPRGV
PQIEVTFDIDANGILNVSAVDKSTGKENKITITNDKGRLSKEEIEKMVQDAEKYKADDEK
QRERISAKNSLESYCFNMKSTVEDDKFKDKVSGEDCSKILDACNDAIKWLDTNQLAEKEE
YEHKQKEVEQICNPIITKMYGASGG-APGGMPGG--MPGG---------------A----
-----APG---G---GSA---------GGPKIEEVD
>Cherax_quadricarinatus_KR058821
-MTK--AVGIDLGTTFSCVGVFQHGKVEIIANDQGNRTTPSYVAFTDAERLIGDAAKNQV
AMNPNNTVFDAKRLIGRKFNDSTVQADMKHWPFMVINDGGKPKIQIEYKGEMKSFYPEEI
SSMVLIKMKETAEAYLGATVKDAVITVPAYFNDSQRQATKDAGTISGMNVLRIINEPTAA
AIAYGLDKK--V--G--GERNVLIFDLGGGT-FDVSILTIEDG-IFEVKSTAGDTHLGGE
DFDNRMVTHFIQEFKRKYKKDLSDNKRAVRRLRTACERAKRTLSSSTQASVEIDSLYEGV
```

```
DFYTAITRARFEEMCADLFRGTLDPVEKALRDAKMDKSQINDIVMVGGSTRIPKIQKLLQ
DFFNGKELNKSINPDEAVAYGAAVQAAILSGDKSEAVQDLLLLDVAPLSLGIETAGGVMT
ALIKRNTTIPTKQTQTFTTYSDNQPGVLIQVYEGERAMTKDNNLLGKFELTGIPPAPRGV
PQIEVTFDIDANGILNVSAVDKSTGKENKITITNDKGRLSKEEIEKMVQDAEKYKADDEK
QRERISAKNSLESYCFNMKSTVEDDKFKDKVSGEDCSKILDACNDAIKWLDTNQLAEKEE
YEHKQKEVEQICNPIITKMYGASGG-APGGMPGG--MPGG---------------A----
-----APG---G---GSA---------GGPKIEEVD
>Cherax_quadricarinatus_HM800921
-MTKAPAVGIDLGTTFSCVGVFQHGKVEIIANDQGNRTTPSYVAFTDAERLIGDAAKNQV
AMNPNNTVFDAKRLIGRKFNDSTVQADMKHWPFMVINDGGKPKIQIEYKGEMKSFYPEEI
SSMVLIKMKETAEAYLGATVKDAVITVPAYFNDSQRQATKDAGTISGMNVLRIINEPTAA
AIAYGLDKK--V--G--GERNVLIFDLGGGTTFDVSILTIEDG-IFEVKSTAGDTHLGGE
DFDNRMVTHFIQEFKRKYKKDLSDNKRAVRRLRTACERAKRTLSSSTQASVEIDSLYEGV
DFYTAITRARFEEMCADLFRGTLDPVEKALRDAKMDKSQINDIVMVGGSTRIPKIQKLLQ
DFFNGKELNKSINPDEAVAYGAAVQAAILSGDKSEAVQDLLLLDVAPLSLGIETAGGVMT
ALIKRNTTIPTKQTQTFTTYSDNQPGVLIQVYEGERAMTKDNNLLGKFELTGIPPAPRGV
PQIEVTFDIDANGILNVSAVDKSTGKENKITITNDKGRLSKEEIEKMVQDAEKYKADDEK
QRERISAKNSLESYCFNMKSTVEDDKFKDKVSGEDCSKILDACNDAIKWLDTNQLAEKEE
YEHKQKEVEQICNPIITKMYGASGG-APGGMPGG--MPGG---------------A---P
GAGGAAPG---G---GSA---------GGPTIEEVD
>Eulimnogammarus_verrucosus_JQ003919
-MSKATAVGIDLGTTYSCVGVFQHGKVEIIANDQGNRTTPSYVAFTDTERLIGDAAKNQV
AMNPTNTVFDAKRLIGRKFDEATVQSDMKHWPFEVVNENTKPKISVDYKGEKKTFTPEEI
SSMVLTKMKETADAYLGTNIKDAVVTVPAYFNDSQRQATKDAGTISGLNVLRIINEPTAA
AIAYGLDKK--V--G--GERNVLIFDLGGGT-FDVSILTIEDG-IFEVKSTAGDTHLGGE
DFDNRMVNHFMQEFKRKYKKDLSENKRSLRRLRTACERAKRTLSSSTQASIEIDSLYDGI
DFYTSVTRARFEELCADLFRGTLDPVEKSLRDAKLDKGQIQEIVLVGGSTRIPKIQKLLQ
DFFNGKELNKSINPDEAVAYGAAVQAAILSGDKSEAVQDLLLLDVAPLSMGIETAGGVMT
ALIKRNTTIPTKQTQTFTTYSDNQPGVLIQVYEGERAMTKDNNLLGKFELTGIPPAPRGV
PQIEVTFDIDANGILNVSAVDKSTGKENKITITNDKGRLSKEEIERMVQDAEKYKNDDDN
QRERISAKNGLESYCFNMKSTVEDDKVKDKISEDDRKKIMEACDEAIKWLDGNQLAEKEE
YEHKQKEVEKVCTPIITKLY---GG-AGAAPP-----PGA---------------A---P
GT-GAAPG---A---GGA---------GGPTIEEVD
>Eulimnogammarus_verrucosus_JN704341
-MSKATAVGIDLGTTYSCVGVFQHGKVEIIANDQGNRTTPSYVAFTDTERLIGDAAKNQV
AMNPTNTVFDAKRLIGRKFDEATVQSDMKHWPFEVVNENTKPKISVDYKGEKKTFTPEEI
SSMVLTKMKETADAYLGTNIKDAVVTVPAYFNDSQRQATKDAGTISGLNVLRIINEPTAA
AIAYGLDKK--V--G--GERNVLIFDLGGGT-FDVSILTIEDG-IFEVKSTAGDTHLGGE
DFDNRMVNHFTQEFKRKYKKDLAENKRSLRRLRTACERAKRTLSSSTQASIEIDSLYDGI
DFYTSVTRARFEELCADLFRGTLDPVEKSLRDAKLDKGQIQEIVLVGGSTRIPKIQKLLQ
DFFNGKELNKSINPDEAVAYGAAVQAAILSGDKSEAVQDLLLLDVAPLSMGIETAGGVMT
ALIKRNTTIPTKQTQTFTTYSDNQPGVLIQVYEGERAMTKDNNLLGKFELTGIPPAPRGV
PQIEVTFDIDANGILNVSAVDKSTGKENKITITNDKGRLSKEEIERMVQDAEKYKNDDDN
QRERISAKNGLESYCFNMKSTVEDDKVKDKISEDDRKKIMEACDEAIKWLDGNQLAEKEE
YEHKQKEVEKVCTPIITKLY---GG-AGAAPP-----PGA---------------A---P
GT-GAAPG---A---GGA---------GGPTIEEVD
>Eulimnogammarus_cyaneus_JN704343
-MSKATAVGIDLGTTYSCVGVFQHGKVEIIANDQGNRTTPSYVAFTDTERLIGDAAKNQV
AMNPTNTVFDAKRLIGRKFDEATVQSDMKHWPFEVVNENTKPKISVDYKGEKKTFTPEEI
SSMVLTKMKETADAYLGTNIKDAVVTVPAYFNDSQRQATKDAGTISGLNVLRIINEPTAA
AIAYGLDKK--V--G--GERNVLIFDLGGGT-FDVSILTIEDG-IFEVKSTAGDTHLGGE
DFDNRMVNHFTQEFKRKYKKDLAENKRSLRRLRTACERAKRTLSSSTQASIEIDSLYDGI
DFYTSVTRARFEELCADLFRGTLDPVEKSLRDAKLDKGQIQEIVLVGGSTRIPKIQKLLQ
DFFNGKELNKSINPDEAVAYGAAVQAAILSGDKSEAVQDLLLLDVAPLSMGIETAGGVMT
ALIKRNTTIPTKQTQTFTTYSDNQPGVLIQVYEGERAMTKDNNLLGKFELTGIPPAPRGV
PQIEVTFDIDANGILNVSAVDKSTGKENKITITNDKGRLSKEEIERMVQDAEKYKNDDDN
QRERISAKNGLESYCFNMKSTVEDDKVKDKISEDDRKKIMEACDEAIKWLDGNQLAEKEE
YEHKQKEVEKVCTPIITKLY---GG-AGAAPP-----PGA---------------A---P
GT-GAAPG---A---GGA---------GGPTIEEVD
>Gammarus_lacustris_JN704340
-MSKATAVGIDLGTTYSCVGVFQHGKVEIIANDQGNRTTPSYVAFTDTERLIGDAAKNQV
AMNPTNTVFDAKRLIGRKFDEATVQSDMKHWPFEVVNENTKPKISVDYKGEKKTFTPEEI
SSMVLTKMKETADAYLGTNIKDAVVTVPAYFNDSQRQATKDAGTISGLNVLRIINEPTAA
AIAYGLDKK--V--G--GERNVLIFDLGGGT-FDVSILTIEDG-IFEVKSTAGDTHLGGE
DFDNRMVNHFTQEFKRKYKKDLAENKRSLRRLRTACERAKRTLSSSTQASIEIDSLYDGI
DFYTSVTRARFEELCADLFRGTLDPVEKSLRDAKLDKGQIQEIVLVGGSTRIPKIQKLLQ
DFFNGKELNKSINPDEAVAYGAAVQAAILSGDKSEAVQDLLLLDVAPLSMGIETAGGVMT
ALIKRNTTIPTKQTQTFTTYSDNQPGVLIQVYEGERAMTKDNNLLGKFELTGIPPAPRGV
PQIEVTFDIDANGILNVSAVDKSTGKENKITITNDKGRLSKEEIERMVQDAEKYKNDDDN
QRERISAKNGLESYCFNMKSTVEDDKVKDKISEDDRKKIMEACDEAIKWLDGNQLAEKEE
YEHKQKEVEKVCTPIITKLY---GG-AGAAPP-----PGA---------------A---P
```

```
GT-GAAPG---A---GGA---------GGPTIEEVD
>Echinogammarus_veneris_GARO01000312
-MSKATAVGIDLGTTYSCVGVFQHGKVEIIANDQGNRTTPSYVAFTDTERLIGDAAKNQV
AMNPTNTVFDAKRLIGRKFDDSVVQADMKHWPFEVVSENSKPKISVDYRGEKKTFTPEEI
SSMVLTKMKETADAYLGTNIKDAVVTVPAYFNDSQRQATKDAGTISGLNVLRIINEPTAA
AIAYGLDKK--V--G--GERNVLIFDLGGGT-FDVSILTIEDG-IFEVKSTAGDTHLGGE
DFDNRMVSHFMHEFKRKYKKDLTENKRSLRRLRTACERAKRTLSSSTQASIEIDSLYDGI
DFYTSITRARFEELCADLFRGTLDPVEKSLRDAKLDKGQIQEIVLVGGSTRIPKIQKLLQ
DFFNGKELNKSINPDEAVAYGAAVQAAILSGDKSEAVQDLLLLDVAPLSMGIETAGGVMT
ALIKRNTTIPTKQTQTFTTYSDNQPGVLIQVYEGERAMTKDNNLLGKFELTGIPPAPRGV
PQIEVTFDIDANGILNVSAVDKSTGKENKITITNDKGRLSKEEIERMVQDAEKYKNDDDQ
QRERISAKNGLESYCFNMKSTVEDDKVKDKISEEDRKKIMEACDEAIKWLDGNQLAEKEE
YEHKQKEVEKVCTPIITKLY---GG-AGGAPP-----PGA---------------A---P
GAAGAAPG---A---GGA---------GGPTIEEVD
>Hyalella_azteca_JQDR01082346
-MSKASAVGIDLGTTYSCVGVFQHGKVEIIANDQGNRTTPSYVAFTDTERLIGDAAKNQV
AMNPTNTVFDAKRLIGRKFDEPTVQSDMKHWPFTVVNDGSKPKISVEYKGETKTFTPEEI
SSMVLTKMKETADAYLGVNIKDAVITVPAYFNDSQRQATKDAGTISGLNVLRIINEPTAA
AIAYGLDKK--V--G--GERNVLIFDLGGGT-FDVSILTIEDG-IFEVKSTAGDTHLGGE
DFDNRMVNHFMQEFKRKYKKDLAENKRSLRRLRTACERAKRTLSSSAQASIEIDSLFDGI
DFYTSVTRARFEELCADLFRGTLDPVEKSLRDAKLDKGQIQEIVLVGGSTRIPKIQKLLQ
DFFNGKELNKSINPDEAVAYGAAVQAAILSGDKSEAVQDLLLLDVAPLSMGIETAGGVMT
ALIKRNTTIPTKQTQTFTTYSDNQPGVLIQVYEGERAMTKDNNLLGKFELTGIPPAPRGV
PQIEVTFDIDANGILNVSAVDKSTGKENKITITNDKGRLSKEEIERMVQDAEKYKNDDDN
QRERISAKNGLESYCFNMKSTVEDDKVKDKISEEDRKKIMEACDEAIKWLDGNQLAEKEE
YEHKQKDIEKVCTPIITKLY---GG-AAGGMP-----PGA---------------A----
GA-GAPPP---G---AGA---------GGPTIEEVD
>Hyalella_azteca_JQDR01082349
-MVKTSAVGIDLGTTYSCVGVFQHGKVEIIANDQGNRTTPSYVAFTDTERLIGDAAKNQV
ALNPVNTIFDAKRLIGRRFDDPTVAGDMKHWPFKVVNDSGKPKLQVEFKGEMKTFSPEEI
SSMVLTKMKEIAEAYLGGAVKDAVITVPAYFNDSQRQATKDAGAIAGLNVLRIINEPTAA
AIAYGLDKK--G--TGAGERNILIFDLGGGT-FDVSILSIDDG-IFEVKATAGDTHLGGE
DFDNRLVNHFVEEFKRKYKKDIKDNKRTLRRLRTACERAKRTLSSSAQASIEIDSLYEGI
DFYTSITRARFEEMCSDLFRGTLDPVEKSLRDAKMDKGHIHEIVLVGGSTRIPKIQKLLQ
DFFNGKELNKSINPDEAVAYGAAVQAAILTGDKSEAVQDLLLLDVAPLSMGIETAGGVMT
ALIKRNTTIPTKHSQIFTTYSDNQPGVLIQVYEGERAMTKDNNLLGKFELTGIPPAPRGV
PQIEVTFDIDANGILNVSAVDKSTGKANKITITNDKGRLSKEDIEKMVQDAEKYKAEDDK
QKEKIAAKNSLESYCFNMKSTLEDDKLKDKVPEEDRKKALDACSEAIKWLDANQLADKEE
FEFKQKEVEKICSPVITKLY---GG--AGGMPGG--MPGG--------------M---P
GAA------------SGA---------GGPTIEEVD
>Hyalella_azteca_JQDR01082354
-MVKGASVGIDLGTTYSCVGVFQHGNVEIITNDQGNRTTPSYVAFTDSERLIGDAAKNQV
AFNPDNTIFDAKRLIGRRFEDPIVAGDMLFWPFKVVNDSSKPKFQVEFKGEMKTFSPEEI
SSMVLTKMKEIAEAYLGGAVKDAVITVPAYFNDSQRQATKDAGAIAGLNVLRIINEPTAA
AIAYGLDKK--G--TGAGERNILIFDLGGGT-FDVSILSIDDG-IFEVKATAGNTHLGGE
DFDILLVIHFVEEFKRKYKKDIRDNKRSLRRLMTACERAKITLSSSAQASIEIDSLYEGI
DFYTSISRARFEEMCSDLFRGTLVPVEKSLRDAKMDKGHIHDIVLVGGSTRIPKIQKLLQ
DFFNGKKLNKSINPDEAVAYGAAVQAAILTGDKSEAVQDLLLLDVAPLSTGIETAGGVMT
ALIKRNTAIPTKHSQNFSTYWDNQPGVLIQVYEGERAMTKDNNLLGEFELFGIPPAPRGV
PQVQVTFNIDANGILDVSAVDNSTGKENNITITNDKGRLSKEEIEKMVLDAEKYKAEDDK
QKEKVAAKNSLESYCFNMKSTLEDDKLKDKVSEEERKKALDACSEAIQWIYANQLAEKEE
FEFKQNEIAKICSPLISKLY----------------------------------------
------------------------------------
>Hyalella_azteca_JQDR01033640
-MVKIPAVGIDLGTTYSCVAVFQRGKVEIIANDQGNRTTPSYVAFTDTERLIGDAAKNQV
ALNPVNTIFDAKRLIGRKFDDPTVAGDMKHWPFKVINHSDKPKLQVEFKREMKTFSPEEI
SSMVLTKMKEIAEAYLGGDVKDAVISVPAYFNDSQRQATKDAGAIAGLNVLRIINEPAAA
AIAYGLDKK--G--TGAGERNILIFDLGGGT-VGVSILSIDDG-IFEVKATAGDNHLGGE
DFDNRIVNHFVQEFKRKYKRDIEDNKRALQRLRTACERAKRTLSSSAQASIELDSLYEGI
NFYTSITRARFEELCADLFRGTLDLVEKSLRDAKMEQGHIHEIVLVGSSTRIPKIQKLLQ
DFFYGKELNKSINPDEAVAYGAAVLAAILSGDKSEAVKDVLLLDVAPLSMGIETAGGVMT
ALIKRNTIIPSIRSQIFTTYSDNQPGVLIQVYEGERAMTKDNNLLGMFELIDIPPAPRGV
PQIEVSFDINVNGILNVSAVDMSTGKKIKITIANDKSRLSKEEIEKMVQDAEKYKEEDEK
QKEKIAAKNSLESYCFNMKSTLEDDKLKDKVPEEELKKALGACSKAITWLDANQHAEKKE
FENTQKQVENICSYVISKLY---RG--ASAMPGG--MP----------------------
GAA------------FGA---------GGPLKERVD
>Ommatogammarus_flavus_GEQS00000000.1
MRAKSTAVGIDLGTTYSCVGVFQHGKVEIIANDQGNRTTPSYVAFTDTERLIGDAAKNQV
ALNPTNTVFDAKRLIGRKFGDSFVNKDMEHWPFKVVNDGGKPMIGVEYKGESKTFSPEEI
SSMVLTKMREISEAYLGKEVKDAVITVPAYFNDSQRQATKDAGAIAGLNVLRIINEPTAA
AIAYGLDKK--G--K--GEKNILIFDLGGGT-FDVSILTIDDG-IFEVKATAGDTHLGGE
```

DFDNRLVDHFAQEFKRKFKKDITQNKRALRRLRTACERAKRTLSSSAQASIEIDSLYEGT
DFYTSITRARFEEMCSDLFRGTLEPVEKSLRDAKMDKGQIHEIVLVGGSTRIPKIQKLLQ
DFFNGKELNKTINPDEAVAYGAAVQAAILTGDNSEAVRDLLLLDVAPLSMGIETAGGVMT
ALIKRNTTIPTKTSQVFSTYADNQPGVLIQVFEGERAMTRDNNLLGKFELSGLPPAPRGV
PQIEVTFDIDANGILNVSAVDKSTGKENKITITNDKGRLSKEDIERLVQDAEKYKANDDQ
QREKIAAKNGLESYCFNMKSAVEDDKLKDKISESDRSKVLEACNEVLKWLDANQLGEKDE
YEHKQKEVEKVCSPIISKLY---GGGAAGCSPGAGPMP----------------------
GAA------------NGA---------G-PTIEEVD
>Carinurus_bicarinatus_GEPL00000000.1
MRAKSTAVGIDLGTTYSCVGVFQHGKVEIIANDQGNRTTPSYVAFTDTERLIGDAAKNQV
ALNPTNTVFDAKRLIGRKFGDSFVNKDMEHWPFKVVNDGGKPMIGVEYKGESKTFSPEEI
SSMVLTKMREISEAYLGKEVKDAVITVPAYFNDSQRQATKDAGAIAGLNVLRIINEPTAA
AIAYGLDKK--G--K--GEKNILIFDLGGGT-FDVSILTIDDG-IFEVKATAGDTHLGGE
DFDNRLVDHFAQEFKRKYKKDITQNKRALRRLRTACERAKRTLSSSAQANIEIDSLYEGT
DFYTSITRARFEEMCSDLFRGTLDPVEKSLRDAKMDKGQIHEIVLVGGSTRIPKIQKLLQ
DFFNGKELNKTINPDEAVAYGAAVQAAILTGDNSEAVRDLLLLDVAPLSMGIETAGGVMT
ALIKRNTTIPTKTSQVFSTYADNQPGVLIQVFEGERAMTRDNNLLGKFELSGLPPAPRGV
PQIEVTFDIDANGILNVSAVDKSTGKENKITITNDKGRLSKEDIERLVQDAEKYKADDDL
QREKIAAKNGLESYCFNMKSAVEDDKVKDKISESDRNKVLEACNEVLKWLDANQLGEKDE
YEHKQKEVEKVCSPIISKLY---GGGAAGCSPGAGPMP----------------------
GAA------------NGA---------G-ATIEEVD
>Hyalellopsis_setosa_GEQI00000000.1
MRAKSTAVGIDLGTTYSCVGVFQHGKVEIIANDQGNRTTPSYVAFTDTERLIGDAAKNQV
ALNPTNTVFDAKRLIGRKFGDSSINKDMEHWPFKVVNDGGKPMIGVEYKGESKTFSPEEI
SSMVLTKMREISEAYLGKEVKDAVITVPAYFNDSQRQATKDAGAIAGLNVLRIINEPTAA
AIAYGLDKK--G--K--GEKNILIFDLGGGT-FDVSILTIDDG-IFEVKATAGDTHLGGE
DFDNRLVDHFAQEFKRKFKKDITQNKRALRRLRTACERAKRTLSSSAQASIEIDSLHEGT
DFYTSITRARFEEMCSDLFRGTLDPVEKSLRDAKMDKGQIHEIVLVGGSTRIPKVQKLLQ
DFFNGKELNKTINPDEAVAYGAAVQAAILTGDNSEAVRDLLLLDVAPLSMGIETAGGVMT
ALIKRNTTIPTKTSQVFSTYADNQPGVLIQVFEGERAMTRDNNLLGKFELSGLPPAPRGV
PQIEVTFDIDANGILNVSAVDKSTGKENKITITNDKGRLSKEDIERLVQDAEKYKSDDDL
QREKIAAKNGLESYCFNMKSTVEDDKLKDKISESDRSKVLEVCNEVLKWLDANQLGEKDE
YEHKQKEIEKVCSPIISKLY---GGGAAGCSPGAGPMP----------------------
GAA------------NGA---------G-PTIEEVD
>Eulimnogammarus_verrucosus_Eve10LT24_1
MRAKSTAVGIDLGTTYSCVGVFQHGKVEIIANDQGNRTTPSYVAFTDTERLIGDAAKNQV
ALNPTNTVFDAKRLIGRKFGDSSVNKDMEHWPFKVVNDGGKPMIGVEFKGESKTFSPEEI
SSMVLTKMREISEAYLGKEVKDAVITVPAYFNDSQRQATKDAGAIAGLNVLRIINEPTAA
AIAYGLDKK--G--K--GEKNILIFDLGGGT-FDVSILTIDDG-IFEVKATAGDTHLGGE
DFDNRLVDHFAQEFKRKYKKDITQNKRALRRLRTACERAKRTLSSSAQASIEIDSLYEGT
DFYTSITRARFEEMCSDLFRGTLDPVEKSLRDAKMDKGQIHEIVLVGGSTRIPKIQKLLQ
DFFNGKELNKTINPDEAVAYGAAVQAAILTGDNSEAVRDLLLLDVAPLSMGIETAGGVMT
ALIKRNTTIPTKTSQVFSTYADNQPGVLIQVFEGERAMTRDNNLLGKFELSGLPPAPRGV
PQIEVTFDIDANGILNVSAVDKSTGKENKITITNDKGRLSKEDIERLVQDAEKYKADDDL
QREKIAAKNGLESYCFNMKSTVEDDKLKDKISESDRSKVLEACNEVLKWLDANQLGEKDE
YEHKQKEVEKVCSPIISKLY---GGGAAGCSPGAGPMP----------------------
GAA------------NGA---------G-PTIEEVD
>Eulimnogammarus_verrucosus_Eve10LT24_2
MRAKGTAVGIDLGTTYSCVGVFQHGKVEIIANDQGNRTTPSYVAFTDTERLIGDAAKNQV
ALNPTNTVFDAKRLIGRKFGDSSVNKDMEHWPFKVVNDGGKPMIGVEFKGESKTFSPEEI
SSMVLTKMREISEAYLGKEVKDAVITVPAYFNDSQRQATKDAGAIAGLNVLRIINEPTAA
AIAYGLDKK--G--K--GEKNILIFDLGGGT-FDVSILTIDDG-IFEVKATAGDTHLGGE
DFDNRLVDHFAQEFKRKFKKDITQNKRALRRLRTACERAKRTLSSSAQASIEIDSLYEGT
DFYTSITRARFEEMCSDLFRGTLDPVEKSLRDAKMDKGQIHEIVLVGGSTRIPKIQKLLQ
DFFNGKELNKTINPDEAVAYGAAVQAAILTGDNSEAVRDLLLLDVAPLSMGIETAGGVMT
ALIKRNTTIPTKTSQVFSTYADNQPGVLIQVFEGERAMTRDNNLLGKFELSGLPPAPRGV
PQIEVTFDIDANGILNVSAVDKSTGKENKITITNDKGRLSKEDIERLVQDAEKYKADDDL
QREKIAAKNGLESYCFNMKSTVEDDKLKDKISESDRSKVLEACNEVLKWLDANQLGEKDE
YEHKQKEVEKVCSPIISKLY---GGGAAGCSPGAGPMP----------------------
GAA------------NGT---------G-PTIEEVD
>Eulimnogammarus_verrucosus_Eve10LT24_3
MRAKSTAVGIDLGTTYSCVGVFQHGKVEIIANDQGNRTTPSYVAFTDTERLIGDAAKNQV
ALNPTNTVFDAKRLIGRKFGDSSVNKDMEHWPFKVVNDGGKPMIGVEFKGESKTFSPEEI
SSMVLTKMREISEAYLGKEVKDAVITVPAYFNDSQRQATKDAGAIAGLNVLRIINEPTAA
AIAYGLDKK--G--K--GEKNILIFDLGGGT-FDVSILTIDDG-IFEVKATAGDTHLGGE
DFDNRLVDHFAQEFKRKFKKDITQNKRALRRLRTACERAKRTLSSSAQASIEIDSLYEGT
DFYTSITRARFEEMCSDLFRGTLDPVEKSLRDAKMDKGQIHEIVLVGGSTRIPKIQKLLQ
DFFNGKELNKTINPDEAVAYGAAVQAAILTGDNSEAVRDLLLLDVAPLSMGIETAGGVMT
ALIKRNTTIPTKTSQVFSTYADNQPGVLIQVFEGERAMTRDNNLLGKFELSGLPPAPRGV
PQIEVTFDIDANGILNVSAVDKSTGKENKITITNDKGRLSKEDIERLVQDAEKYKADDDL
QREKIAAKNGLESYCFNMKSTVEDDKLKDKISESDRSKVLEACNEVLKWLDANQLGEKDE

YEHKQKEVEKVCSPIISKLY---GGGAAGCSPGAGPMP---------------------
GAA------------NGT---------G-PTIEEVD
>Eulimnogammarus_cyaneus_Ecy10LT24_1
MRAKGTAVGIDLGTTYSCVGVFQHGKVEIIANDQGNRTTPSYVAFTDTERLIGDAAKNQV
ALNPTNTVFDAKRLIGRKFGDSSVNKDMEHWPFKVVNDGGKPMIGVEFKGESKTFSPEEI
SSMVLTKMREISEAYLGKEVKDAVITVPAYFNDSQRQATKDAGAIAGLNVLRIINEPTAA
AIAYGLDKK--G--K--GEKNILIFDLGGGT-FDVSILTIDDG-IFEVKATAGDTHLGGE
DFDNRLVDHFAQEFKRKYKKDITQNKRALRRLRTACERAKRTLSSSAQASIEIDSLYEGT
DFYTSITRARFEEMCSDLFRGTLEPVEKSLRDAKMDKGQIHEIVLVGGSTRIPKIQKLLQ
DFFNGKELNKSINPDEAVAYGAAVQAAILTGDNSEAVRDLLLLDVAPLSMGIETAGGVMT
ALIKRNTTIPTKTSQVFSTYADNQPGVLIQVFEGERAMTRDNNLLGKFELSGLPPAPRGV
PQIEVTFDIDANGILNVSAVDKSTGKENKITITNDKGRLSKEDIERLVQDAEKYKADDDL
QREKIAAKNGLESYCFNMKSTVEDDKVKDKISESDRSKVLEACNEVLKWLDANQLGEKDE
YEHKQKEVEKVCSPIISKLY---GGGAAGCSPGAGPMP---------------------
GAA------------NGT---------G-PTIEEVD
>Eulimnogammarus_cyaneus_Ecy10LT24_2
MRAKSTAVGIDLGTTYSCVGVFQHGKVEIIANDQGNRTTPSYVAFTDTERLIGDAAKNQV
ALNPTNTVFDAKRLIGRKFGDSSVNKDMEHWPFKVVNDGGKPMIGVEFKGESKTFSPEEI
SSMVLTKMREISEAYLGKEVKDAVITVPAYFNDSQRQATKDAGAIAGLNVLRIINEPTAA
AIAYGLDKK--G--K--GEKNILIFDLGGGT-FDVSILTIDDG-IFEVKATAGDTHLGGE
DFDNRLVDHFAQEFKRKYKKDITQNKRALRRLRTACERAKRTLSSSAQASIEIDSLYEGT
DFYTSITRARFEEMCSDLFRGTLEPVEKSLRDAKMDKGQIHEIVLVGGSTRIPKIQKLLQ
DFFNGKELNKSINPDEAVAYGAAVQAAILTGDNSEAVRDLLLLDVAPLSMGIETAGGVMT
ALIKRNTTIPTKTSQVFSTYADNQPGVLIQVFEGERAMTRDNNLLGKFELSGLPPAPRGV
PQIEVTFDIDANGILNVSAVDKSTGKENKITITNDKGRLSKEDIERLVQDAEKYKADDDL
QREKIAAKNGLESYCFNMKSTVEDDKVKDKISESDRSKVLEACNEVLKWLDANQLGEKDE
YEHKQKEVEKVCSPIISKLY---GGGAAGCSPGAGPMP---------------------
GAA------------NGT---------G-PTIEEVD
>Eulimnogammarus_cyaneus_Ecy10LT24_3
MRAKSTAVGIDLGTTYSCVGVFQHGKVEIIANDQGNRTTPSYVAFTDTERLIGDAAKNQV
ALNPTNTVFDAKRLIGRKFGDSTVNKDMEHWPFKVVNDGGKPMIGVEFKGESKTFSPEEI
SSMVLTKMREISEAYLGKEVKDAVITVPAYFNDSQRQATKDAGAIAGLNVRRIINEPTAA
AIAYGLDKK--G--K--GEKNILIFDLGGGT-FDVSILTIDDG-IFEVKATAGDTHLGGE
DFDNRLVDHFAQEFKRKYKKDITQNKRALRRLRTACERAKRTLSSSAQASIEIDSLYEGT
DFYTSITRARFEEMCSDLFRGTLEPVEKSLRDAKMDKGQIHEIVLVGGSTRIPKIQKLLQ
DFFNGKELNKSINPDEAVAYGAAVQAAILTGDNSEAVRDLLLLDVAPLSMGIETAGGVMT
TLIKRNTTIPTKTSQVFSTYADNQPGVLIQVFEGERAMTRDNNLLGKFELSGLPPAPRGV
PQIEVTFDIDANGILNVSAVDKSTGKENKITITNDKGRLSKEDIERLVQDAEKYKADDDL
QREKIAAKNGLESYCFNMKSTVEDDKVKDKISESDRSKVLEACNEVLKWLDANQLGEKDE
YEHKQKEVEKVCSPIISKLY---GGGAAGCSPGAGPMP---------------------
GAA------------NGT---------G-PTIEEVD
>Eulimnogammarus_cyaneus_Ecy10LT24_4
MRAKSTAVGIDLGTTYSCVGVFQHGKVEIIANDQGNRTTPSYVAFTDTERLIGDAAKNQV
ALNPTNTVFDAKRLIGRKFGDSSVNKDMEHWPFKVVNDGGKPMIGVEFKGESKTFSPEEI
SSMVLTKMREISEAYLGKEVKDAVITVPAYFNDSQRQATKDAGAIAGLNVLRIINEPTAA
AIAYGLDKK--G--K--GEKNILIFDLGGGT-FDVSILTIDDG-IFEVKATAGDTHLGGE
DFDNRLVDHFAQEFKRKYKKDITQNKRALRRLRTACERAKRTLSSSAQASIEIDSLYEGT
DFYTSITRARFEEMCSDLFRGTLEPVEKSLRDAKMDKGQIHEIVLVGGSTRIPKIQKLLQ
DFFNGKELNKSINPDEAVAYGAAVQAAILTGDNSEAVRDLLLLDVAPLSMGIETAGGVMT
TLIKRNTTIPTKTSQVFSTYADNQPGVLIQVFEGERAMTRDNNLLGKFELSGLPPAPRGV
PQIEVTFDIDANGILNVSAVDKSTGKENKITITNDKGRLSKEDIERLVQDAEKYKADDDL
QREKIAAKNGLESYCFNMKSTVEDDKVKDKISESDRSKVLEACNEVLKWLDANQLGEKDE
YEHKQKEVEKVCSPIISKLY---GGGAAGCSPGAGPMP---------------------
GAA------------NGT---------G-PTIEEVD
>Oxyacanthus_flavus_GEPB00000000.1
MRAKSTAVGIDLGTTYSCVGVFQHGKVEIIANDQGNRTTPSYVAFTDTERLIGDAAKNQV
ALNPTNTVFDAKRLIGRKFGDSCVNKDMKHWPFKVVNDGGKPMIGVEYKGESKTFSPEEI
SSMVLTKMREISEAYLGKEVKDAVITVPAYFNDSQRQATKDAGAIAGLNVLRIINEPTAA
AIAYGLDKI--E--S--GEKNILIFDLGGGT-FDVSILTIDDG-IFEVKATAGDTHLGGE
DFDNRLVDHFAQEFKRKYKKDITQNKRALRRLRTACERAKRTLSSSAQASIEIDSLYEGT
DFYTSITRARFEEMCSDLFRGTLEPVEKSLRDAKMDKGQIHEIVLVGGSTRIPKIQKLLQ
DFFNGKELNKTINPDEAVAYGAAVQAAILTGDNSEAVRDLLLLDVAPLSMGIETAGGVMT
ALIKRNTTIPTKTSQVFSTYADNQPGVLIQVFEGERAMTRDNNLLGKFELSGLPPAPRGV
PQIEVTFDIDANGILNVSAVDKSTGKENKITITNDKGRLSKEDIERLVQDAEKYKADDDL
QREKIAAKNGLESYCFNMKSAVEDDKVKDKISESDCSKVLEACNEVLKWLDANQLGEKDE
YEHKQKEVEKVCSPIISKLY----GGAAGCSPGAGPMP---------------------
EAA------------NGA---------G-PTIEEVD
>Odontogammarus_calcarata_GEQQ00000000.1
MRAKSTAVGIDLGTTYSCVGVFQHGKVEIIANDQGNRTTPSYVAFTDTERLIGDAAKNQV
ALNPTNTVFDAKRLIGRKFGDSAVNKDMEHWPFKVVNDGGKPMIGVEYKGESKTFSPEEI
SSMVLTKMREISEAYLGKEVKDAVITVPAYFNDSQRQATKDAGAIAGLNVLRIINEPTAA

AIAYGLDKI--G--S--GEKNILIFDLGGGT-FDVSILTIDDG-IFEVKATAGDTHLGGE
DFDNRLVDHFAQEFKRKYKKDITQNKRALRRLRTACERAKRTLSSSAQASIEIDSLYEGT
DFYTSITRARFEEMCSDLFRGTLEPVEKSLRDAKMDKGQIHEIVLVGGSTRIPKIQKLLQ
DLFNGKELNKTINPDEAVAYGAAVQAAILTGDNSEAVRDLLLLDVAPLSMGIETAGGVMT
ALIKRNTTIPTKTSQVFSTYADNQPGVLIQVFEGERAMTRDNNLLGKFELSGLPPAPRGV
PQIEVTFDIDANGILNVSAVDKSTGKENKITITNDKGRLSKEDIERLVQDAEKYKADDDL
QREKIAAKNGLESYCFNMKSSVEDDKVKDKISESDRSKVLEACNEVLKWLDANQLGEKDE
YEHKQKEVEKVCSPIISKLY---GGGAAGCSPGAGPMQ----------------------
GAA------------NGA---------G-PTIEEVD
>Micruropus_glaber_GEQN00000000.1
MRVKSTAVGIDLGTTYSCVGVFQHGKVEIIANDQGNRTTPSYVAFTDTERLIGDAAKNQV
ALNPTNTVFDAKRLIGRKYGDSTVNKDMAHWPFKVVNDGGKPKIGVEYKGENKTFSPEEI
SSMVLTKMREIAEAYLGKEVKDAVITVPAYFNDSQRQATKDAGAIAGLNVLRIINEPTAA
AIAYGLDKK--G--R--GEKNILIFDLGGGT-FDVSILTIDDG-IFEVKATAGDTHLGGE
DFDNRLVDHFAQEFKRKYKKDITQNKRALRRLRTACERAKRTLSSSAQASIEIDSLHEGT
DFYTSITRARFEEMCSDLFRGTLDPVEKSLRDAKMDKGQIHEIVLVGGSTRIPKIQKLLQ
DFFNGKELNKSINPDEAVAYGAAVQAAILTGDNSEALQDLLLLDVAPLSMGIETAGGVMT
ALIKRNTTIPTKTSQIFSTYADNQPGVLIQVFEGERAMTRDNNLLGKFELSGLPPAPRGV
PQIEVTFDIDANGILNVSAVDKSTGKENKITITNDKGRLSKEDIERMVQDAEKYKADDDQ
QREKIAAKNGLESYCFNMKSTVEDDKLKDKISESDRSKVLEACNEALKWLDANQLGEKDE
YEHKQKEVEKVCSPIISKLY---GGAAAGCTPGSGPMP----------------------
G----------------A---------G-PTIEEVD
>Crypturopus_inflatus_GEPM00000000.1
MKGKSTAVGIDLGTTYSCVGVFQHGKVEIIANDQGNRTTPSYVAFTDTERLIGDAAKNQV
ALNPTNTVFDAKRLIGRKFGDSTVNNDMQHWPFKVVNDGGKPKIGVEYKGENKTFSPEEI
SSMVLTKMREISEAYLGKAVKDAVITVPAYFNDSQRQATKDAGAIAGLNVLRIINEPTAA
AIAYGLDKK--G--R--GEKNILIFDLGGGT-FDVSILTIDDG-IFEVKATAGDTHLGGE
DFDNRLVDHFAQEFKRKFKKDITQNKRAQRRLRTACERAKRTLSSSAQASIEIDSLYEGT
DFYTSITRARFEEMCSDLFRGTLEPVEKSLRDAKMDKGQIHEIVLVGGSTRIPKIQKLLQ
DFFNGKELNKTINPDEAVAYGAAVQAAIMTGDNSEAVQDLLLLDVAPLSMGIETAGGVMT
TLIKRNTTIPTKTSQIFSTYSDNQPGVLIQVFEGERAMTRDNNLLGKFELSGLPPAPRGV
PQIEVTFDIDANGILNVSAVDKSTGKENKITITNDKGRLSKEDIERLVQDAEKYKADDDQ
QREKIASKNGLESYCFNMKSTVEDDKLKDKISESDRSKVLEACNEALKWLDANQLGEKDE
YEHKQKEVEKVCSPIISKLY---GGAAAGCSPGSGPMP----------------------
GSA------------NGA---------G-PTIEEVD
>Gammarus_locusta_FM165078
MRGAGTTVGIDLGTTYSCVGVFQHGKVEIIANDQGNRTTPSFVAFTDTERLIGDAAKNQV
ALNPTNTVFDAKRLIGRKFGDSSINEDIKHWPFKVVNDGGKPKIGVEYKGDSKNFSPEEI
SSMVLTKMRQISEAYLGKTVKDAVITVPAYFNDSQRQATKDAGAIAGLNVLRIINEPTAA
AIAYGLDKK--T--N--GERNILIFDLGGGT-FDVSILTIEDG-IFEVKATAGDTHLGGE
DFDNRLVDHFVQEFKRKFSKDITQNKRAVRRLRTACERAKRTLSYSAQASIEIDSLFEGT
DFYTSITRARFEEMCSDLFRGTLVPVEKSLRDAKMDKGQMHEIVLVGGSTRIPKVQKLLQ
DFFNGKELNKSINPDEAVAYGAAVQAAILAGDNSEAVQDLLLLDVAPLSMGIETAGGVMT
PLIKRNTTIPTKTSQTFSTYADNQPGVLIQVFEGERAMTRDNNLLGKFELSELPPVPRGV
PQIEVTFDVDANGILNVSAVDKSTGKENKITITNDKGRLSKEDIEQMVQDAQKYKADDDQ
QREKIAAKNSLESYCFNIKSTVEDDKLKDKISESDRSKVLESCNEAIKWLDANQLGEKDE
YEHKQKEVEKVCSSIIAKLY---GGRAAGCPPGAGPMP----------------------
GTT------------DGA---------G-PKIEEV-
>Drosophila_melanogaster_mHsc70_1_NP_524063.1
-MPKLPAVGIDLGTTYSCVGVFQHGKVEIIANDQGNRTTPSYVAFTESERLIGDAAKNQV
AMNPNNTIFDAKRLIGRRFDDATVQSDMKHWPFEVFAENGKPRIRVEYKGERKSFYPEEV
SSMVLTKMRETAEAYLGGTVTDAVVTVPAYFNDSQRQATKDAGAIAGLNVLRIINEPTAA
AIAYGLDKQ--G--T--SERNVLIFDLGGGT-FDVSVLTIEDG-IFEVKATAGDTHLGGE
DFDNRLVNHFVQEFQRKHKKDLGQNKRALRRLRTACERAKRTLSSSTQASIEIDSLFEGV
DFYTSVTRARFEELNGDLFRGTMEPVAKALRDAKMDKGQIHDIVLVGGSTRIPKVQRLLQ
DFFNGKELNKSINPDEAVAYGAAVQAAILHGDKSEAVQDLLLLDVTPLSLGIETAGGVMT
TLIKRNTTIPTKQTQIFTTYADNQPGVLIQVFEGERAMTRDNNSLGKFELSAIPPAPRGV
PQVEVTFDIDANGILNVTALEKSTGKENRITITNDKGRLSKEDIERMVNDAEAYRQADEQ
QRDRINAKNQLESYCFQLRSTLDDEHLSSRFSPADRETIQQRSSETIAWLDANQLAERQE
FEHKQQELERICSPIITRLY---QG-A------------G--------------MAPPP
TAGGSNPG---A---TGG---------SGPTIEEVD

Supplementary Data 3. Phylogenetic tree for Fig. 5.

(Fenneropenaeus_chinensis_FJ167398:0.1598061510,
(Cherax_destructor_KR058822:0.0200251495,Pontastacus_leptodactylus_GAFY01019070:0.128250825
6)100/1:0.1166331867,(Drosophila_melanogaster_Hsp70Aa_D__NP_731651.1:0.3230197813,
(((((((((Charybdis_japonica_KM277361:0.0035455610,Scylla_serrata_JQ780845:0.0124863605)0/
0.333:0.0000022591,
((Scylla_paramamosain_EU754021:0.0000024288,Scylla_paramamosain_JX913782:0.0000024288)88.3/
1:0.0035741611,
(Portunus_trituberculatus_FJ527835:0.0035461519,Callinectes_sapidus_DQ663760:0.0000024288)9
6/1:0.0071428675)32.2/0.602:0.0017458459)95.4/1:0.0098812707,
(Chaceon_affinis_KU613091:0.0170910020,
((Xantho_pilipes_KU613099:0.0035443640,Cyanagraea_praedator_KU613100:0.0125262260)0/0.333:0
.0000024287,Segonzacia_mesatlantica_KU613101:0.0035461615)88.6/1:0.0063395550)25.1/0.684:0.
0033962857)96.4/1:0.0142753753,
(((Cardisoma_armatum_KU613075:0.0000024288,Cardisoma_armatum_KU613076:0.0017576236)91.1/1:0
.0052851163,Cardisoma_armatum_KU613077:0.0088900704)93.7/1:0.0092150670,
((((Pachygrapsus_marmoratus_KU613082:0.0052567961,Pachygrapsus_marmoratus_DQ173922:0.00175
06753)0/0.333:0.0000024287,Pachygrapsus_marmoratus_KU613084:0.0000024288)0/0.333:0.00000242
87,Pachygrapsus_marmoratus_KU613083:0.0035010472)0/0.333:0.0000020383,Pachygrapsus_marmorat
us_AM410078:0.0194467776)91.1/1:0.0066622600,Hemigrapsus_nudus_KU613086:0.0076286639)91/1:0
.0093332415)87.8/0.996:0.0072247002)99.4/1:0.0220267494,Homarus_americanus_DQ173923:0.00822
94867)64.2/0.589:0.0029607295,
(Pontastacus_leptodactylus_GAFY01000931:0.0000021207,Astacus_astacus_KU613104:0.0016893532)
99.3/1:0.0190856201)69.2/0.931:0.0047899582,(Cherax_cainii_KR058813:0.0053487848,
(Cherax_quadricarinatus_KR058811:0.0017391311,Cherax_destructor_KR058812:0.0052207193)75.1/
0.712:0.0016344666)93.5/1:0.0099356553)97.2/1:0.0188043510,
(((((Litopenaeus_vannamei_AY645906:0.0000024288,
((Fenneropenaeus_chinensis_AY748350:0.0017111874,Penaeus_monodon_AF474375:0.0034280958)0/0.
333:0.0000024287,Penaeus_monodon_KJ746596:0.0017119920)0/0.333:0.0000024287)0/0.333:0.00000
24287,Penaeus_monodon_EF472918:0.0000024288)0/0.333:0.0000020931,Litopenaeus_vannamei_EF495
128:0.0017108106)77.6/0.921:0.0016967398,
(Marsupenaeus_japonicus_AB520826:0.0034330339,Marsupenaeus_japonicus_EF091692:0.0017142850)
76.9/0.995:0.0017464313)91/0.999:0.0073416827,
(Metapenaeus_ensis_DQ486134:0.0000024288,Metapenaeus_ensis_KJ511266:0.0017432594)99.1/1:0.0
198141076)79.8/0.994:0.0088795599)96.9/1:0.0193643809,
(((Procambarus_clarkii_KU613184:0.0212271121,Pontastacus_leptodactylus_GAFY01000699:0.00386
93349)99.7/1:0.0326835175,
((Cherax_cainii_KR058823:0.0017557328,Cherax_quadricarinatus_KR058821:0.0000025064)30.7/0.7
81:0.0017559552,Cherax_quadricarinatus_HM800921:0.0000026714)95.2/1:0.0167949327)99.8/1:0.0
446814748,((((Eulimnogammarus_verrucosus_JQ003919:0.0010998113,
(Eulimnogammarus_verrucosus_JN704341:0.0000024288,Eulimnogammarus_cyaneus_JN704343:0.000002
4288)77.4/0.991:0.0023801056)91.3/0.999:0.0085704464,Echinogammarus_veneris_GARO01000312:0.
0181136222)95.2/1:0.0207954311,Hyalella_azteca_JQDR01082346:0.0176781017)99.8/1:0.046527990
4,
(((Hyalella_azteca_JQDR01082349:0.0054953262,Hyalella_azteca_JQDR01082354:0.1144645201)79.1
/0.719:0.0062529277,Hyalella_azteca_JQDR01033640:0.1449325522)99.9/1:0.0569960614,
(((((Ommatogammarus_flavus_GEQS00000000.1:0.0066187529,
(Carinurus_bicarinatus_GEPL00000000.1:0.0060306909,
(Oxyacanthus_flavus_GEPB00000000.1:0.0079114445,Odontogammarus_calcarata_GEQQ00000000.1:0.0
069337871)89.5/1:0.0062676814)29.4/0.671:0.0018103322)51.6/0.973:0.0041596382,
(Hyalellopsis_setosa_GEQI00000000.1:0.0125365819,
(Eulimnogammarus_verrucosus_Eve10LT24_1:0.0000020509,
((Eulimnogammarus_verrucosus_Eve10LT24_2:0.0017895056,Eulimnogammarus_verrucosus_Eve10LT24_
3:0.0000027712)77.4/0.974:0.0017852347,
((Eulimnogammarus_cyaneus_Ecy10LT24_1:0.0017847962,Eulimnogammarus_cyaneus_Ecy10LT24_2:0.00
00022849)0/0.333:0.0000020353,
(Eulimnogammarus_cyaneus_Ecy10LT24_3:0.0035657739,Eulimnogammarus_cyaneus_Ecy10LT24_4:0.000
0021541)83.4/0.991:0.0017791429)94.8/1:0.0053751973)70.4/0.943:0.0017866773)82.7/0.992:0.00
18129723)0/0.333:0.0000020657)98.6/1:0.0197247359,Crypturopus_inflatus_GEPM00000000.1:0.022
7339612)65.4/0.972:0.0060045060,Micruropus_glaber_GEQN00000000.1:0.0112567648)51.1/0.9:0.01
22159833,Gammarus_locusta_FM165078:0.0851493618)100/1:0.0689235493)99.7/1:0.0577011337)98/1
:0.0326746182)93.2/1:0.0175512823)95.4/1:0.0275923592,Portunus_trituberculatus_FJ830635:0.0
687178677)95.5/1:0.0450408672,Drosophila_melanogaster_mHsc70_1_NP_524063.1:0.2023937785)71.
2/0.984:0.0454805604)96.1/1:0.0738512142);
